# Development of antitumor biguanides targeting energy metabolism and stress responses in the tumor microenvironment

**DOI:** 10.1038/s41598-021-83708-w

**Published:** 2021-03-01

**Authors:** Takayuki Sakai, Yoshiyuki Matsuo, Kensuke Okuda, Kiichi Hirota, Mieko Tsuji, Tasuku Hirayama, Hideko Nagasawa

**Affiliations:** 1 Laboratory of Pharmaceutical and Medicinal Chemistry, Gifu Pharmaceutical University, Gifu-City, Gifu 501-1196 Japan; 2 Department of Human Stress Response Science, Institute of Biomedical Science, Kansai Medical University, 2-5-1 Shin-machi, Hirakata, Osaka 573-1010 Japan; 3 Laboratory of Bioorganic and Natural Products Chemistry, Kobe Pharmaceutical University, 4-19-1 Motoyama-kita, Higashinada, Kobe 658-8558 Japan

**Keywords:** Drug discovery, Medicinal chemistry, Drug discovery and development

## Abstract

To develop antitumor drugs capable of targeting energy metabolism in the tumor microenvironment, we produced a series of potent new biguanide derivatives via structural modification of the arylbiguanide scaffold. We then conducted biological screening using hypoxia inducible factor (HIF)-1- and unfolded protein response (UPR)-dependent reporter assays and selective cytotoxicity assay under low glucose conditions. Homologation studies of aryl-(CH_2_)_n_-biguanides (n = 0–6) yielded highly potent derivatives with an appropriate alkylene linker length (n = 5, 6). The *o*-chlorophenyl derivative **7l** (n = 5) indicated the most potent inhibitory effects on HIF-1- and UPR-mediated transcriptional activation (IC_50_; 1.0 ± 0.1 μM, 7.5 ± 0.1 μM, respectively) and exhibited selective cytotoxicity toward HT29 cells under low glucose condition (IC_50_; 1.9 ± 0.1 μM). Additionally, the protein expression of HIF-1α induced by hypoxia and of GRP78 and GRP94 induced by glucose starvation was markedly suppressed by the biguanides, thereby inhibiting angiogenesis. Metabolic flux and fluorescence-activated cell sorting analyses of tumor cells revealed that the biguanides strongly inhibited oxidative phosphorylation and activated compensative glycolysis in the presence of glucose, whereas both were strongly suppressed in the absence of glucose, resulting in cellular energy depletion and apoptosis. These findings suggest that the pleiotropic effects of these biguanides may contribute to more selective and effective killing of cancer cells due to the suppression of various stress adaptation systems in the tumor microenvironment.

## Introduction

Metabolic reprogramming has been recognized as an emerging hallmark of cancer and has attracted attention as a tumor-selective target for the development of antitumor agents^1,2^. Unlike normal cells, tumor cells were widely believed to use glycolysis as a major source of ATP production instead of oxidative phosphorylation (OXPHOS) even under normoxic conditions due to their impaired mitochondrial activity (the Warburg effect)^3^. However, recent investigations have found intact mitochondria in most tumor cells, and have confirmed that these play a very important role in tumor growth, particularly when tumors rely on OXPHOS for bioenergetic and biosynthetic processes^4–6^. Furthermore, cancer mitochondria can flexibly switch between glycolysis and OXPHOS in order to meet the challenge of high demands for energy and biosynthesis inherent to their survival^7^. The upregulation of OXPHOS has been well studied in several types of cancers, and several subtypes of cancers, especially those with glycolytic dysfunction, are particularly sensitive to OXPHOS inhibitors^8^. Accordingly, therapeutic strategies that target mitochondrial metabolism, such as OXPHOS inhibition, are considered promising and are attracting a great deal of attention. Currently, the development of anticancer drugs that target mitochondrial metabolisms has been actively pursued, and multiple preclinical or clinical trials have been conducted to explore the potential of mitochondrial metabolism inhibition as a new cancer treatment^9–14^. Having said that, only a limited number of OXPHOS inhibitors have been tested in clinical trials, and so far none have advanced to the late-stages of clinical development^8^. Further efforts are needed to achieve clinical application of OXPHOS inhibitors for cancer treatment.

Biguanides, such as phenformin (**1**) and metoformin commonly used to treat type 2 diabetes, are strong inhibitors of the mitochondrial respiratory complex-I^15,16^. Numerous preclinical studies have been conducted to evaluate the application of biguanides for antitumor treatments exploiting the metabolic vulnerability of neoplastic cells^11,17–19^. In addition, clinical trials have shown that various forms of cancer exhibit significant clinical responses to metformin^18,20^. Phenformin shows stronger OXPHOS inhibition and antidiabetic effect than metformin, but its clinical use has been discontinued due to the high incidence of lactic acidosis, a major side effect of biguanides. However, phenformin is expected to have a higher anti-tumor effect than metformin due to its better bioavailability^19^, and in fact, the superiority of phenformin has been reported in xenograft models^21^. Furthermore, biguanide derivatives have been shown to reduce unfolded protein response (UPR)-dependent transactivation, activate AMP-activated protein kinase (AMPK), and cause selective cytotoxicity in glucose-deprived tumor cells. Phenformin is also considerably potent than metformin with respect to these biological effects^22,23^. Therefore, we have focused on the arylbiguanide scaffold and developed new arylbiguanides with higher activity than phenformin by screening based on selective cytotoxicity during glucose deprivation^24^. These arylbiguanide derivatives led to significant suppression of hypoxia-induced factor-1 (HIF-1)- and UPR-dependent transcriptional activation, the expression of various ER stress-related proteins and c-Myc, as well as angiogenesis^24–27^. Cancer cells have various adaptive response systems, including HIF-1 and UPR signaling pathways, which enable them to survive under hypoxia and low nutrition stresses in the tumor microenvironment (TME)^28^. Accordingly, biguanide derivatives have been suggested as promising drug seeds for antitumor agents able to target TMEs, in addition to being candidates for mitochondrial-targeting anticancer agents.

Our previous study on the structural modification of the aryl ethylamine scaffold revealed that strong basic components, such as biguanides and guanidine, are necessary to inhibit HIF-1- and UPR-dependent transcriptional activation and selective cytotoxicity under glucose deprivation. In the present study, we performed structural modifications based on aryl-based biguanide scaffolds in order to develop antitumor drugs targeting TME and mitochondrial energy metabolisms. In addition, we analyzed the effect of these new biguanides on the energy metabolism of cancer cells and investigated mechanisms of TME-selective cytotoxicity.

## Results and discussion

### Structural modifications and synthesis

**Figure 1:**
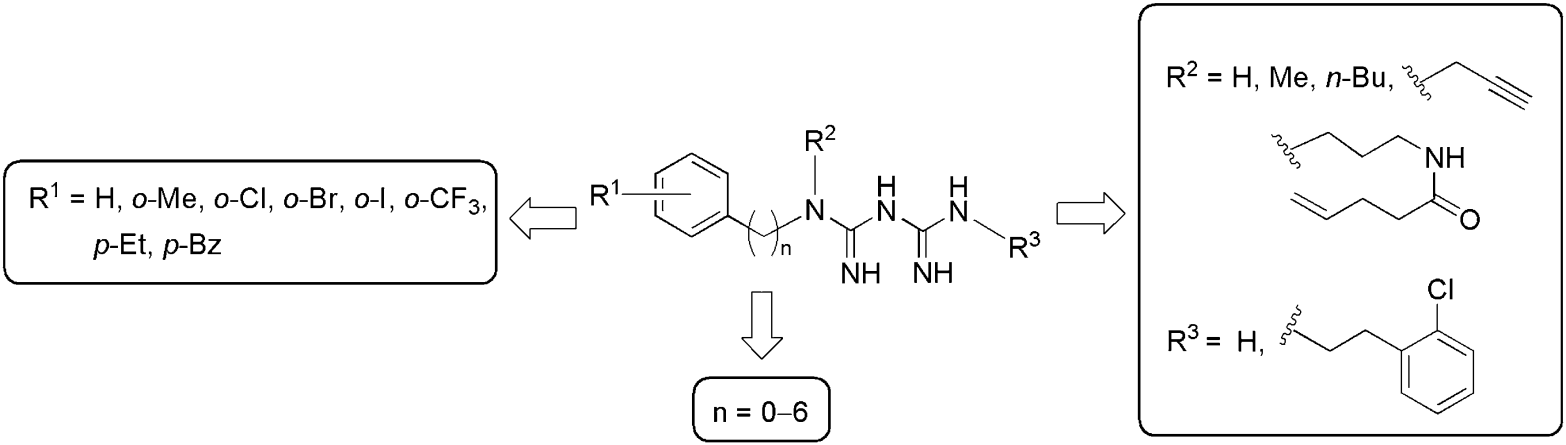
Structural modifications based on the aryl biguanide scaffold.

**Figure 2:**
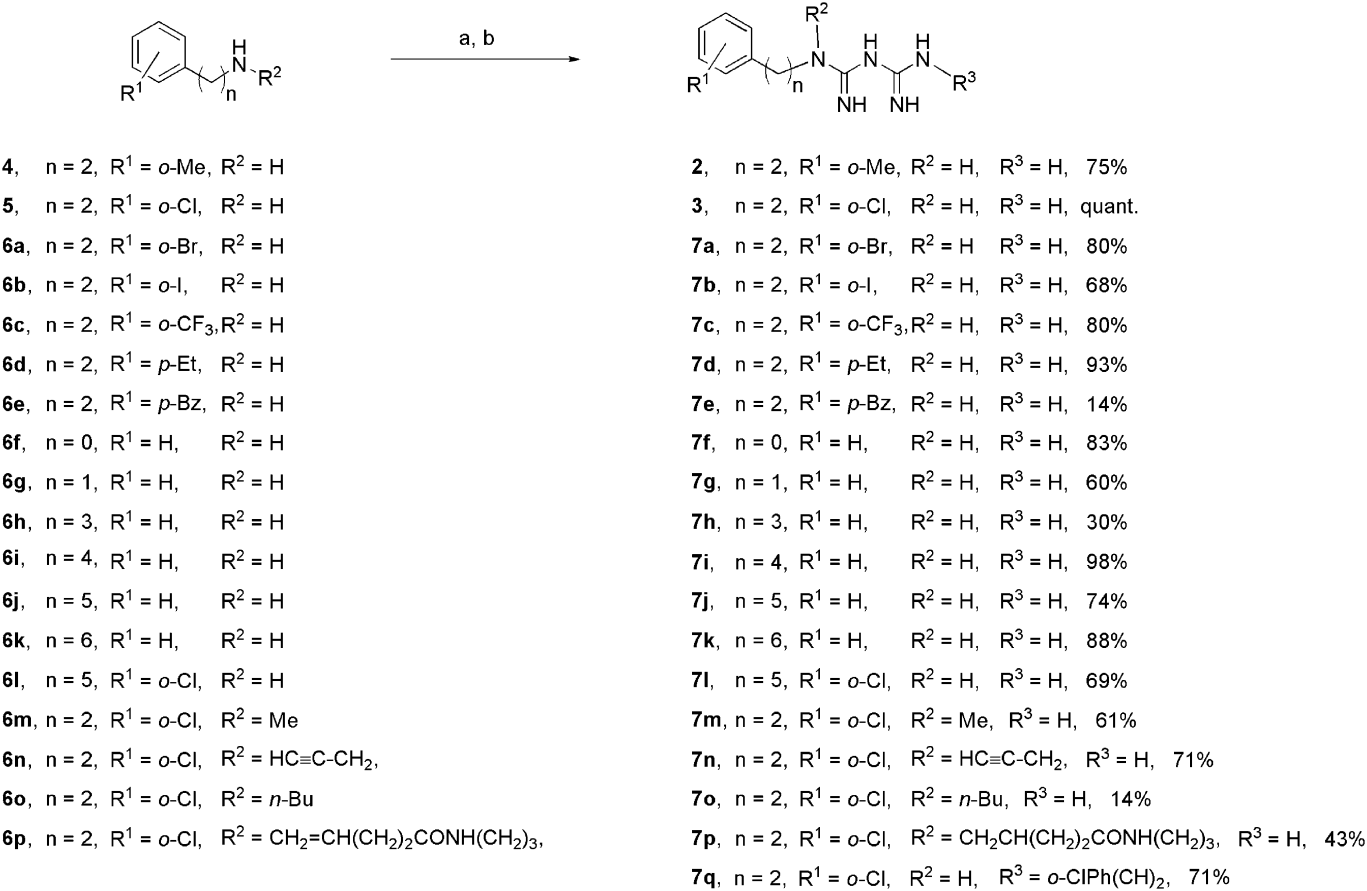
Synthesis of a series of biguanides via microwave-assisted reactions. Reagents and conditions: (a) dicyandiamide, TMSCl, CH_3_CN, 130 °C or 150 °C under microwave irradiation, 5–15 min; (b) *i*PrOH, 125 °C under microwave irradiation, 1 min.

In a previous structure–activity relationship study, we found that the introduction of a chloro or methyl group at the o-. *m*- or *p*-position of the phenyl group greatly enhanced the activity of phenformin (**1**). We also found that *o*-substituted derivatives **2** and **3** exhibited the strongest cytotoxicity and selectivity under low glucose conditions^20^. However, their inhibitory effects on HIF-1- and UPR-mediated transcriptional activation showed little difference depending on the position of the substituent. Therefore, as shown in Fig. 1, structural modifications were performed based on the aryl biguanide scaffold, including the introduction of substituents to the aromatic ring, methylene homologation of the alkylene linker, and modification of the biguanide moiety. Biguanide derivatives were efficiently obtained from their corresponding primary or secondary amines Supplementary Information, Figure S1 and dicyandiamide within 5–15 min using a microwave assisted reaction following Mayer's method^29^ (Fig. 2).

### Biological evaluation

**Figure 3:**
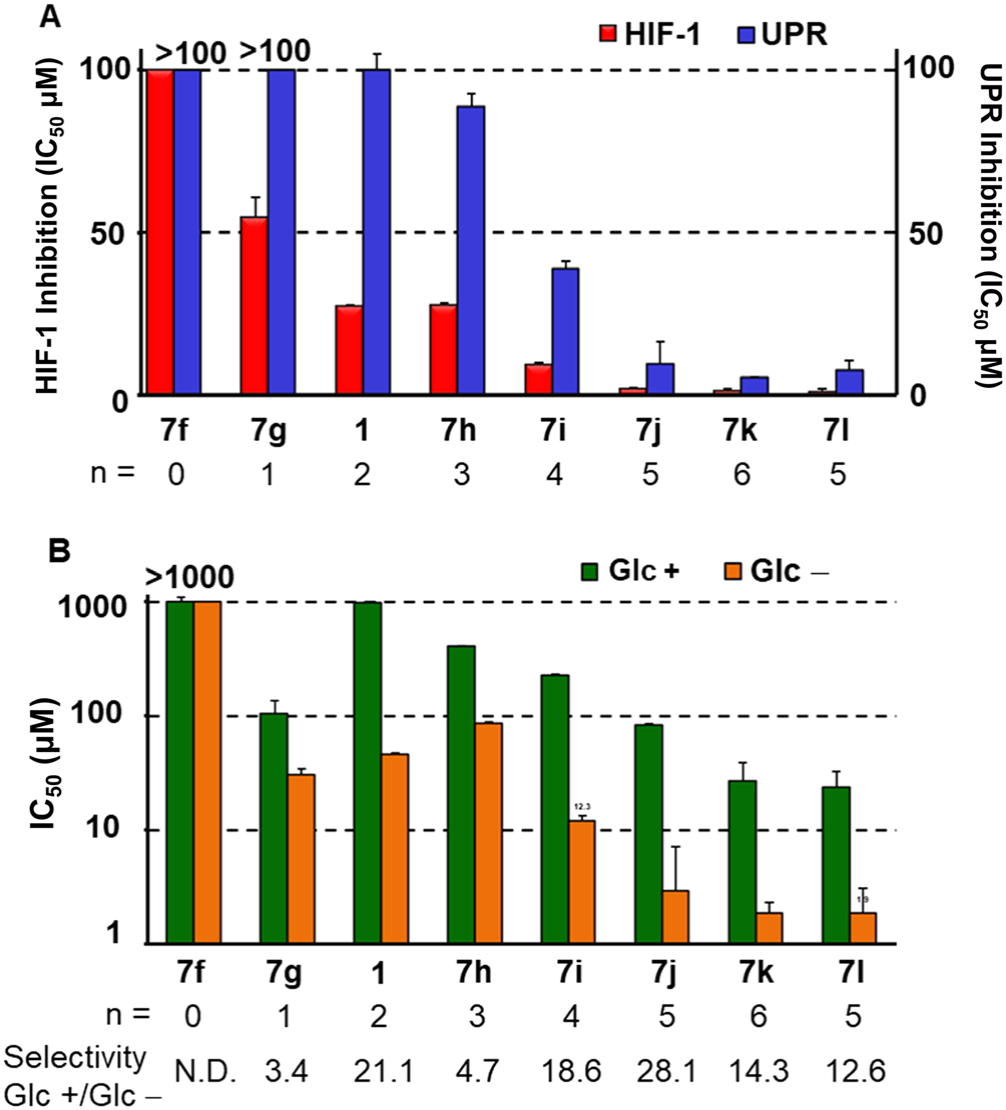
(**A**) IC_50_ for HIF-1- and UPR-dependent transcriptional activation of biguanides with different alkylene linker chain lengths. HEK293 p2.1 #3 and HEK293 GRP78 #85 cells were treated with **1** and **7f**–**7l** for 24 h under hypoxic or 0.3 mM 2-DG conditions, respectively. (**B**) IC_50_ of cytotoxicity on HT29 by treatment with biguanides. Cells were treated with **1** and **7f**–**7l** for 48 h in the presence (Glc +) or absence (Glc −) of glucose.

As a first-stage screening, cell-based luciferase reporter assays were undertaken in response to hypoxic and glucose deficiency stress. The luciferase assay was performed as reported previously^24^ using our stable cell lines of HEK293 transfected with the HIF-1-responsive luciferase reporter plasmid p2.1^30,31^ or the pGRP78pro160-luc plasmid containing the human glucose-regulated protein (GRP) 78 promoter with an ER stress response element^24,32^. Subsequently, a 3-(4,5-dimethylthiazol-2-yl)2,5-diphenyltetrazolium bromide (MTT) assay was performed on HT-29 colorectal adenocarcinoma cells in glucose-containing and glucose-free media in order to assess selective cytotoxicity under glucose-deprived conditions. As shown in Table 1, when bromine (**7a**) or iodine (**7b**) was used as the halogen substituent at the *o*-position, the inhibitory effects on HIF-1- and UPR-mediated transcription activations were stronger, but the selective cytotoxicity with low glucose was weaker than in the case of the chlorine substituent (entries 3–5). Our SAR studies of biguanide derivatives found that upsilon steric parameters^33^ correlated with cytotoxicity under low glucose conditions^24^. Considering the upsilon parameters of substituents 0.55(Cl), 0.65(Br), 0.78(I), CF_3_(0.91), Me(0.52), and Et(0.56), it was found that the cytotoxicity under glucose depletion (Glc −) was enhanced by the attachment of halogens or relatively small alkyl groups with upsilon values between 0.52 and 0.91 to the aromatic ring regardless of their substitution position (entries 1–7). On the other hand, more bulky substituents, such as the benzoyl group (**7e**) (entry 8) and *t*-Bu [υ; 1.24, IC_50_ (Glc −); 23.4 μM]^24^ in the para position, caused significant reductions in the cytotoxicity (Glc −). In the homologous series of aryl-(CH_2_)_n_–biguanide (n = 0–6), hydrophobicity increased and biological activity became stronger with increasing alkylene linker length. (entry 9–14, Fig. 3). In particular, **7j** (n = 5), **7k** (n = 6) and the *o*-chloro phenyl analogue **7l** (n = 5) showed potent inhibitory effects on both HIF-1- and UPR-mediated transcriptional activation and were highly selectively cytotoxic under glucose-deprived conditions (entry 13–15).

**Table 1:** Inhibitory activities for HIF-1- and UPR-mediated transcriptional activation and cytotoxicity of biguanides.

		
Entry	Compd	n	R^1^	R^2^	R^3^	IC_50_ of transactivation (μM)		IC_50_ of cytotoxicity (μM)^c^		Selectivity^d^ Glc + /Glc −	Rm	c log *D*^f^ (pH 7.4)
						HIF-1^a^	UPR^b^	Glc +	Glc −			
1	**1**	2	H	H	H	27.4 ± 6.1^g^	107.8 ± 9.4^g^	972.6 ± 95.0^g^	46.2 ± 9.2^g^	21.1^g^	− 0.010	− 6.64^g^
2	**2**	2	*o*-Me	H	H	6.0 ± 0.1^g^	87.9 ± 4.9^g^	403.1 ± 31.2^g^	7.2 ± 3.5^g^	56^g^	0.092	− 6.50
3	**3**	2	*o*-Cl	H	H	5.8 ± 0.3^g^	37.4 ± 3.1^g^	350.4 ± 11.9^g^	5.2 ± 0.7^g^	67.4^g^	0.15	− 6.39
4	**7a**	2	*o*-Br	H	H	3.1 ± 0.4	29.9 ± 9.4	217.6 ± 11.2	7.3 ± 0.5	29.8	0.41	− 6.32
5	**7b**	2	*o*-I	H	H	4.6 ± 0.4	23.6 ± 1.4	230.3 ± 48.8	14.1 ± 1.1	16.3	0.48	− 6.16
6	**7c**	2	*o*-CF_3_	H	H	5.0 ± 0.3	89.2 ± 4.5	316.6 ± 10.6	15.8 ± 0.7	20	N.D	− 6.02
7	**7d**	2	*p*-Et	H	H	3.0 ± 0.3	41.3 ± 6.8	80.3 ± 4.4	6.1 ± 1.1	13.2	0.46	− 6.52
8	**7e**	2	*p*-Bz	H	H	4.6 ± 0.6	92.1 ± 11.4	217.5 ± 13.8	28.7 ± 2.7	7.6	0.63	− 5.94
9	**7f**	0	H	H	H	> 100	> 100	> 1000	> 1000	N.D	N.D	− 4.83
10	**7g**	1	H	H	H	54.7 ± 8.6	126.6 ± 19.8	105.3 ± 10.1	31.0 ± 7.8	3.4	− 0.036	− 6.73
11	**7h**	3	H	H	H	27.7 ± 6.1	86.8 ± 22.3	407.4 ± 80.8	86.9 ± 9.4	4.7	0.28	− 6.57
12	**7i**	4	H	H	H	9.4 ± 0.4	38.8 ± 4.2	228.8 ± 13.8	12.3 ± 2.3	18.6	0.43	− 6.49
13	**7j**	5	H	H	H	2.0 ± 0.1	9.6 ± 0.3	84.3 ± 11.7	3.0 ± 1.1	28.1	0.58	− 6.40
14	**7k**	6	H	H	H	1.3 ± 0.2	5.5 ± 0.6	27.1 ± 1.3	1.9 ± 0.3	14.3	0.80	− 6.30
15	**7l**	5	*o*-Cl	H	H	1.0 ± 0.1	7.5 ± 0.1	24.0 ± 1.4	1.9 ± 0.1	12.6	0.57	− 6.05
16	**7m**	2	*o*-Cl	Me	H	29.0 ± 3.0	116.3 ± 13.1	224.7 ± 72.9	43.0 ± 7.9	5.2	0.36	− 4.70
17	**7n**	2	*o*-Cl		H	48.5 ± 2.4	148.2 ± 31.7	94.1 ± 9.3	24.2 ± 5.6	3.9	0.63	− 4.20
18	**7o**	2	*o*-Cl	*n*-Bu	H	40.7 ± 6.4	96.6 ± 19.6	42.4 ± 14.6	21.7 ± 4.1	2.0	0.55	− 4.05
19	**7p**	2	*o*-Cl		H	> 100	> 100	> 300	> 300	N.D	− 0.010	− 3.76
20	**7q**	2	*o*-Cl	H		N.D	N.D	17.6 ± 1.0	18.2 ± 1.7	1.0	N.D	− 5.09

^a^IC_50_, 50% inhibitory concentration (mean ± SD, n = 3). Determined by luciferase assay using HEK293 p2.1 #3 cells following 24 h of drug treatment under hypoxia.

^b^Determined by luciferase assay using HEK293 GRP78 #85 cells following 24 h of drug treatment with 0.3 mM 2-DG.

^c^Determined by MTT assay on HT29 cells following 48 h of drug treatment in the presence (Glc +) or absence (Glc −) of glucose.

^d^Calculated by IC_50_ (Glc +)/IC_50_ (Glc −).

^e^Rm_,_ hydrophobic parameter^34,35^ calculated as described in the “Experimental section” section.

^f^Calculated by Accord for Excel ver. 7.1.5 (Accelrys Software, Inc., Santa Clara, CA, USA).

^g^Data from ref. ^24^. *N.D.*, not determined. Concentration response curves for biguanide **7l** can be found in Figure S3.

We evaluated hydrophobicity for biguanides by Rm values calculated from TLC-relation factor (Rf) by using octylsililated silicagel plate^34,35^ and calculated log D (clog D), distribution coefficients at physiological pH. According to the regression analyses of the homologation series data (Figure S2A), there was a very good positive correlation between the methylene number of the alkylene linker and the hydrophobicity parameters (Rm; R^2^ = 0.9758, clog D; R^2^ = 0.9971) and between Rm and clog D values (R^2^ = 0.9699). The Rm value of **7l** was almost equal to that of **7j** with the same linker length (n = 5) on the fitted regression line, but its clog D was far off the regression line to the hydrophobic side. Therefore, we analyzed the correlation of the IC_50_ for each biological activity to Rm values for mono-substituted biguanides (entries 1–15). Interestingly, the IC_50_ of inhibitory activity of UPR-mediated transcriptional activation correlated well with the Rm value (R^2^ = 0.8653, Figure S2B). However, the IC_50_ for HIF-1-mediated transcriptional activation and cytotoxicity (G + and G −) did not correlate with the Rm in either case (R^2^ = 0.3–0.7, Figure S2B–S2D). Since anti-diabetic biguanides are positively charged at neutral pH and have low hydrophobicity, they require organic cation transporters (OCTs) to facilitate their uptake into cells^36^. On the other hand, phenformin, which is more hydrophobic than metformin, was also reported to directly permeate the cell membrane^37^. In contrast, most of the biguanides shown here are much more hydrophobic than phenformin as predicted by their Rm values, and thus are more likely to cross the cell membrane by direct diffusion. Therefore, hydrophobic factors are considered to be of significance in these biological activities targeting the cancer microenvironment. In this connection, Bridges et al*.* reported a remarkable finding^38^. Namely, bis-substituted biguanides or those with a direct conjugated phenyl group inhibited isolated complex I, but did not inhibit mitochondrial respiration because they were not taken up by mitochondria regardless of hydrophobic parameters. This suggests a selective transporter across the mitochondrial inner membrane. As for our disubstituted biguanides with substituents at N1 position, **7m**, **7n**, and **7o**, shown in Table 1 (entries 16–18), were not as potent, but exhibited HIF-1 and UPR inhibition and low glucose-selective cytotoxicity comparable to those of phenformin (**1**). These results indicate that the selective cytotoxicity of new biguanides is not solely attributed to mitochondrial damage.

Furthermore, compounds **2**, **3** and **7l**, which showed strong activity, were evaluated for selective cytotoxicity under glucose deprivation in A549 lung cancer and U87-MG glioma, both of which have been suggested to be more dependent of glycolytic processes for their energy metabolism than HT29^39,40^ (Table 2). Among these compounds, **7l** showed the most potent cytotoxicity under glucose deprivation in all tested cell lines. It is also noteworthy that **7l** is effective against a wide variety of cancer cells and exhibits strong cytotoxicity, even against the highly malignant U87MG glioma, due to mitochondrial dysfunction and metabolic reprograming^41^.

**Table 2:** Cytotoxicity of **2**, **3** and **7l **on HT29, A549 and U87MG cells.

Compd.	Cell	IC_50_ of cytotoxicity (μM)		Selectivity Glc +/Glc −
		Glc +	Glc −	
**2**	HT29	403.1 ± 31.2^a^	7.2 ± 3.5^a^	56^a^
	A549	232.5 ± 13.9	13.2 ± 1.9	17.6
	U87MG	214.6 ± 2.7	37.1 ± 8.2	5.8
**3**	HT29	350.4 ± 11.9^a^	5.2 ± 0.7^a^	67.4^a^
	A549	127.2 ± 27.1	4.4 ± 0.3	28.9
	U87MG	193.3 ± 22.6	21.8 ± 1.8	8.9
**7l**	HT29	24.0 ± 1.4	1.9 ± 0.1	12.6
	A549	17.4 ± 4.3	2.6 ± 1.4	6.7
	U87MG	23.8 ± 1.4	9.4 ± 4.5	2.5

IC_50_ (mean ± SD, n = 3) was determined by MTT assay on each cell following 48 h of drug treatment in the presence (Glc +) or absence (Glc −) of glucose.

^a^Data from ref. ^24^. Concentration response curves for biguanide **2**, **3** and **7l** on A549 and U87MG and **7l** on HT29, A549 and U87MG can be found in Figures S3, S4 and S5.

**Figure 4:**
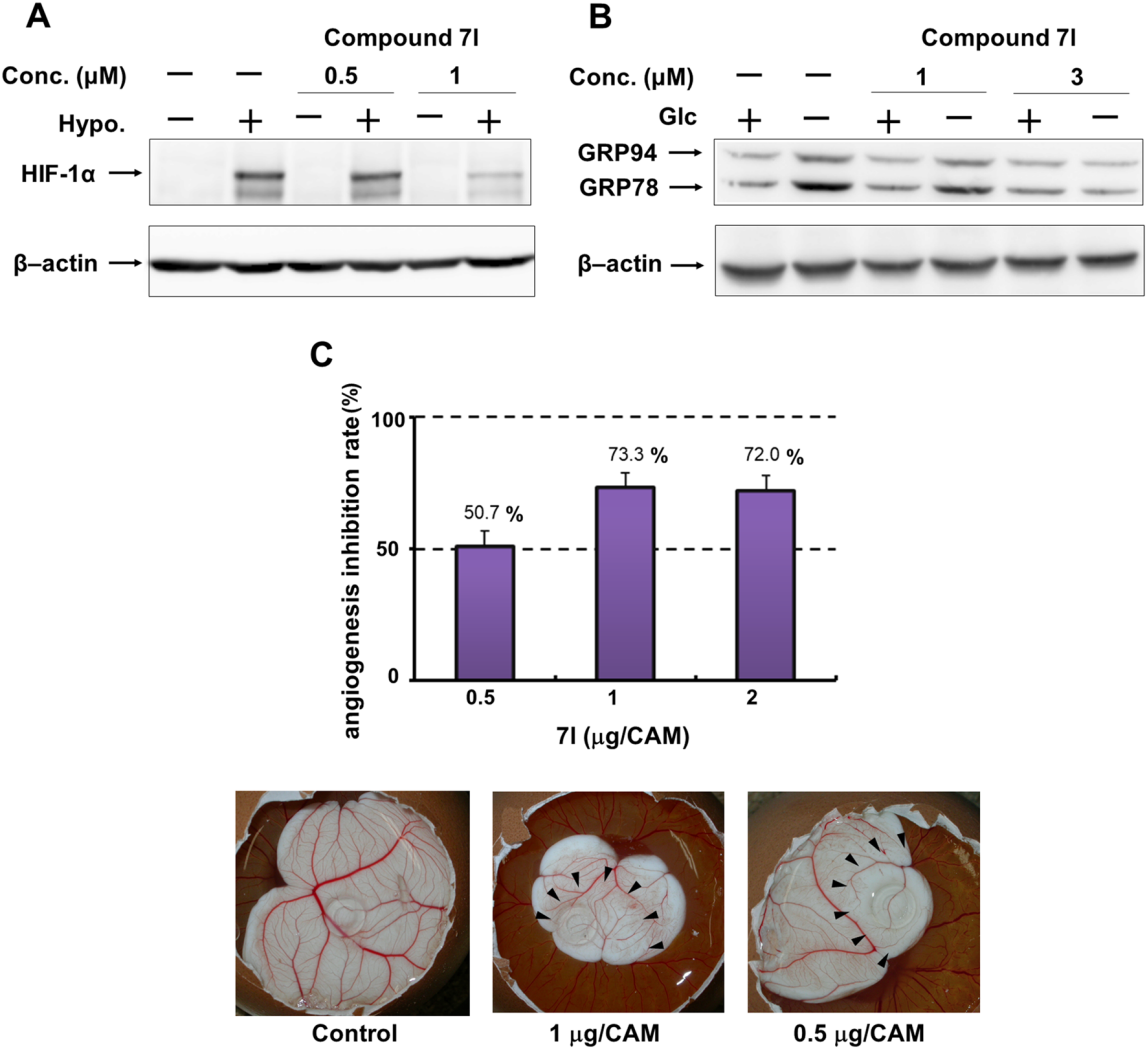
Inhibition effect of **7l** on protein expressions of HIF-1α induced under hypoxia or GRP78 and GRP94 by glucose deprivation. Immunoblot analysis of HIF-1α (**A**), GRP78 and GRP94 (**B**) (See also Fig. S7). (**A**) HT29 cells were incubated with compound **7l** for 4 h under normoxic (20% O_2_) (Hypo. −) or hypoxic (1% O_2_) (Hypo. +) conditions. (**B**) HT29 cells were treated with compound **7l** for 24 h in normal (2000 mg/L glucose, Glc +) or glucose-free (Glc −) medium. (**C**) Antiangiogenic effects of compound **7l** in CAM assay. The CAMs of 4-day-old chick embryos were treated with compounds for 2 days. Antiangiogenesis ratios of **7l**, calculated from the following formula: Antiangiogenesis ratio (%) = (1 − [control point/drug point]) × 100. Eight to ten eggs were used in total for each condition. Pictures of **7l**–treated CAM show avascular zone around the silicon ring where the test compound was administered. The area surrounded by arrowheads indicates the hypovascular region.

The above screening led to the most promising compound **7l** being selected for examination of its effect on the expression of HIF-1α and UPR-mediated protein expression. As shown in Fig. 4A and B, compound **7l** clearly suppressed HIF-1α protein expression induced by hypoxia at concentrations of 1 μM, one-tenth of the effective concentration of compounds **2** and **3**^24^. Additionally, compound **7l** completely inhibited the induction of protein expression in GRP78 and GRP94, the major regulators of UPR, by glucose deprivation at concentrations of 3 μM.

Phenformin and metformin are known to inhibit angiogenesis by reducing the production of angiogenesis-related proteins^17^. The in vivo inhibitory effect of such compounds on angiogenesis can be evaluated by chick chorioallantoic membrane (CAM) assay^42,43^. We previously demonstrated that phenformin (**1**) and **2** inhibited neovascularization in chick embryos at 5 μg/CAM and 2 μg/CAM, respectively^24^, but here found that **7l** significantly inhibited angiogenesis at a much lower dose, 0.5 μg/CAM, as shown in Fig. 4C.

### Analysis of energy metabolism

**Figure 5:**
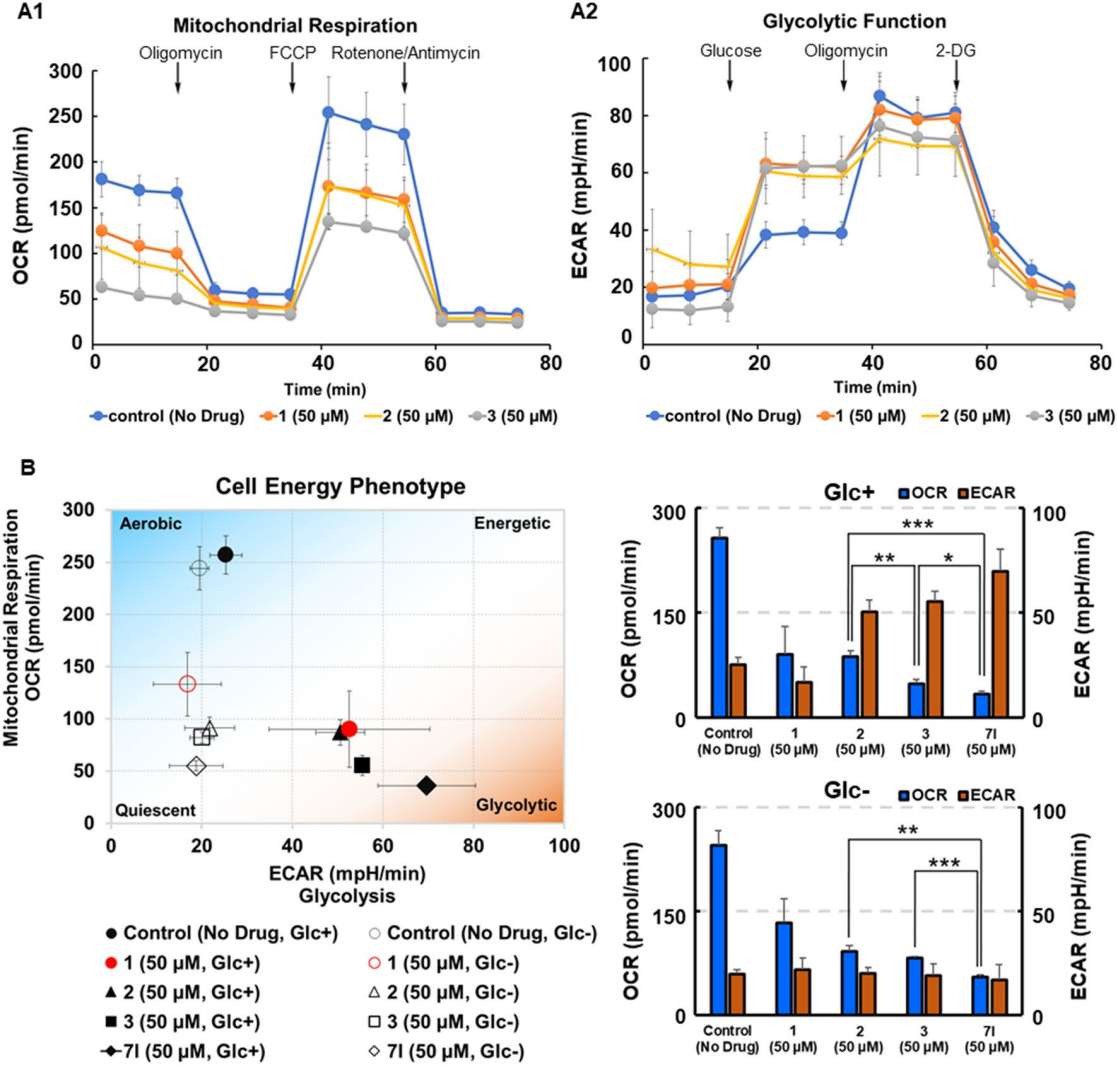
Influence of biguanides on the energy metabolism of HT29 in the presence or absence of glucose. (**A1**) HT29 cells were treated with 50 μM of **1, 2**, and **3** in assay medium (glucose, 10 mM) for 1 h, and then the OCR was measured over time using an XFp extracellular flux analyzer while sequentially injecting oligomycin (1.5 µM), FCCP (2 µM) and rotenone/antimycin A (0.5 µM each). (**A2**) HT29 cells were treated with 50 μM of **1**, **2**, and **3** in glucose-free assay medium for 1 h, and then the ECAR was measured over time while sequentially injecting glucose (10 mM), Oligomycin (1 μM) and 2-DG (50 mM). (**B**) Cell energy phenotype profile of HT29 cells treated with biguanides. After the treatment with 50 μM of **2**, **3**, and **7l** in glucose-containing (Glc +) or non-glucose (Glc −) assay medium for 1 h, OCR and ECAR were measured by XFp extracellular flux analyzer. The differences in OCR values between each compound treatment were statistically significant (*p < 0.05; **p < 0.005; ***p < 0.0005). Each data represents mean ± SD of triplicate experiments.

Birsoy et al. demonstrated that the sensitivity of cancer cells to biguanides, which are OXPHOS inhibitors, is enhanced under low glucose conditions and that OXPHOS is a major pathway required for optimal cancer cell proliferation under such conditions^44^. Therefore, in order to investigate the effects of new biguanides on the energy metabolism of tumor cells, we evaluated mitochondrial function using the oxygen consumption rate (OCR) as an indicator and glycolytic function using extracellular acidification rate (ECAR) in living HT29 cells under normal and low glucose conditions using the XFp extracellular flux analyzer^45,46^. ECAR is primarily a measure of lactate production and can be equated to glycolytic rate. We measured changes in OCR and ECAR of HT-29 cells in the absence or presence of biguanides **1**, **2** and **3** in response to the sequential addition of the following mitochondrial inhibitors: the ATP synthase inhibitor oligomycin, the mitochondrial uncoupler FCCP, and the complex I/III inhibitors rotenone and antimycin A. As shown in Fig. 5A mitochondrial basal and maximal respirations were more strongly inhibited by **2** than by** 1** and **3** at concentrations of 50 μM, while ECARs derived from the glycolysis increased to compensate for OXPHOS. The *o*-chloroaryl derivatives **3** and **7l** significantly suppressed OCR and enhanced ECAR in HT-29 cells relative to **1** and **2** at concentrations of 50 μM in the presence of glucose (Fig. 5B). On the other hand, both OCR and ECAR were suppressed in the glucose-free medium. In particular, **7l** reduced the OCR completely (33.4 pmol/min) until the level of non-mitochondrial oxygen consumption in the presence of glucose. Correlations between ECAR and OCR of cells treated with various biguanides in both normal and glucose deprived conditions are shown in the energy phenotype profile diagram (Figs. 5B and S6). The responses of various tumor cell lines to energy demands under each culture condition reflects their metabolic potential. U87MG gliomas exhibited a Warburg phenotype of low OCR and high ECAR compared to other cells under normal conditions^40^, and the metabolisms of all cell lines were reprogrammed toward a more oxidative phenotype under conditions of glucose deprivation^41^. The energy phenotype profile diagram shows that the presence of biguanides led to a significant reduction in OCR both in the presence and absence of glucose in all cell lines; the order of the strength of inhibition was **7l** > **3** > **2** > **1**. In total, of the compounds examined, **7l** inhibited OXPHOS most potently, such that the inhibitory effect was observed even at a concentration of 25 μM. In the presence of glucose, stronger OXPHOS inhibitor caused greater increases in ECAR. In addition, when energy production by mitochondrial respiration is inhibited by OXPHOS inhibitor, the glycolytic system is normally activated to compensate for the lack of energy. The limited availability of oxygen also enhances the glycolytic system via activation of HIF-1 signaling, resulting in lactic acidosis as a side effect. On the other hand, the present biguanide strongly inhibits protein expression and transcriptional activation of HIF-1α at lower concentrations then OXPHOS inhibition, which may prevent acidosis. Furthermore, recently, Minamishima et al*.*, proposed a hopeful strategy to avoid acidosis by activating HIF-1 with a prolyl hydroxylase domain-containing protein 2 (PHD2) inhibitor^47^. Activation of HIF-1 signaling by PHD2 inhibitor would enhance the Cori cycle, which activate gluconeogenesis to reduce lactate. In cancer treatment, metformin has been reported to not cause acidosis or hypoglycemia in non-diabetic patients, but the cancer treatment effects and side effects of phenformin have not been well studied. New compound **7l** showed potent low glucose-selective cytotoxicity, HIF-1 inhibition, and UPR inhibition, all of which were about 20-fold higher than phenformin, but no OXPHOS inhibition was observed at doses effective for these biological effects. These suggest that the adverse effect, lactic acidosis, may be alleviated with appropriate dosing or combination therapy^19^.

### Fluorescence-activated cell sorting (FACS) analysis of cell death

**Figure 6:**
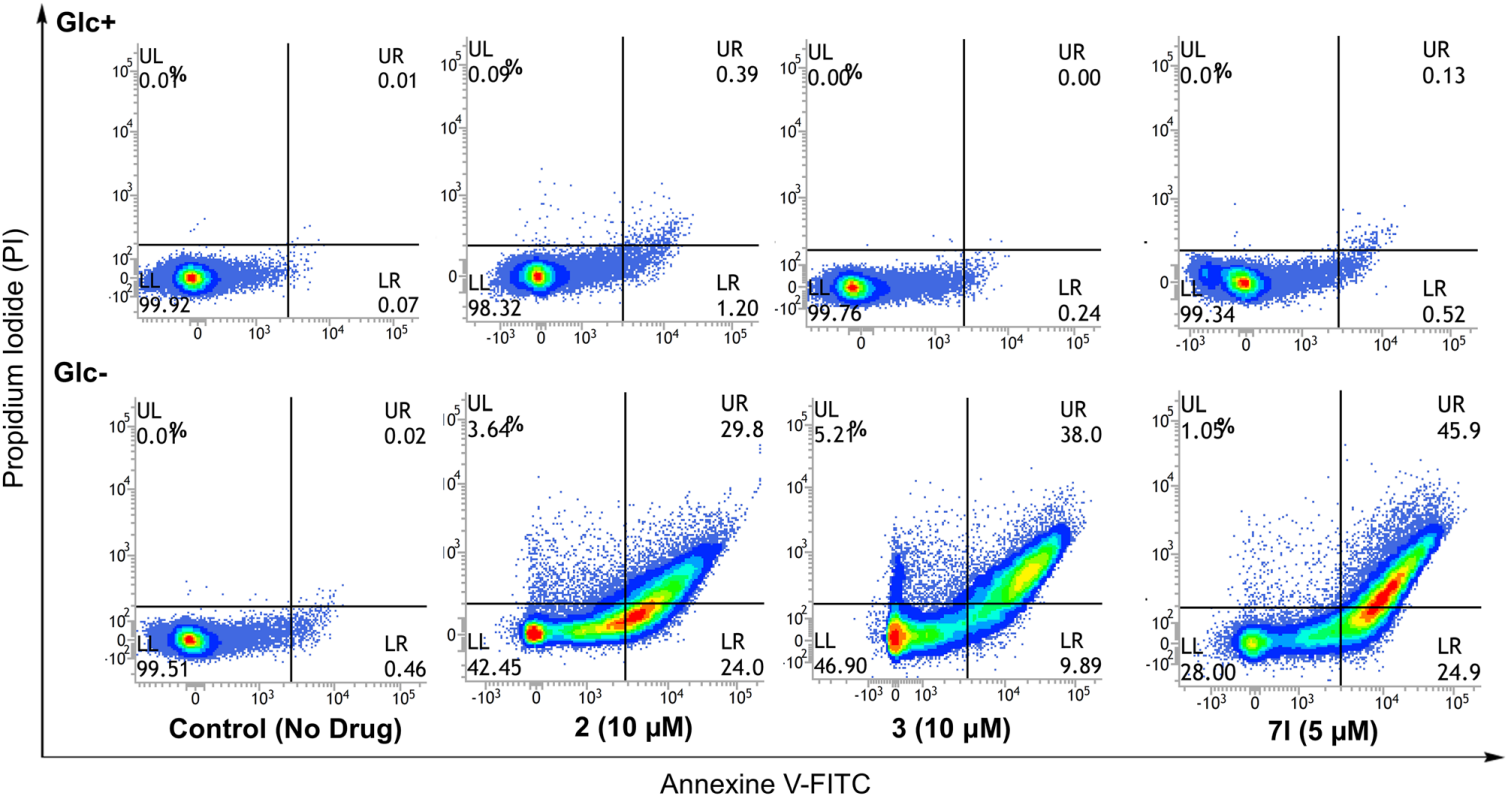
FACS analysis of apoptosis in HT29 treated with **2, 3** and **7l** for 48 h in normal (2000 mg/L glucose, Glc +) or glucose-free (Glc −) medium.

It has been reported that the antiproliferative activity of biguanides is mediated by the induction of apoptosis in breast cancer cells and is markedly enhanced under glucose-deficient conditions^48^. To evaluate the cell death of HT29 cells via biguanide treatment in media with variable glucose levels, flow cytometric analysis was performed by double staining with Annexin V-FITC and propidium iodide (PI). HT29 cells were treated with compounds **2, 3**, and **7l** at concentrations of 10 µM, 10 µM, and 5 µM, respectively, for 48 h and subjected to FACS analysis. The total ratios of early (Annexin V-FITC+ /PI−) and late (Annexin V-FITC+/PI+) apoptosis induced by compounds **2, 3**, and **7l** in the low glucose medium were 53.8%, 47.9%, and 70.8%, respectively (Fig. 6). On the other hand, minimal cell death was observed in cells exposed to the compounds in the presence of glucose, suggesting that biguanide treatment selectively promoted apoptosis at low levels of glucose. These results are consistent with previous reports by Ben Sahra et al*.*, who found that energy depletion with metformin and 2-deoxy-d-glucose (2-DG) induced apoptosis in prostate cancer cells^49^.

## Conclusions

To develop therapeutic agents targeting energy metabolism in the tumor microenvironment, we developed a series of potent new biguanide derivatives via structural modification of the aryl biguanide scaffold and screening using HIF-1- and UPR-dependent reporter assays and selective cytotoxicity assays under low glucose conditions. Homologation studies of aryl-(CH_2_)_n_-biguanides (n = 0–6) yielded strong derivatives with appropriate alkylene linker lengths (n = 5, 6). The *o*-chlorophenyl derivative **7l** was found to be the most promising compound, such that its inhibitory activities were tens of times stronger than those of phenformin (**1**). Furthermore, biguanide **7l**, at the lowest dose of the series of compounds synthesized here, also markedly reduced the protein expression of HIF-1α induced by hypoxia and the protein expression of GRP78 and GRP94 induced by glucose starvation, in addition to inhibiting angiogenesis. Although these biguanides resulted in potent HIF-1 inhibitory activity, no selective cytotoxicity was observed under hypoxic conditions (1% O_2_). It has been reported that the sensitivity of cancer cells to OXPHOS inhibitors, such as biguanides, is enhanced by low levels of glucose, especially in cells with abnormal mitochondrial function and impaired glucose utilization^44^. Therefore, among the diverse biological activities of biguanides, their modulating effects on energy metabolism may be the main contributor to their selective cytotoxicity under low glucose conditions. Metabolic flux analysis of tumor cells revealed that these newly produced biguanides strongly inhibit OXPHOS and activate compensative glycolysis in the presence of glucose, whereas both are strongly suppressed in the absence of glucose, resulting in cellular energy depletion and apoptosis. Since apoptosis induced by energy stress in combination with metformin and 2-DG is mediated by AMPK^50^, the apoptosis induced by energy depletion in the presence of the biguanides reported herein may also be related to AMPK signaling. We have previously shown that the biguanide derivatives suppress HIF-1 and UPR-dependent transcriptional activation and the expression of c-Myc and ATF4 proteins, while they activate AMPK^24,26^. Biguanides will be utilized effectively through synthetic lethal strategies by exploiting the specific metabolic vulnerability of transformed cells or by inducing changes in the microenvironment with drugs^49,51^. In this paper, we present new potent biguanides that can efficiently inhibit mitochondrial respiration under glucose deprivation conditions in tumor microenvironment, ultimately leading to cell death due to energy depletion. In addition, they can potently suppress HIF-1 and UPR signaling, which are key factors for adaptation to cancer microenvironmental stress. As a result, the new biguanides may have multifunctional properties that disable stress response mechanisms, such as hypoxia, endoplasmic reticulum stress, and metabolic stress, and cause synthetic lethality in combination with potent OXPHOS inhibition.

## Experimental section

### General synthetic procedure

All commercially available reagents and solvents were used without further purification. Normal-phase thin layer chromatography (TLC) was carried out on Silica gel 60 F254 (Merck, 1.05715.0009) using reagent grade solvents. TLC was detected by the absorption of UV light (254 nm) or using a visualization reagent (molybdophosphoric acid). Column chromatography was performed on silica gel (AP-300S Taiko-shoji) or NH silica gel (Chromatorex DM1020, 100–200 mesh FUJI SILYSIA CHEMICAL LTD.) with mixed solvents as described. ^1^H and ^13^C NMR spectra were obtained for samples in the indicated solution at 25 °C utilizing the JEOL JNM-ECA500 spectrometer at 500 MHz frequency for ^1^H or the JNM-AL400 spectrometer at 400 MHz frequency for ^1^H in CD_3_OD or deuterated dimethylsulfoxide (DMSO)-*d*_6_ with tetramethylsilane as an internal standard. ^1^H NMR chemical shifts are reported in terms of the chemical shift (δ, ppm) relative to the singlet corresponding to tetramethylsilane at 0 ppm. Splitting patterns are designated as follows: s, singlet; d, doublet; t, triplet; q, quartet; m, multiplet; br, broad. Coupling constants are reported in Hz. ^13^C NMR spectra were fully decoupled and are reported in terms of the chemical shift (δ, ppm) relative to a septet at δ = 39.5 ppm corresponding to DMSO- *d*_6_ or a septet at δ = 49.0 ppm corresponding to CD_3_OD. Melting point was measured with a MP-J3 apparatus (Yanaco Technical Science). Electrospray ionization-mass (ESI)-mass spectrometry or direct analysis in real time (DART)-mass spectrometry measurements were carried out on the JEOL JMS-T100TD spectrometer, and (FAB)-mass spectrometry measurements were carried out on JEOL JMX-SX 102 sppectrometer. Elementary analysis was performed on a MT-5 CHN corder (Yanaco Technical Science). Microwave reactions were performed with Initiator 2.0 (Biotage AB). Solvents were evaporated under reduced pressure on a rotary evaporator.

### General procedure for the synthesis of biguanides **2**, **3** and **7a**–**7q**^24^

The reaction procedure followed the literature procedure. The amine derivatives (compounds **4**, **5** and **6a–6q**, 0.3–6.23 mmol) was added to a solution of dicyandiamide (1.0 eq.) in 0.8–5.0 mL of CH_3_CN, and then TMSCl (1.1 eq.) was slowly added dropwise to the mixture. With Initiator 2.0, the mixture was stirred and irradiated for 10–30 min at 130 or 150 °C. After the mixture was cooled down to approximately 50 °C, *i*PrOH (3.0 eq.) was added slowly and the mixture was further stirred and irradiated at 125 °C for 1 min. The precipitate was filtrated and washed with CH_3_CN twice to give the biguanide hydrochloride salt.

#### 2-(2-Methylphenyl)ethylbiguanide (**2**)^24^

Following the general procedure for the synthesis of biguanides, the reaction of **4** (540 mg, 4.0 mmol) was performed (130 °C, 10 min, 12.0 mL CH_3_CN) to give **2** hydrochloride (764 mg, 75%). The analytical sample was obtained by recrystallization from EtOH to obtain **2** dihydrochloride as a colorless powder. mp 171.5–172.5 °C (lit. 173–174 °C)^24^.

#### 2-(2-Chlorophenyl)ethylbiguanide (**3**)^24^

Following the general procedure for the synthesis of biguanides, the reaction of **5** (623 mg, 4.0 mmol) was performed (130 °C, 10 min, 12.0 mL CH_3_CN) to give **3** hydrochloride (1.10 g, quant.). The analytical sample was obtained by recrystallization from EtOH to obtain **3** dihydrochloride as a colorless powder. mp 159–160 °C (lit. 159–161 °C)^24^.

#### 2-(2-Bromophenyl)ethylbiguanide (**7a**)

Following the general procedure for the synthesis of biguanides, the reaction of **6a** (200 mg, 1.0 mmol) was performed (130 °C, 10 min, 3.0 mL CH_3_CN) to give **7a** hydrochloride (255.7 mg, 80%). The analytical sample was obtained by recrystallization from EtOH to obtain **7a** dihydrochloride as a colorless powder. mp 173–175 °C; ^1^H NMR (500 MHz, DMSO-*d*_6_): δ = 3.02 (br s, 2H), 3.50 (br s, 2H), 7.21 (t, *J* = 6.9 Hz, 1H), 7.37 (t, *J* = 7.4 Hz, 1H), 7.51 (d, *J* = 6.3 Hz, 1H), 7.62 (d, *J* = 7.4 Hz, 1H), 7.74, 8.58, 9.22, 9.72 (br s, total 6H); ^13^C NMR (125 MHz, DMSO-*d*_6_): δ = 33.5, 42.1, 123.9, 128.0, 128.9, 131.1, 132.6, 137.2, 152.1, 155.1; LRMS (DART+): m/z [M+H]^+^: 284; Anal. Calcd for C_10_H_16_BrCl_2_N_5_: C, 33.64; H, 4.52; N, 19.61. Found: C, 33.61; H, 4.50; N, 19.60.

#### 2-(2-Iodophenyl)ethylbiguanide (**7b**)

Following the general procedure for the synthesis of biguanides, the reaction of **6b** (1.54 g, 6.23 mmol) was performed (130 °C, 10 min, 5.0 mL CH_3_CN) to give **7b** hydrochloride (1.71 g, 66%). The analytical sample was obtained by recrystallization from EtOH to obtain **7b** dihydrochloride as a colorless powder. mp 159 °C; ^1^H NMR (500 MHz, DMSO-*d*_6_): δ = 2.98 (br s, 2H), 3.44 (br s, 2H), 7.02 (t,* J* = 7.5 Hz, 1H), 7.38 (t,* J* = 7.5 Hz, 2H), 7.87 (t,* J* = 7.5 Hz, 1H), 7.46, 8.48, 9.11, 9.64 (br s, total 6H) ; ^13^C NMR (125 MHz, DMSO-*d*_6_): δ = 38.5, 42.9, 101.4, 129.2, 129.4, 130,6, 139.7, 141.0, 152.6, 155.6; HRMS (ESI+): *m/z* calcd for C10H15IN5^+^ [M+H]^+^: 332.0367, found: 332.0339; Anal. Calcd for C_10_H_16_Cl_2_IN_5_: C, 29.72; H, 3.99; N, 17.33. Found: C, 29.74; H, 3.99; N, 17.10.

#### 2-(2-Trifluoromethylphenyl)ethylbiguanide (**7c**)

Following the general procedure for the synthesis of biguanides, the reaction of **6c** (200 mg, 0.95 mmol) was performed (130 °C, 10 min, 3.0 mL CH_3_CN) to give **7c** hydrochloride (262 mg, 80%). The analytical sample was obtained by recrystallization from EtOH to obtain **7c** dihydrochloride as a colorless powder. mp 146–147 °C; ^1^H NMR (500 MHz, CD_3_OD): δ =  3.18 (br s, 2H), 3.60 (br s, 2H), 7.44 (t, *J* = 7.3 Hz, 1H), 7.49–7.78 (m, 4H); ^13^C NMR (100 MHz, CD_3_OD): δ = 31.6, 44.9, 126.1 (q, *J* = 277.3 Hz), 127.2, 127.3, 128.6, 129.7 (q, *J* = 29.6 Hz), 132.8, 133.7, 154.3, 156.7; LRMS (DART+): *m/z* [M+H]^+^: 274; Anal. Calcd for C_11_H_16_F_3_Cl_2_N_5_: C, 38.16; H, 4.66; N, 20.23. Found: C, 37.99; H, 4.53; N, 20.22.

#### 2-(4-Ethylphenyl)ethylbiguanide (**7d**)

Following the general procedure for the synthesis of biguanides, the reaction of **6d** (149 mg, 1.0 mmol) was performed (130 °C, 10 min, 3.0 mL CH_3_CN) to give **7d** hydrochloride (250 mg, 93%). The analytical sample was obtained by recrystallization from EtOH to obtain **7d** dihydrochloride as a colorless powder. mp 178–179 °C; ^1^H NMR (500 MHz, DMSO-*d*_6_): δ = 1.16 (t, *J* = 7.6 Hz, 3H), 2.57 (q, *J* = 7.6 Hz, 2H), 2.84 (br s, 2H), 3.47 (br s, 2H), 7.15 (d, *J* = 7.8 Hz, 2H), 7.23 (d, *J* = 7.8 Hz, 2H), 7.79, 8.55, 9.15, 9.63 (br s, total 6H); ^13^C NMR (125 MHz, DMSO-*d*_6_): δ = 15.7, 27.8, 33.0, 43.8, 127.8, 128.7, 135.3, 141.9, 152.0, 155.1; LRMS (DART+): *m/z* [M+H]^+^: 234; Anal. Calcd for C_12_H_21_Cl_2_N_5_: C, 47.06; H, 6.91; N, 22.87. Found: C, 47.24; H, 6.90; N, 22.61.

#### 2-(4-Benzoylphenyl)ethylbiguanide (**7e**)

Following the general procedure for the synthesis of biguanides, the reaction of **6e** (115 mg, 0.510 mmol) was performed (130 °C, 10 min, 3.0 mL CH_3_CN) to give **7e** hydrochloride (28.4 mg, 14%). The analytical sample was obtained by recrystallization from EtOH to obtain **7e** dihydrochloride as a colorless powder. mp 148 °C; ^1^H NMR (500 MHz, DMSO-*d*_6_): δ = 3.01 (br s, 2H), 3.58 (br s, 2H), 7.44–7.62 (m, 4H), 7.64–7.80 (m, 5H), 8.56, 9.20, 9.67 (br s, total 6H); ^13^C NMR (125 MHz, DMSO-*d*_6_): δ = 33.5, 43.2, 128.6, 129.1, 129.6, 129.9, 132.6, 135.3, 137.2, 143.6, 152.1, 155.1, 195.5; HRMS (ESI+): *m/z* calcd for C_17_H_20_N_5_O^+^ [M+ H]^+^: 310.1668, found: 310.1653; Anal. Calcd for C_17_H_21_Cl_2_N_5_O: C, 53.41; H, 5.54; N, 18.32, found: C, 53.22; H, 5.49; N, 18.59.

#### Phenylbiguanide (**7f**)^29^

Following the general procedure for the synthesis of biguanides, the reaction of aniline (**6f**, 168 mg, 1.8 mmol) was performed (150 °C, 15 min, 2.4 mL CH_3_CN) to give **7f** hydrochloride (320 mg, 83%). The analytical sample was obtained by recrystallization from *i*PrOH to obtain **7f** hydrochloride as a colorless needle. mp 246–249 °C (lit. 244–247 °C); ^1^H NMR (400 MHz, DMSO-*d*_6_): δ = 7.04–7.08 (m, 3H), 7.30–7.35 (m, 8H), 9.79 (s, 1H); LRMS (FAB+): *m/z* [M+H]^+^: 178.

#### Benzylbiguanide (**7g**)

Following the general procedure for the synthesis of biguanides, the reaction of benzylamine (**6 g**, 193 mg, 1.8 mmol) was performed (150 °C, 15 min, 2.4 mL CH_3_CN) to give **7g** hydrochloride (245.1 mg, 60%). The analytical sample was obtained by recrystallization from *i*PrOH to obtain **7g** hydrochloride as a colorless needle. mp 197–199 °C (lit. 196–197 °C)^52^; ^1^H NMR (400 MHz, DMSO-*d*_6_): δ = 4.35 (d,* J* = 5.8 Hz, 2H), 7.09 (s, 4H), 7.22–7.48 (m, 7H), 7.95 (br s, 1H); LRMS (FAB+): *m/z* [M+H]^+^: 192. Anal. Calcd for C_9_H_14_ClN_5_: C, 47.47; H, 6.20; N, 30.76. Found: C, 47.44; H, 6.12; N, 30.69.

#### 3-Phenylpropylbiguanide (**7h**)^29^

Following the general procedure for the synthesis of biguanides, the reaction of 3-phenylpropylamine (**6h**, 243 mg, 1.8 mmol) was performed (150 °C, 15 min, 2.4 mL CH_3_CN) to give **7h** hydrochloride (137 mg, 30%). The analytical sample was obtained by recrystallization from EtOH and *i*PrOH to obtain **7h** dihydrochloride as a colorless solid. mp 197–200 °C; ^1^H NMR (400 MHz, DMSO-*d*_6_): δ = 1.84 (br s, 2H), 2.65 (t,* J* = 7.7 Hz, 2H), 3.24 (br s, 2H) 7.18–7.48 (m, 5H), 8.44, 9.04, 9.51 (br s, total 6H); LHRMS (FAB+): *m/z* [M + H]^+^: 220.

#### 4-Phenylbutylbiguanide (**7i**)

Following the general procedure for the synthesis of biguanides, the reaction of 4-phenylbutylamine (**6i**, 149 mg, 1.0 mmol) was performed (130 °C, 10 min, 3.0 mL CH_3_CN) to give **7i** hydrochloride (264 mg, 98%). The analytical sample was obtained by recrystallization from EtOH to obtain **7i** dihydrochloride as a colorless powder. mp 158–160 °C; ^1^H NMR (400 MHz, CD_3_OD): δ = 1.70 (br, 4H), 2.68 (t, *J* = 7.0 Hz, 2H), 3.33 (t, *J* = 7.0 Hz, 2H), 7.15–7.28 (m, 5H); LRMS (DART+): *m/z* [M+H]^+^: 234; Anal. Calcd for C_12_H_21_Cl_2_N_5_O_2_: C, 47.06; H, 6.91; N, 22.87. Found: C, 47.02; H, 6.86; N, 23.10.

#### 5-Phenylpentylbiguanide (**7j**)

Following the general procedure for the synthesis of biguanides, the reaction of 5-phenylpentylamine (**6j**, 163 mg, 1.0 mmol) was performed (130 °C, 10 min, 3.0 mL CH_3_CN) to give **7j** hydrochloride (209 mg, 74%). The analytical sample was obtained by recrystallization from EtOH to obtain **7j** dihydrochloride as a colorless powder. mp 164–165 °C; ^1^H NMR (500 MHz, DMSO-*d*_6_): δ = 1.31–1.37 (m, 2H), 1.56–1.61 (m, 4H), 2.58 (t, *J* = 7.4 Hz, 2H), 3.23 (q, *J* = 6.3 Hz, 2H), 7.15–7.30 (m, 5H), 7.88, 8.55, 9.09, 9.52 (br s, total 6H); ^13^C NMR (125 MHz, DMSO-*d*_6_): δ = 25.8, 27.2, 30.6, 35.1, 42.3, 125.7, 128.3, 128.3, 142.1, 151.8, 155.0; LRMS (DART+): *m/z* [M+H]^+^: 248; Anal. Calcd for C_13_H_23_Cl_2_N_5_: C, 48.75; H, 7.24; N, 21.87. Found: C, 48.50; H, 7.19; N, 21.93.

#### 6-Phenylhexylbiguanide (**7k**)

Following the general procedure for the synthesis of biguanides, the reaction of 6-phenylhexylamine (**6k**, 177 mg, 1.0 mmol) was performed (130 °C, 10 min, 3.0 mL CH_3_CN) to give **7k** hydrochloride (261 mg, 88%). The analytical sample was obtained by recrystallization from EtOH to obtain **7k** dihydrochloride as a colorless powder. mp 168–169 °C; ^1^H NMR (500 MHz, DMSO-*d*_6_): δ = 1.27–1.37 (m, 4H), 1.52–1.58 (m, 4H), 2.56–2.59 (t, *J* = 7.4 Hz, 2H), 3.23 (q, *J* = 6.5 Hz, 2H), 7.15–7.29 (m, 5H), 7.94, 8.56, 9.11, 9.51 (br s, total 6H); ^13^C NMR (125 MHz, DMSO-*d*_6_): δ = 26.0, 27.4, 28.3, 30.9, 35.1, 42.4, 125.6, 128.2, 128.3, 142.3, 151.8, 155.0; LRMS (DART+): *m/z* [M+H]^+^: 262; Anal. Calcd for C_14_H_25_Cl_2_N_5_: C, 50.30; H, 7.54; N, 20.95. Found: C, 50.15; H, 7.52; N, 21.02.

#### 5-(2-Chlorophenyl)pentylbiguanide (**7l**)

Following the general procedure for the synthesis of biguanides, the reaction of **6l** (198 mg, 1.0 mmol) was performed (130 °C, 10 min, 3.0 mL CH_3_CN) to give **7l** hydrochloride (220 mg, 69%). The analytical sample was obtained by recrystallization from EtOH to obtain **7l** dihydrochloride as a colorless powder. mp 153–155 °C; ^1^H NMR (500 MHz, CD_3_OD): δ = 1.46–1.52 (m, 2H), 1.66–1.77 (m, 4H), 2.78 (t, *J* = 7.7 Hz, 2H), 3.34 (t, *J* = 7.2 Hz, 2H), 7.14–7.34 (m, 4H); ^13^C NMR (125 MHz CD_3_OD): δ = 26.2, 27.4, 29.3, 33.0, 42.9, 126.8, 127.3, 129.1, 130.4, 133.5, 139.7, 152.4, 155.2; LRMS (DART+): *m/z* [M+H]^+^: 282; Anal. Calcd for C_13_H_22_Cl_3_N_5_: C, 44.02; H, 6.25; N, 19.74. Found: C, 43.75; H, 6.19; N, 19.70.

#### 1-Methyl-2-(2-chlorophenyl)ethylbiguanide (**7m**)

Following the general procedure for the synthesis of biguanides, the reaction of **6m** (102 mg, 0.6 mmol) was performed (150 °C, 5 min, 0.8 mL CH_3_CN). Then the mixture was evaporated under reduced pressure to give the crude product, which was purified by NH silica gel column chromatography (CHCl_3_/MeOH = 10 : 1) to obtain **7m** (92.6 mg, 61%) as a colorless amorphous. ^1^H NMR (400 MHz, CD_3_OD): *δ* = 2.96 (s, 3H), 3.04 (t, *J* = 7.5 Hz, 2H), 3.69 (t, *J* = 7.4 Hz, 2H), 7.10–7.40 (m, 4H); ^13^C NMR (100 MHz, CD_3_OD): *δ* = 32.7, 36.5, 51.3, 128.3, 129.4, 130.6, 132.4, 135.1, 137.3, 160.3, 160.5; HRMS(ESI+): *m/z* calcd for C_11_H_17_ClN_5_^+^ [M+ H]^+^: 254.1172, found: 254.1166.

#### 1-[2-(2-Chlorophenyl)ethyl]-1-prop-2-yn-1-ylbiguanide (**7n**)

Following the general procedure for the synthesis of biguanides, the reaction of **6n** (58.1 mg, 0.3 mmol) was performed (150 °C, 10 min, 4 mL CH_3_CN). Then the mixture was evaporated under reduced pressure to give the crude product, which was purified by NH silica gel column chromatography (CHCl_3_/MeOH = 25 : 1) to obtain **7n** (59.1 mg, 71%) as a white paste. ^1^H NMR (500 MHz, CD_3_OD): δ = 1.89 (s, 1H), 3.12 (t, *J* = 4.8, 2H), 3.75 (t, *J* = 5.0, 2H), 4.17 (s, 2H), 7.17–7.31 (m, 2H), 7.32–7.47 (m, 2H); ^13^C NMR (125 MHz, CD_3_OD): δ = 24.2, 32.5, 34.3, 38.7, 45.1, 128.3, 129.6, 130.6, 132.3, 135.1, 137.3, 159.4, 161.4; HRMS (DART+): *m/z* calcd for C_1__3_H_17_ClN_5_^+^ [M + H]^+^ : 278.1173, found: 278.1183.

#### 1-Butyl-[2-(2-chlorophenyl)ethyl]biguanide (**7o**)

Following the general procedure for the synthesis of biguanides, the reaction of **6o** (106 mg, 0.5 mmol) was performed (150 °C, 15 min, 0.8 mL CH_3_CN). Then the mixture was evaporated under reduced pressure to give the crude product, which was purified by NH silica gel column chromatography (Acetone/MeOH = 9:1) to obtain **7o** (20.6 mg, 14%) as a colorless amorphous. ^1^H NMR (500 MHz, CD_3_OD): δ = 0.93 (t, *J* = 7.4 Hz, 3H), 1.21–1.407 (m, 2H), 1.45–1.65 (m, 2H), 3.05 (t, *J* = 7.6 Hz, 2H), 3.14–3.264 (m, 2H), 3.63 (t, *J* = 7.6 Hz, 2H), 7.15–7.30 (m, 2H), 7.39 (d, 2H, *J* = 6.4 Hz),; ^13^C NMR (100 MHz, CD_3_OD): *δ* = 14.0, 20.8, 128.8, 130.1, 130.8, 132.6, 135.0, 136.0, 154.4, 157.2; HRMS(ESI+): *m/z* calcd for C_14_H_23_ClN_5_^+^ [M + H]^+^: 296.1642, found: 296.1615.

#### *N-*(3-{1-[2-(2-Chlorophenyl)ethyl]-3-carbamimidoylguanidino}propyl)pent-4-enamide (**7p**)

Following the general procedure for the synthesis of biguanides, the reaction of **6p** (88.4 mg, 0.3 mmol) was performed (150 °C, 15 min, 4 mL CH_3_CN). Then the mixture was evaporated under reduced pressure to give the crude product, which was purified by NH silica gel column chromatography (CHCl_3_/MeOH = 15 : 1) to obtain **7p** (54.0 mg, 43% ) as a pale yellow paste. ^1^H NMR (500 MHz, CD_3_OD): δ = 1.77 (br s, 2H), 2.23–2.40 (m, 4H), 3.05 (t, *J* = 7.5 Hz, 2H), 3.11–3.25 (m, 2H), 3.26–3.31 (m, 2H), 3.62 (t, *J* = 7.5 Hz, 2 H), 5.00–5.20 (m, 2H), 5.77–5.88 (m, 1H), 7.18–7.45 (m, 4H); ^13^CNMR (125 MHz, CD_3_OD): δ = 28.5, 30.9, 32.7 36.4, 37.7, 115.9, 119.9, 128.4, 129.6, 130.9, 132.5, 135.1, 138.2, 159.9, 163.4, 175.6; HRMS (DART+): *m/z* calcd for C_18_H_28_ClN_6_O^+^ [M + H]^+^: 379.2013, found: 379.2026.

#### 1-[2-(2-Chlorophenyl)ethyl]-5-[2-(2-chlorophenyl)ethyl]biguanide dihydrochloride (**7q**)

Following the general procedure for the synthesis of biguanides, the reaction of **5** (284 mg, 1.8 mmol) was performed (150 °C, 10 min, 3.0 mL CH_3_CN) to give **7q** hydrochloride (269 mg, 71%). The analytical sample was obtained by recrystallization from EtOH to obtain **7q** dihydrochloride as a colorless powder. ^1^H NMR (400 MHz, CD_3_OD): δ = 3.13 (br s, 2H), 3.60 (br s, 2H), 7.28 (br s, 2H), 7.40 (br s, 2H); Anal. Calcd for C_18_H_23_Cl_4_N_5_: C, 47.91; H, 5.14; N, 15.52. Found: C, 47.51; H, 5.15; N, 15.65.

## Biological evaluation

### Materials and compounds

Eagle’s minimum essential medium (E-MEM, 051-07615), 1 mmol/L sodium pyruvate, RPMI-1640 (glucose, 2000 mg/L, 189-02025), Sodium Dodecyl Sulfate (SDS, 194-13985), Tween 20, bovine serum albumin (5217/100G) and 45w/v% *D*(+)-Glucose Solution (079-05511) were obtained from FUJIFILM Wako Pure Chemical Corporation (Osaka, Japan). MEM non-essential amino acid (11140050), RPMI 1640 (no glucose, 11879020) and were obtained from Thermo Fisher Scientific, Inc, (GIBCO, Tokyo, Japan). 50 units/mL penicillin, 50 μg/mL streptomycin and 50 μg/mL kanamycin were obtained from Meiji Seika Pharma Corp, Ltd, (Tokyo, Japan). G418, thiazolyl blue tetrazolium bromide (MTT), Anti-Mouse IgG (whole molecule) peroxidase conjugate (A4416) and Anti-Goat IgG (whole molecule) peroxidase conjugate (A5420) were obtained from Sigma-Aldrich (Tokyo, Japan). XF 200 mM Glutamine solution (103578-100), XF DMEM medium pH 7.4 (103575-100), XF 100 mM pyruvate solution (103578-100), oligomycin, carbonyl cyanide-4-(trifluoromethoxy)phenylhydrazone (FCCP), rotenone, antimycin A, oligomycin and 2-deoxy-*D*-glucose were obtained from Agilent Technologies (Tokyo, Japan). Annexin V-FITC Apoptosis Detection kit (PK-CA577-K101-100) were obtained from Clontech TaKaRa cellartis.

### Preparation of test compounds

All of the test compounds were prepared as stock solutions of 100 mM in DMSO and stored in aliquots at − 20 °C. The final concentration of DMSO is less than 1% (v/v) for all in vitro assays**.**

### Cell lines and culture conditions

Cells were maintained in EMEM supplemented with 1% (v/v) MEM non-essential amino acid (100×) and 1 mmol/L sodium pyruvate for Human Embryo Kidney HEK293(JCRB Cell Bank, JCRB9068) and U87MG glioblastoma(ATCC, HTB-14), or RPMI-1640 for human colon cancer HT-29(ATCC, HTB-38) and A549 lung carcinoma(JCRB Cell Bank, JCRB0076). Both mediums were supplemented with 10% heat-inactivated fetal bovine serum, 50 units/mL penicillin, 50 μg/mL streptomycin, 50 μg/mL kanamycin (Meiji Seika Pharma Corp, Ltd). All cell lines were cultured at 37 °C in a humidified atmosphere containing 5% CO_2_ as the normal growth condition. Cells were treated with 0.3 mM 2-DG in normal medium (2000 mg/L glucose) or cultured in glucose-free medium for glucose deprivation treatment. Glucose-free RPMI 1640 medium was supplemented with 10% heat-inactivated fetal bovine serum. For hypoxia conditions, an air-tight chamber (Modular Incubator Chamber from Billups-Rothenberg Inc.) filled with mixed gas (1% O_2_, 94% N_2_, and 5% CO_2_). All compounds were added at various final concentrations immediately after replacing the medium with 2-DG-containing medium or glucose-free medium, whereas hypoxic treatment was performed one hour after the addition of compounds.

### Luciferase reporter assay

The luciferase reporter assay was carried out according to the procedure reported previously^30^. We used previously established stable transfectants, HEK293 p2.1 #3 for the HIF-1-dependent luciferase assay and HEK293 GRP78 #85 for UPR-dependent luciferase assay^24^. The HEK293 clone cells were plated into a 24-well plate (8.0 × 10^4^ cells/well) and incubated for 24 h. For hypoxia treatment, the cells were incubated for 1 h after the medium was replaced with fresh medium containing 0.25% DMSO supplemented with the indicated concentrations of the test compounds, and then incubated under normoxic or hypoxic conditions (1% O_2_) for another 24 h. In the case of glucose deprivation treatment, cells were incubated for 24 h after replacement with fresh medium containing the indicated concentrations of the test compounds and 0.3 mM 2-DG. The luciferase assays were performed according to the protocol of Luciferase Assay Kit (Roche Diagnostics, Mannheim, Germany). Luciferase activity was measured using a FB12 luminometer (Titertek-Berthold, Bad Wildbad, Germany). The luciferase activities were normalized with respect to protein concentration.

### Cell viability assay

HT29 cells were seeded in 96-well (3.0 × 10^3^ cells per well) and cultured overnight. Then treated with various concentrations of compounds in the normal or glucose-free medium for 48 h. Then the medium was replaced with fresh growth medium, and cells were cultured for further 16 h. Subsequently, 10 μL of thiazolyl blue tetrazolium bromide (Sigma-Aldrich, St Louis, MO, USA) solution (0.5 mg/mL) was added to each well. After 4 h incubation at 37 °C, the medium was removed, 100 μL of DMSO was added then absorbance of each well was measured at 570 nm by MULTISKAN JX plate reader. Relative cell survival (mean ± SD of triplicate determinations) was calculated by setting each of the control absorbance from non-drug treated cells as 100%.

### Rm value

Rm values of the tested compounds were measured by TLC (60 RP-8 F254S, MERCK) analysis eluted with a mixture of MeOH and 20 mM sodium phosphate buffer (pH 7.2) (8 : 2). Then Rm values were calculated as follows: Rm = log(1/Rf − 1).

### Immunoblot analysis

HT29 cells were cultured overnight in Φ100 mm culture dishes (6.0 × 10^6^ cells) and then treated with various concentrations of compounds under hypoxia for 4 h or glucose deprived condition for 24 h. Then, cells were lysed, and the protein concentrations were determined using a Pierce BCA Protein Assay Kit (Thermo Scientific). After electrophoresis of the protein samples with a 7% sodium dodecyl sulfate (SDS)-polyacrylamide gel, the proteins were electrotransferred to Hybond-ECL nitrocellulose membranes (GE Healthcare, Little Chalfont, UK) or polyvinylidene fluoride (PVDF) membrane (Bio-Rad Laboratories, Inc., Hercules, CA, USA). Immunoblot analysis was probed with the following primary antibodies: mouse KDEL mono- clonal antibody (10C3) for detection of GRP78 and GRP94 (Stressgen SPA-827, Enzo Life Sciences, Inc., Farmingdale, NY, USA), mouse monoclonal antibody against human HIF-1α (H1alpha67) (NB100-105, Novus Biologicals, Littleton, CO, USA), goat polyclonal anti-human β-actin antibody (I-19) (sc-1616, Santa Cruz Biotechnology, Inc., Dallas, TX, USA). Membranes were pretreated for 1 h with Tris-buffered saline with Tween 20 (Wako Pure Chemical Industries, Ltd) (TBS–T) [50 mM Tris–HCl (pH 7.5), 150 mM NaCl, and 0.1% Tween 20] containing 5% nonfat dry milk or 1% bovine serum albumin (BSA, Wako Pure Chemical Industries, Ltd) at room temperature. Then the membranes were incubated, at 4 °C for 10 h with the anti-HIF-1α antibody (diluted 1:1,000), at room temperature for 1 h with anti-KDEL antibody (diluted 1:1,000), or at room temperature for 1 h with the anti-β-actin antibody (diluted 1:3,000). The membranes were washed with TBS–T containing 5% nonfat dry milk or 1% BSA at room temperature, and then incubated at room temperature for 1 h with appropriate horseradish peroxidase-labeled secondary: Anti-Mouse IgG (whole molecule) peroxidase conjugate (A4416; Sigma-Aldrich) and Anti-Goat IgG (whole molecule) peroxidase conjugate (A5420, Sigma-Aldrich). After washing with TBS–T, the specific signals were detected with an enhanced chemiluminescence detection system (Pierce Western Blotting Substrate, Thermo Scientific) or chemiluminescence detection system (Immobilon Western Chemiluminescent HRP substrate, Merck Millipore, Billerica, MA, USA) and visualized with a Fujifilm Luminescent Image Analyzer LAS-3000 (Fujifilm, Tokyo, Japan).

### OCR and ECAR measurements for the analysis of cell energy phenotypes

Cellular OCR and ECAR of live cells were measured using a Seahorse XFp Extracellular Flux Analyzer (Agilent, Santa Clara, CA, USA). The cellular mitochondrial function and glycolytic rate were analyzed via the XF Cell Mito Stress Test and XF Glycolysis Stress Test kit (Agilent), respectively according to the manufacturer's protocol. In brief, HT29, A549 or U87MG cells (2.5 × 10^4^ cells/well) were seeded into an XFp cell culture microplate and incubated for 24 h. The sensor cartridges of the XFp analyzer were hydrated in a 37 °C non-CO_2_ incubator the day before the experiment. To examine the mitochondrial function, injection port A on the sensor cartridge was loaded with 1.5 μM oligomycin (complex V inhibitor), port B was loaded with 2 μM carbonyl cyanide-4-(trifluoromethoxy)phenylhydrazone (FCCP), and port C with 0.5 μM rotenone/antimycin A (inhibitors of complex I and complex III). During sensor calibration, cells were treated with 5–50 μM of test compounds and incubated in a 37 °C non-CO_2_ incubator together with 180 μM of assay medium (XF base medium with 10 mM glucose, 1 mM pyruvate, and 2 mM l-glutamine, pH 7.4) for 1 h. The plate was immediately placed into the calibrated XFp Extracellular Flux Analyzer. For the glycolysis stress test, injection port A on the sensor cartridge was loaded with 10 mM glucose, port B was loaded with 1 μM Oligomycin, and port C with 50 mM 2-deoxy-*D*-glucose. During the sensor calibration, cells were treated with 5–50 μM of test compounds and incubated in a 37 °C non-CO_2_ incubator together with 180 μL of assay medium (XF base medium with 2 mM l-glutamine, pH 7.4) for 1 h. The plate was immediately placed into the calibrated XFp Extracellular Flux Analyzer. The each average of the OCR values measured at three points during the first 15 min in the profile of mitochondrial function and the ECAR values measured simultaneously were used as the respiratory inhibitory activity of biguanides in the presence of glucose (Glc +). Similarly, the mean values of ECAR and OCR determined from the glycolytic functional profile were used as respiratory inhibitory activity under glucose-depleted conditions (Glc −). Then, the OXPHOS and ECAR were measured after treatment of HT29 with biguanides **1, 2, 3** and **7l** under 10 mM glucose (Glc +) and glucose deprivation conditions (Glc −) in a non-CO_2_ incubator. DMEM-based medium supplemented with 10 mM glucose, 1 mM sodium pyruvate, and 2 mM l-glutamine at final concentrations was prepared for the normal glucose assay medium, and 2 mM l-glutamine supplemented medium was used for the glucose-free assay condition.

### CAM assay in fertilized chicken eggs

The antiangiogenic effect of compounds in vivo was evaluated by CAM assay as reported procedure^42^. Briefly, fertilized chicken eggs were incubated at 37.5 °C in a humidified incubator with forced air circulation. Ovalbumin (3 mL) was removed from 3-day-old embryonated eggs. Then a small hole was drilled on each shell of the egg and capped, and the eggs were incubated at 39.0–39.5 °C. After a 1-day incubation, each compound saline solution, with 2.0% DMSO and 1.0% methylcellulose, was applied on the center of silicon rings (outer diameter, 5 mm; inner diameter, 3 mm; height, 1 mm) that were placed on each of the CAMs, and the eggs were incubated at 39.0–39.5 °C for 2 days. A white 20% intralipos solution (Otsuka Pharma-ceutical, Japan) was injected beneath the CAM to enhance visibility of the overlying superficial CAM vessels. The images were captured with a digital camera and scored on the seventh embryonic day. Saline solution, with 2.0% DMSO and 1.0% methylcellulose, was used as a vehicle. Eight to ten eggs were used in total for each data point. The inhibition point was scored by an estimation of the area of the avascular zone. The inhibition ratios were calculated from the following formula: Inhibition ratio (%) = [1 − (point for control CAM/point for drug treated CAM)] × 100.

### FACS analysis of cell death

Apoptosis and cell death were analyzed using the Annexin V-FITC Detection kit according to its instruction manual. Briefly, HT29 cells were seeded at 5 × 10^5^ cells/100-mm dish. After exposure to **2**, **3** or **7l** at concentrations of 10 or 5 μM in normal or glucose-free medium for 48 h, the cells were washed twice with cold PBS, and suspended in a 1× binding buffer at a concentration of 5 × 10^5^ cells/mL, after which Annexin V-FITC and propidium iodide were added. Following incubation for 5 min at room temperature in the dark, 1× binding buffer (500 μL) was added to each tube. The cells were analyzed using a BD FACSVerse flow cytometer and BD FACSuite v1.0.5.3841 software (BD Biosciences) and the fraction of the cell population in different quadrants was determined using quadrant statistics.

### Statistical analysis

For the results of OCR and ECAR measurements, statistical differences between the three groups of samples treated with three compounds were evaluated by Student’s *t*-test as indicated in each figures (*p < 0.05; **p < 0.005; ***p < 0.0005). Dose-response results of the reporter assays and cell viability assays are presented as the mean ± SD of three independent experiments.

## Supplementary Information

Supplementary Informations.
