# Supplementary material for: Development of antitumor biguanides targeting energy metabolism and stress responses in the tumor microenvironment
Source: Sci Rep. 2021 Mar 1;11:4852. doi: 10.1038/s41598-021-83708-w (PMC7921556; doi:10.1038/s41598-021-83708-w)
Supplement: Supplementary file 1 — Supplementary Informations. [file 41598_2021_83708_MOESM1_ESM.pdf]

Supplementary Information

Development of antitumor biguanides targeting energy metabolism and stress responses in the tumor microenvironment

*Takayuki Sakai[1], Yoshiyuki Matsuo[2], Kensuke Okuda[3], Kiichi Hirota[2], Mieko Tsuji[1], Tasuku Hirayama[1], Hideko Nagasawa[1]**

[1] Laboratory of Pharmaceutical and Medicinal Chemistry, Gifu Pharmaceutical University, Gifu-city, Gifu 501-1196, Japan.

[2] Department of Human Stress Response Science Institute of Biomedical Science Kansai Medical University 2-5-1 Shin-machi.

[3] Laboratory of Bioorganic & Natural Products Chemistry, Kobe Pharmaceutical University, 4-19-1 Motoyama-kita, Higashinada, Kobe 658-8558 Japan.

Contents

Chemistry, General synthetic procedure ---**S2**

Syntheses of intermediate compounds ---**S3−S14**

Biological evaluation, Materials, and compounds. --**S15**

Figure S2. ---**S16**

Figure S3. ---**S17**

Figure S4. ---**S18**

Figure S5. ---**S19**

Figure S6. ---**S20**

Figure S7. ---**S21**

References. --**S22**

^{1}H and ^{13}C NMR spectra of new biguanides ---**S23−S46**

Experimental section

Chemistry

General synthetic procedure

All commercially available reagents and solvents were used without further purification. Normal-phase thin layer chromatography (TLC) was carried out on Silica gel 60 F254 (Merck, 1.05715.0009) using reagent grade solvents. TLC was detected under UV light (254 nm) or using a visualization reagent (phosphomolybdic acid). Column chromatography was performed on silica gel (AP-300S Taiko-shoji) or NH silica gel (Chromatorex DM1020, 100–200 mesh FUJI SILYSIA CHEMICAL LTD.) with mixed solvents as described. ^{1}H and ^{13}C NMR spectra were obtained for samples in the indicated solvent at 25 °C utilizing the JEOL JNM-ECA500 spectrometer at 500 MHz frequency for ^{1}H or the JNM-AL400 spectrometer at 400 MHz frequency for ^{1}H in $CDCl_3$, CD_3OD or deuterated dimethylsulfoxide (DMSO)-d_6 with tetramethylsilane as an internal standard. ^{1}H NMR chemical shifts are reported in terms of the chemical shift (δ, ppm) relative to the singlet corresponding to tetramethylsilane at 0 ppm. Splitting patterns are designated as follows: s, singlet; d, doublet; t, triplet; dd, doublet of doublets; td, triplet of doublets; q, quartet; m, multiplet; br, broad. Coupling constants are reported in Hz. ^{13}C NMR spectra were fully decoupled and are reported in terms of the chemical shift (δ, ppm) relative to a triplet at δ = 77.0 ppm corresponding to $CDCl_3$, a septet at δ = 39.5 ppm corresponding to DMSO- d_6 or a septet at δ = 49.0 ppm corresponding to CD_3OD. Melting point was measured with a MP-J3 apparatus (Yanaco Technical Science). Electrospray ionization (ESI)-mass spectrometry or direct analysis in real time (DART)-mass spectrometry measurements were carried out on the JEOL JMS-T100TD spectrometer. Elementary analysis was performed on a MT-5 CHN corder (Yanaco Technical Science). Microwave reactions were performed with Initiator 2.0 (Biotage AB). Solvents were evaporated under reduced pressure on a rotary evaporator.

Syntheses of intermediate compounds

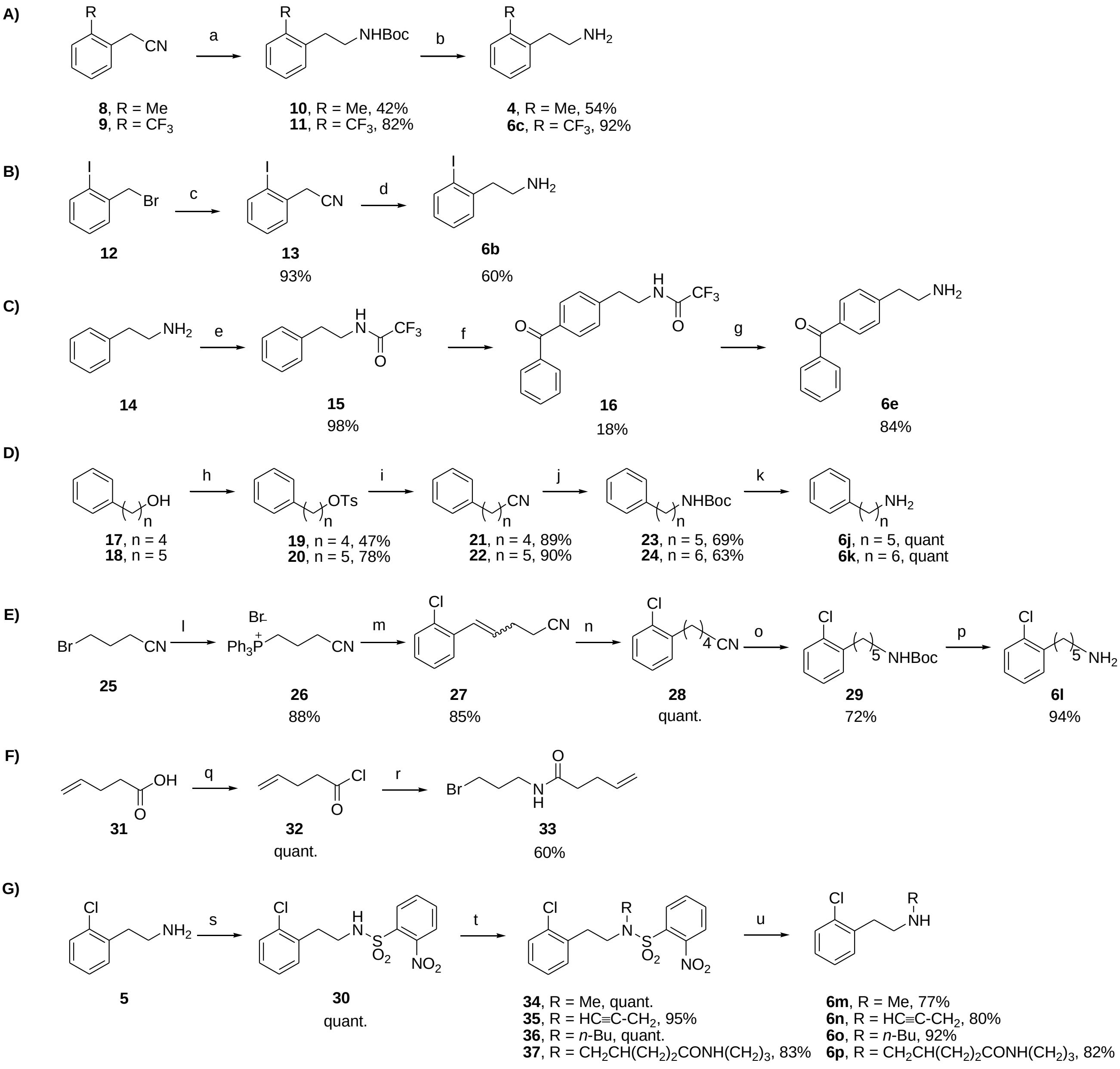

Figure S1. Synthesis of intermediate amines **4**, **6b**, **6c**, **6e**, **6j–p**, **33**

Reagent and conditions: A) (a) (i) $(Boc)_2O$, $NiCl_2$, $NaBH_4$, dry MeOH, 0 °C to rt; (ii) diethylenetriamine, rt; (b) (i) 4M HCl/EtOAc, EtOAc, rt; (ii) NH silica gel column chromatography. B) (c) KCN, EtOH, reflux; (d) 0.93 M BH_3·THF, dry THF, 0 °C to reflux. C) (e) trifluoromethanesulfonic anhydride dry THF, under N_2, rt; (f) benzoic anhydride, TfOH, under N_2, rt; (g) 1 M NaOH aq., under N_2, 0 °C to rt. D) (h) TsCl, pyridine, dry CH_2Cl_2, 0 °C to rt; (i) KCN, DMSO, 120 °C; (j) (i) $(Boc)_2O$, $NiCl_2$, $NaBH_4$, dry MeOH, 0 °C to rt; (ii) diethylenetriamine, rt; (k) (i) 4 M HCl/EtOAc, EtOAc, rt; (ii) NH silica gel column chromatography. E) (l) PPh_3, toluene, reflux, under N_2; (m) NaH, 2-chlorobenzaldehyde, dry THF, dry DMSO, under N_2 at 0 °C to rt, (E form : Z form = 1 : 4); (n) Pd/C, EtOAc, 1,4-

dioxane, under H_2; (o) (i) $(Boc)_2O$, $NiCl_2·6H_2O$, $NaBH_4$, dry MeOH, 0 °C to rt; (ii) diethylenetriamine, rt; (p) (i) 4 M HCl/EtOAc, EtOAc, rt; (ii) NH silica gel column chromatography. F) (q) thionyl chloride, under N_2, 55 to 80 mmHg, rt to 80 °C; (r) 3-bromopropylamine, dry Et_3N, dry CH_2Cl_2, 0 °C. G) (s) 2-nitrobenzenesulfonyl chloride, Et_3N dry CH_2Cl_2, under N_2, rt; (t) RBr, RI, or 80 °C, K_2CO_3, dry DMF, 60 °C; (u) PhSH, KOH, CH_3CN, 50 °C.

General procedure for the preparation of *N*-Boc protected amines 10, 11, 23, 24 and 29.

To a stirred solution of nitrile derivative (**8**, **9**, **21**, **22** and **28**, 3.0–8.0 mmol) in dry methanol (30–200 mL) at 0 °C, Boc_2O (2.0 eq.), and $NiCl_2·6H_2O$ (5–10 mol%) were added. Then $NaBH_4$ (7–10 eq.) was added in small portions over 30 min. The reaction was exothermic and effervescent. The resulting reaction mixture containing a finely divided black precipitate was allowed to warm to room temperature and stirred for total 4–25 h. Then diethylenetriamine (1.0 eq.) was added, and the mixture was stirred for another 30 min before solvent evaporation. After elimination of volatiles under reduced pressure, the purple residue was dissolved in ethyl acetate (100 mL) and washed with saturated $NaHCO_3$ aq (3 × 30 mL). The organic extracts were dried over anhydrous $MgSO_4$, filtered, and evaporated to give the crude product mixture which was purified by silica gel column chromatography to give the target carbamates.

General procedure for the preparation of amines 4, 6c and 6j–6l.

Compound **10, 11, 23, 24** and **29** (1.2–73.0 mmol) was stirred in 4 M HCl/EtOAc (4–50 mL) at room temperature for 6–7 h. The solvent was removed *in vacuo* to obtain amine hydrochlorides as a white powder. Then it was subjected to short NH silica gel column chromatography ($CHCl_3$/methanol = 1:1) to obtain the target amines.

General procedure for the preparation of *N*-alkyl-*N*-nosylamines 34–37.

K_2CO_3 (6.6 mmol) was added to a solution of **30** (3.0 mmol) in anhydrous DMF (5–7 mL) under N_2 at room temperature. Then iodomethane, 1-bromobutane, propargyl bromide or compound **33** (3.3–60 mmol) were added over a period of 10 min at room temperature, and the resulting mixture was stirred at 60 °C under N_2 for 10–42 h. The reaction mixture was allowed to cool to room temperature, diluted with water (25 mL), and extracted with diethyl ether (40 mL × 4). The combined organic extracts were washed with brine (30 mL), dried over anhydrous $MgSO_4$, filtered and evaporated to give the crude mixture which was purified by silica gel column chromatography.

General procedure for the preparation of secondary amines 6m–6p.

10.9 M KOH aq. (0.57–1.38 mL) was added to the solution of thiophenol (6.2–15 mmol) in CH_3CN (2–10 mL) over a period of 10 min at 0 °C. After 5 min, the mixture was warmed to room temperature, and the solution of **34–37** (2.5–6.0 mmol) in CH_3CN (1–2 mL) was added over a period of 20 min at room temperature. The resulting mixture was stirred at 50 °C under N_2 for 50 min. Then the reaction mixture was cooled to room temperature, diluted with water (20 mL) and extracted with CH_2Cl_2 (40 mL × 3). The combined organic extracts were washed with brine (50 mL), dried over anhydrous $MgSO_4$, filtered and evaporated to give the crude mixture which was purified by silica gel column chromatography.

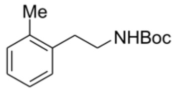

1-*tert*-Butoxycarbonylamino-2-(2-methyl)phenylethane (**10**) [1].

Following the general procedure for reduction and Boc protection of nitrile derivatives, the reaction of compound **8** (1.05 g, 8.00 mmol) was performed. The resulting crude mixture was purified by silica gel column chromatography ($CHCl_3$/*n*-hexane = 1:1) to afford **10** (784.1 mg, 42%) as a yellow oil; ^{1}H NMR (400 MHz, $CDCl_3$): δ = 1.45 (s, 9H), 2.34 (s, 3H), 2.81 (t, *J* = 7.0 Hz, 2H), 3.34 (q, *J* = 7.0 Hz, 2H), 4.58 (s, 1H), 7.13–7.26 (m, 4H).

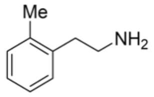

2-Methylphenetylamine (**4**). CAS 55755-16-3

Following the general procedure, the reaction of compound **10** (17.1 g, 73 mmol) was performed to give **4** hydrochloride. Then NH silica gel column chromatography of **4** hydrochloride gave **4** (5.29 g, 54%) as a pale yellow oil.

^{1}H NMR (500 MHz, $CDCl_3$): δ = 2.32 (s, 3H), 2.97 (t, *J* = 7.0 Hz, 2H), 3.09 (t, *J* = 7.0 Hz, 2H), 7.05–7.20 (m, 4H).

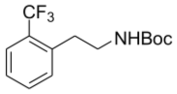

1-*tert*-Butoxycarbonylamino-2-(2-trifuluoromethyl)-phenyl ethane (11).

Following the general procedure for reduction and Boc protection of nitrile derivatives, the reaction of 2-trifuluoromethylbenzyl nitirile (**9**, 741 mg, 4.0 mmol) was performed. The resulting crude mixture was purified by silica gel column chromatography ($CHCl_3$/*n*-hexane = 1:1) to afford **11** (948 mg, 82%) as a pale yellow solid.

Mp 60–61 °C.; ^{1}H NMR (400 MHz, $CDCl_3$): δ = 1.44 (s, 9H), 2.99 (t, *J* = 7.0 Hz, 2H), 3.38 (q, *J* = 7.0 Hz, 2H), 4.63 (s, 1H), 7.31–7.38 (m, 2H), 7.31–7.65 (m, 4H); ^{13}C NMR (125 MHz, $CDCl_3$): δ = 28.4, 33.1, 41.6, 79.3, 124.5 (q, *J* = 273.9 Hz), 126.1 (q, *J* = 5.6 Hz), 126.5, 128.9 (q, *J* = 29.6 Hz), 131.6, 131.8, 137.6, 155.8; LRMS (DART+): *m/z* $[2M+H]^+$: 579; Anal. Calcd. for $C_{14}H_{18}F_3NO_2$: C, 58.12; H, 6.27; N, 4.84, found: C, 58.12; H, 6.21; N, 4.72.

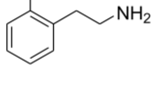

2-Trifuluoromethylphenetylamine (6c) [2]

Following the general procedure, the reaction of compound **11** (902 mg, 3.1 mmol) was performed to give **6c** hydrochloride (705 mg, quant.). Then NH silica gel column chromatography of **6c** hydrochloride (461 mg, 2.0 mmol) gave **6c** (353 mg, 92%) as a pale yellow oil. ^{1}H NMR (500 MHz, $CDCl_3$): δ = 2.94 (br, 2H), 2.98 (br, 2H), 7.31 (t, *J* = 7.7 Hz, 1H), 7.35 (t, *J* = 7.4 Hz, 1H), 7.48 (t, *J* = 7.4 Hz, 1H), 7.64 (t, *J* = 8.0 Hz, 1H).

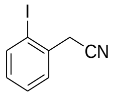

2-Iodobenzyl cyanide (**13**). CAS 40400-15-5

To a stirred solution of 2-Iodobenzyl bromide (**12**, 8.90 g, 30 mmol) in EtOH (50 mL) was added NaCN (4.41 g, 90 mmol). The reaction mixture was refluxed and stirred for 7 h. Then H_2O (50 mL) was added to the mixture and extracted with EtOAc (4 × 50 mL), dried over anhydrous $MgSO_4$. After filtration, the solvent was evaporated under reduced pressure to give the residue, which was purified by silica gel column chromatography (*n*-hexane/EtOAc = 5 : 1) to give **13** (6.77 g, 93%) as a brown oil. LRMS (DART+): *m/z* $[M+H]^+$: 244.

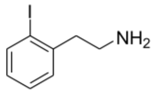

2-Iodophenetylamine (**6b**). CAS 66384-49-4

To a stirred solution of 2-Iodobenzyl cyanide (**13**, 6.76 g, 27.8 mmol) in dry THF (10 mL) was added dropwise 0.93 M BH_3· THF (60 mL, 55.8 mmol) under N_2 at 0 °C. The mixture was stirred at room temperature for 4 h. The solvent was evaporated under reduced pressure to give the residue, which was stirred in 4 M HCl/EtOAc (4 mL) at room temperature for 6 h. The solvent was removed *in vacuo* to obtain **6b** hydrochlorides as a white powder. Then it was subjected to short NH silica gel column chromatography ($CHCl_3$/methanol = 1:1) to obtain **6b** (4.13 g, 60%) as a pale yellow oil. ^1H NMR (500 MHz, $CDCl_3$): δ = 1.44 (s, 9H), 2.90 (t, *J* = 7.0 Hz, 2H), 2.96 (t, *J* = 7.0 Hz, 2H), 6.91 (td, *J* = 2.0 Hz , 7.5 Hz, 1H), 7.19–7.31 (m, 2H), 7.79–7.85 (m, 1H); LRMS (DART+): *m/z* $[M+H]^+$: 248.

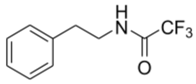

2,2,2-Trifluoro-*N*-(2-phenylethyl)acetamide (**15**) [3] .

To a stirred solution of 2-phenylethanamine (**14**, 606 mg, 5.0 mmol) in dry THF (10 mL) was added dropwise trifluoromethanesulfonic anhydride (1.19 mL, 10.0 mmol) under N_2 at room temperature. The mixture was stirred at the same temperature for 3 h. The resulting mixture was evaporated to give the crude product, which was purified by silica gel chromatography (*n*-hexane/EtOAc = 1 : 1) to give **15** (1.06 g, 98%) as a white solid. ^1H NMR (500 MHz, $CDCl_3$): δ = 2.89 (t, *J* = 7.0 Hz, 2H), 3.64 (q, *J* = 7.0 Hz, 2H), 7.20–7.40 (m, 5H).

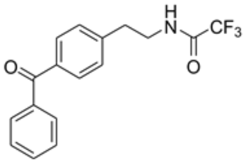

***N*-[2-(4-Benzoylphenyl)ethyl]-2,2,2-trifluoroacetamide** (**16**) [3] .

TfOH (1 mL) was added to compound **15** (740 mg, 3.4 mmol) and benzoic anhydride (1.54 g, 6.8 mmol) at 0 °C. The mixture was stirred under N_2 at room temperature for 4 h. The reaction mixture was diluted with water (20 mL)

and extracted with EtOAc (40 mL × 3). The combined extracts were washed with brine (20 mL), dried over anhydrous $MgSO_4$, filtered, and evaporated under reduced pressure to give the residue, which was purified by silica gel column chromatography (*n*-hexane/EtOAc = 8 : 1) to give **16** (196 mg, 18%) as a colorless oil. ^{1}H NMR (500 MHz, $CDCl_3$): δ = 3.00 (t, *J* = 7.0 Hz, 2H), 3.68 (q, *J* = 7.0 Hz, 2H), 7.17–7.75 (m, 9H).

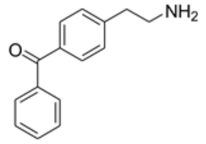

4-Benzophenylethylamine (**6e**) [3].

To a stirred solution of 2-phenylethanamine (**16**, 196 mg, 0.61 mmol) in MeOH (1 mL) was added dropwise 1 M NaOH (4 mL) under N_2 at 0 °C for 2 h. The reaction mixture was warmed to room temperature, diluted with water (20 mL) and extracted with EtOAc (40 mL × 3). The combined extracts were washed with brine (30 mL× 2), dried over anhydrous $MgSO_4$, filtered and evaporated under reduced pressure to give **6e** (115 mg, 84%) as a pale yellow oil. ^{1}H NMR (500 MHz, $CDCl_3$): δ = 2.86 (t, *J* = 7.0 Hz, 2H), 3.04 (q, *J* = 7.0 Hz, 2H), 7.20–7.81 (m, 9H).

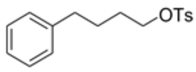

4-Phenylbutyl tosylate (**19**) [4].

To a stirred solution of tosyl chrolide (1.14 g, 6.0 mmol) in CH_2Cl_2 (6 mL) at 0 °C, 4-phenylbutyl alchohol (**17**, 750.1 mg, 5.0 mmol), and pyridine (403 μL, 5.0 mmol) were added. The reaction mixture was allowed to warm to room temperature and stirred for 24 h. Then the mixture was washed with 0.1 M HCl aq. (2 × 15 mL), saturated $NaHCO_3$ aq. (2 × 15 mL), brine (15 mL), and dried over anhydrous $MgSO_4$. After filtration, the volatiles are evaporated under reduced pressure to give the residue, which was purified by silica gel column chromatography (*n*-hexane/$CHCl_3$ = 2:1) to afford **19** (707 mg, 47%) as a pale yellow oil. ^{1}H NMR (400 MHz, $CDCl_3$): δ = 1.66 (br, 4H), 2.44 (s, 3H), 2.56 (t, *J* = 7.0 Hz, 2H), 4.03 (t, *J* = 6.0 Hz, 2H), 7.10 (d, *J* = 6.8 Hz, 2H), 7.18 (t, *J* = 7.5 Hz, 1H), 7.26 (t, *J* = 7.2 Hz, 2H), 7.33 (d, *J* = 8.2 Hz, 2H), 7.78 (d, *J* = 8.2 Hz, 2H).

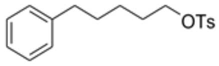

5-Phenylpentyl tosylate (**20**) [5] .

To a stirred solution of tosyl chrolide (1.14 g, 6.0 mmol) in CH_2Cl_2 (6 mL) at 0 °C, 5-phenylpentyl alchohol (**18**, 821.3 mg, 5.0 mmol), and pyridine (403 μL, 5.0 mmol) were added. The reaction mixture was allowed to warm to room temperature and stirred for 48 h. Then the mixture was washed with 0.1 M HCl aq. (2 × 25 mL), saturated $NaHCO_3$ aq. (2 × 25 mL), brine (25 mL), and dried over anhydrous $MgSO_4$. After filtration, the volatiles are

evaporated under reduced pressure to give the residue, which was purified by silica gel column chromatography (*n*-hexane/$CHCl_3$ = 2:1) to afford **20** (1.43 g, 78%) as a pale yellow oil. ^1H NMR (400 MHz, $CDCl_3$): δ = 1.31–1.38 (m, 2H), 1.52–1.60 (m, 2H), 1.63–1.70 (m, 2H), 2.45 (s, 3H), 2.56 (t, *J* = 7.5 Hz, 2H), 4.01 (t, *J* = 6.5 Hz, 2H), 7.12 (d, *J* = 6.8 Hz, 2H), 7.18 (t, *J* = 7.2 Hz, 1H), 7.27 (t, *J* = 7.2 Hz, 2H), 7.34 (d, *J* = 7.7 Hz, 2H), 7.78 (d, *J* = 8.2 Hz, 2H).

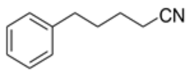

5-Phenylpentylnitrile (**21**) [6].

To a stirred solution of compound **19** (625 mg, 2.1 mmol) in DMSO (5 mL) was added KCN (668 mg, 10.3 mmol). The reaction mixture was allowed to 120 °C and stirred for 5 h. Then H_2O (50 mL) was added to the reaction mixture. The resulting mixture was extracted with EtOAc (4 × 50 mL), dried over anhydrous $MgSO_4$. After filtration, the volatiles are evaporated under reduced pressure to give the residue, which was purified by silica gel column chromatography (*n*-hexane/$CHCl_3$ = 2:1) to afford **21** (291.3 mg, 89%) as a colorless oil. ^1H NMR (400 MHz, $CDCl_3$): δ = 1.67–1.72 (m, 2H), 1.76–1.82 (m, 2H), 2.35 (t, *J* = 7.0 Hz, 2H), 2.66 (t, *J* = 7.2 Hz, 2H), 7.17–7.31 (m, 5H).

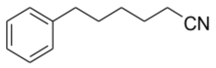

6-Phenylhexylnitrile (**22**) [7].

To a stirred solution of compound **20** (1.91 g, 6.0 mmol) in DMSO (15 mL) was added KCN (1.95 g, 30 mmol). The reaction mixture was allowed to 120 °C and stirred for 5 h. Then H_2O (50 mL) was added to the mixture. The resulting mixture was extracted with EtOAc (4 × 50 mL), dried over anhydrous $MgSO_4$. After filtration, the volatiles are evaporated under reduced pressure to give the residue, which was purified by silica gel column chromatography (*n*-hexane/$CHCl_3$ = 2:1) to afford **22** (935 mg, 90%) as a colorless oil. ^1H NMR (400 MHz, $CDCl_3$): δ = 1.44–1.56 (m, 2H), 1.62–1.72 (m, 4H), 2.33 (t, *J* = 7.0 Hz, 2H), 2.63 (t, *J* = 7.7 Hz, 2H), 7.16–7.30 (m, 5H).

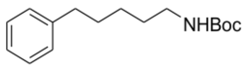

1-*tert*-Butoxycarbonylamino-5-phenylpentane (**23**).

Following the general procedure for reduction and Boc protection of nitrile derivatives, the reaction of compound **21** (478 mg, 3.0 mmol) was performed. The resulting crude mixture was purified by silica gel column chromatography ($CHCl_3$/*n*-hexane = 2:1) to afford **23** (542.5 mg, 69%) as a yellow oil. ^1H NMR (400MHz, $CDCl_3$): δ = 1.33–1.39 (m, 2H), 1.44 (s, 9H), 1.48–1.52 (m, 2H), 1.59–1.67 (m, 2H), 2.61(t, *J* = 7.7 Hz, 2H), 3.11 (q, *J* = 6.8 Hz, 2H), 4.50 (s, 1H), 7.16–7.29 (m, 5H); ^{13}C NMR (125 MHz, $CDCl_3$): δ =26.4, 28.4, 29.9, 31.1, 35.8, 40.5, 79.0, 125.7, 128.3, 128.4, 142.5, 155.9; LRMS (DART+): *m/z* $[2M+H]^+$: 527; Anal. Calcd. for $C_{16}H_{25}NO_2$: C, 72.96; H, 9.57; N, 5.32. Found: C, 72.93; H, 9.59; N, 5.28.

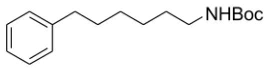

1-*tert*-Butoxycarbonylamino-6-phenylhexane (**24**).

Following the general procedure for reduction and Boc protection of nitrile derivatives, the reaction of compound **22** (693.0 mg, 4.0 mmol) was performed. The resulting crude mixture was purified by silica gel column chromatography ($CHCl_3$/*n*-hexane = 2:1) to afford **24** (694 mg, 63%) as a yellow oil. ^{1}H NMR (400 MHz, $CDCl_3$): δ = 1.33–1.35 (m, 4H), 1.44 (s, 11H), 1.62 (t, *J* = 7.2 Hz, 2H), 2.60 (t, *J* = 7.7 Hz, 2H), 3.11 (q, *J* = 6.3 Hz, 2H), 7.16–7.30 (m, 5H); ^{13}C NMR (125 MHz, $CDCl_3$): δ = 26.6, 28.4, 28.9, 30.0, 31.3, 35.8, 40.5, 79.0, 125.6, 128.2, 128.3, 142.6, 155.9; LRMS (DART+): *m/z* $[2M+H]^+$: 555; Anal. Calcd for $C_{17}H_{27}NO_2$: C, 73.61; H, 9.81; N, 5.05. Found: C, 73.51; H, 9.86; N, 4.85.

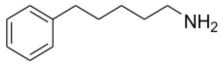

Phenylpentylamine (**6j**) [8].

Following the general procedure, the reaction of **23** (481 mg, 1.8 mmol) was performed to give **6j** (296 mg, quant.) as a pale yellow oil. ^{1}H NMR (500 MHz, $CDCl_3$): δ = 1.33–1.39 (m, 2H), 1.48–1.54 (m, 2H), 1.60–1.66 (m, 2H), 2.61 (t, *J* = 7.7 Hz, 2H), 2.71 (t, *J* = 7.2 Hz, 2H), 7.16–7.29 (m, 5H).

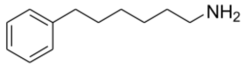

Phenylhexylamine (**6k**) [8].

Following the general procedure, the reaction of **24** (460 mg, 1.7 mmol) was performed to give **6k** (295 mg, quant.) as a pale yellow oil. ^{1}H NMR (500 MHz, $CDCl_3$): δ = 1.34 (br, 4H), 1.48 (br, 2H), 1.61 (br, 2H), 2.59 (t, *J* = 7.7 Hz, 2H), 2.69 (t, *J* = 7.2 Hz, 2H), 7.16–7.28 (m, 5H).

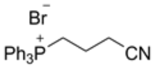

3-Cyanopropyltriphenylphosphonium bromide (**26**) [9].

A solution of triphenylphosphine (5.30 g, 20.2 mmol) and 4-bromobutyronitrile (**25**, 2.49 g, 16.8 mmol) in toluene (30 mL) was refluxed under N_2 for 18 h. The reaction mixture was cooled and the solid was collected by filtration, washed with diethyl ether, and dried under vacuum to give **26** (6.07 g, 88%) as a colorless powder. ^{1}H NMR (500 MHz $CDCl_3$): δ = 2.01–2.06 (m, 2H), 3.12 (t, *J* = 7.0 Hz, 2H), 4.13–4.19 (m, 2H), 7.71–7.89 (m, 15H).

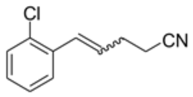

***E/Z* mixture of 5-(2-Chlorophenyl)pent-4-ennitrile** (**27**).

Compound **26** (5.91 g, 14.4 mmol) was added slowly to a round-bottomed flask containing sodium hydride (576 mg

in dry THF (60 mL) under N_2 at 0 °C. The solution was stirred at room temperature for 15 min followed by the addition of dry DMSO (6 mL). The reaction mixture was stirred for another 2 h, then 2-chlorobenzaldehyde (1.69 g, 12 mmol) was added dropwise. The solution was stirred for further 4 h. The reaction was quenched using saturated NH_4Cl aq (50 mL) and the reaction mixture was extracted with EtOAc (3 × 100 mL). The combined organic layer was dried over anhydrous $MgSO_4$ and volatiles were evaporated in vacuo. The resulting residue was purified by silica gel chromatography (*n*-hexane/EtOAc = 10 : 1) to give the compound **27** as *E/Z* mixture (1.95 g, 85%, *E* : *Z* = 1 : 4). ^{1}H NMR (500 MHz, $CDCl_3$): δ = 2.42 (t, *J* = 7.4 Hz), 2.53 (q, *J* = 6.9 Hz, triplet of *E* form was overlapped completely), 2.62 (q, *J* = 6.9 Hz), 5.80 (dt, *J* = 5.7, 13.7 Hz), 6.19 (dt, *J* = 6.9, 16.0 Hz), 6.68 (d, *J* = 11.5 Hz), 6.91 (d, *J* = 16.0 Hz), 7.19–7.51 (m, 4H).

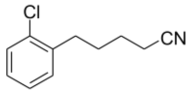

5-(2-Chlorophenyl)pentanenitrile (**28**) [10].

Pd/C (10% Pd, 26.6 mg, 5 mol%) was added to the solution of compound **27** (958 mg, 5.0 mmol) in mixed solvent (80% EtOAc and 20% 1,4-dioxane, 25 mL). The solution was stirred for 8 h at room temperature to hydrogenate under H_2 atmospheric pressure, stirred for 8 h. Then the catalyst was filtered through a cake of Celite and the filtrate was evaporated under reduced pressure. The resulting residue was purified by a silica gel column chromatography (*n*-hexane/$CHCl_3$ = 1:1) to yielded **28** (971 mg, quant.) as a colorless oil. ^{1}H NMR (500 MHz, $CDCl_3$): δ = 1.71–1.81 (m, 4H), 2.38 (t, *J* = 6.9 Hz, 2H), 2.78 (t, *J* = 7.4 Hz, 2H), 7.14–7.35 (m, 4H).

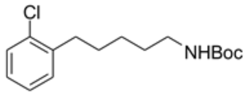

1-*tert*-Butoxycarbonylamino-5-(2-chlorophenyl)pentane (**29**).

Following the general procedure for reduction and Boc protection of nitrile derivatives, the reaction of **28** (775 mg, 4.0 mmol) was performed. The resulting crude mixture was purified by silica gel column chromatography ($CHCl_3$/*n*-hexane = 3:2) to give **29** (860 mg, 72%) as a pale yellow oil and recovered unreacted **28** (218 mg, 28%). ^{1}H NMR (500 MHz, $CDCl_3$): δ = 1.36–1.42 (m, 2H), 1.44 (s, 9H), 1.49–1.55 (m, 2H), 1.60–1.66 (m, 2H), 2.72 (t, *J* = 7.7 Hz, 2H), 3.12 (br, 2H), 4.51 (s, 1H), 7.11–7.33 (m, 4H); ^{13}C NMR (125 MHz $CDCl_3$): δ = 26.5, 28.4, 29.4, 29.9, 33.4, 40.5, 79.0, 126.7, 127.1, 129.4, 130.3, 133.8, 140.0, 155.9; LRMS (DART+): *m/z* $[2M+H]^+$: 595; Anal. Calcd. for $C_{16}H_{24}ClNO_2$: C, 64.53; H, 8.12; N, 4.70. Found: C, 64.40; H, 8.12; N, 4.57.

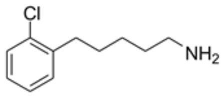

2-Chlororophenylpentylamine (**6l**).

Following the general procedure, the reaction of **29** (351 mg, 1.2 mmol) was performed to give **6l** (218 mg, 94%) as

a pale yellow oil. ^{1}H NMR (500 MHz, $CDCl_3$): δ = 1.37–1.43 (m, 2H), 1.46–1.52 (m, 2H), 1.60–1.66 (m, 2H), 2.69 (t, J = 6.9 Hz, 2H), 2.73 (t, J = 8.0 Hz, 2H), 7.11–7.33 (m, 4H); ^{13}C NMR (125 MHz, $CDCl_3$): δ = 26.6, 29.6, 33.5, 33.5, 42.1, 126.6, 127.1, 129. 4, 130.2, 133.8, 140.1; LRMS (DART+): *m/z* $[2M+H]^+$: 395; Anal. Calcd. for $C_{11}H_{17}Cl_2N \cdot 1/3H_2O$: C, 55.01; H, 7.41; N, 5.83, found: C, 55.07; H, 7.41; N 5.93.

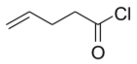

4-Pentenoyl chloride (**32**). CAS 39716-58-0

Thionyl chloride (7.14 g, 60 mmol) was slowly added to 4-pentenoic acid (**31**, 2.00 g, 20.0 mmol) at room temperature over 1 min under N_2. The mixture was stirred at 80 °C for 40 min, and then allowed to cool to room temperature. By reducing the pressure to 75 mmHg and heating to 20 °C, thionyl chloride was removed to give **32** (2.38 g, quant.). as a colorless oil. ^{1}H NMR (400 MHz, $CDCl_3$): δ = 2.39–2.48 (m, 2H), 3.00 (t, J = 7.3 Hz, 2H), 5.07–5.14 (m, 2H), 5.74–5.84 (m, 1H).

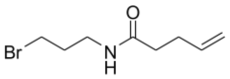

***N*-(3-Bromopropyl)pent-4-enamide** (**33**).

Anhydrous Et_3N (2.92 mL, 21 mmol) was added to the solution of compound **32** (1.02 g, 3.0 mmol) in anhydrous CH_2Cl_2 (10 mL) under N_2 at 0 °C. Then the solution of 3-bromopropylamine (1.66 mg, 14 mmol) in anhydrous CH_2Cl_2 (10 mL) was added over 15 min to the suspension and stirred for 2 h at the same temperature. The resulting suspension was warmed to room temperature, dissolved in CH_2Cl_2 (60 mL) and the organic layer was washed with 1 M HCl aq, saturated $NaHCO_3$ aq, and brine, dried over anhydrous $MgSO_4$, filtered and evaporated under reduced pressure to give the residue, which was purified by silica gel column chromatography (EtOAc/*n*-hexane = 1 : 2) to afford **33** (1.45 g, 60%) as a brown oil. ^{1}H NMR (500 MHz, $CDCl_3$): δ = 2.06–2.11 (quint, J = 6.9 Hz, 2H), 2.00–2.3 (t, J = 7.7 Hz, 2H), 2.37–2.42 (q, J = 6.9 Hz, 2H), 3.39–3.45 (m, 4H), 5.01–5.16 (m, 2H), 5.77–5.84 (m, 2H); ^{13}C NMR (125 MHz, $CDCl_3$): δ = 29.6, 31.0, 32.1, 35.8, 115.7, 137.0, 172.6; HRMS (DART+): *m/z* calcd for $C_8H_{15}BrNO^+$ $[M+H]^+$: 220.0332, found 220.0302.

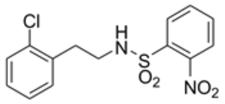

***N*-[2-(2-Chlorophenyl)ethyl]-2-nitrobenzenesulfonamide** (**30**)[11].

NEt_3 (767 µL, 5.5 mmol) was added to the solution of compound **5** (757 µL, 5.0 mmol) in CH_2Cl_2 (9 mL) at 0 °C. Then 2-nitrobenzenesulfonyl chloride (1.11 g, 5.5 mmol) was added over a period of 5 min at 0 °C. After 5 min, the reaction mixture was warmed to room temperature, stirred for 15 min, and then quenched with 1M HCl aq (10 mL). The aqueous layer was extracted with $CHCl_3$ (3 × 30 mL). The combined organic phase was washed with brine (50 mL), dried over anhydrous $MgSO_4$, filtered and evaporated to give **30** (1.71 g, quant.) as a white solid. ^{1}H NMR (500

MHz $CDCl_3$): δ = 2.98 (t, *J* = 7.2 Hz, 2H), 3.42 (q, *J* = 6.7 Hz, 2H), 1.56 (t, *J* = 5.7 Hz, 1H), 7.15–7.26 (m, 4H), 7.71–7.73 (m, 2H), 7.83–7.85 (m, 1H), 8.10–8.12 (m, 1H).

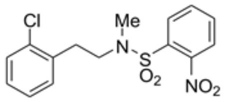

***N*-[2-(2-Chlorophenyl)ethyl]-*N*-methyl-2-nitrobenzenesulfonamide** (**34**).

Following the general procedure for introducing alkyl groups into nosyl-protected primary amines, the reaction of iodomethane (3.74 mL, 60 mmol) was performed. The resulting crude mixture was purified by silica gel column chromatography (EtOAc/*n*-hexane = 1:1) to give **34** (2.2 g, quant.); as a pale yellow oil. ^{1}H NMR (400 MHz, $CDCl_3$): δ = 2.96 (s, 3H), 3.03 (t, *J* = 8.2 Hz, 2H), 3.48 (t, *J* = 8.2 Hz, 2H), 7.10–7.22 (m, 2H), 7.26 (d, *J* = 7.2, 1H), 7.30 (d, *J* = 7.2 Hz, 1H), 7.60 (d, *J* = 7.7 Hz, 1H) 7.63–7.72 (m, 2H), 7.94 (d, *J* = 7.5 Hz, 1H); ^{13}C NMR (100 MHz, CD_3OD): δ = 32.5, 34.9, 49.7, 124.1, 127.1, 128.3, 129.5, 130.6, 131.2, 131.7, 132.3, 133.5, 133.8, 135.40, 148.0; HRMS (DART+): *m/z* calcd for $C_{15}H_{16}ClN_2O_4S^+$ $[M+H]^+$: 355.0519, found 355.0506.

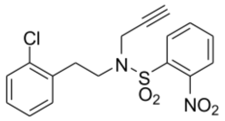

***N*-[2-(2-Chlorophenyl)ethyl]-*N*-propargyl-2-nitrobenzenesulfonamide (35)**.

Following the general procedure for introducing alkyl groups into nosyl-protected primary amines, the reaction of propargyl bromide (250 µL, 3.3 mmol) was performed. Obtained crude mixture was purified by silica gel column chromatography (EtOAc/n-hexane = 1:1) to give **35** (1.08 g, 95%); as a white solid. ^{1}H NMR (400 MHz, $CDCl_3$): δ = 2.23 (t, *J* = 2.4 Hz, 1H), 3.06 (t, *J* = 7.6 Hz, 2H), 3.67 (t, *J* =7.6 Hz, 2H), 4.24 (d, *J* = 2.4 Hz, 2H), 7.11–7.33 (m, 4H), 7.60–7.72 (m, 3H), 8.01–8.06 (m, 1H); ^{13}C NMR (100 MHz, CD_3OD): δ = 32.4, 37.0, 46.5, 73.9, 77.0, 124.3, 127.1, 128.4, 130.9, 131.2, 131.7, 132.8, 134.0, 135.3, 148.1; HRMS (DART+): *m/z* calcd for $C_{17}H_{16}ClN_2O_4S^+$ $[M+H]^+$: 379.0519, found 379.0524.

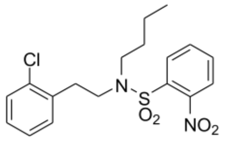

***N*-[2-(2-Chlorophenyl)ethyl]-*N*-butyl-2-nitrobenzenesulfonamide** (**36**).

Following the general procedure for introducing alkyl groups into nosyl-protected primary amines, the reaction of *n*-butyl bromide (707 µL, 18 mmol) was performed. The resulting crude mixture was purified by silica gel column chromatography (EtOAc/*n*-hexane = 1:1) to give **36** (2.5 g, quant.) as a white solid. ^{1}H NMR (500 MHz, $CDCl_3$): δ = 0.90 (t, *J* = 7.4 Hz, 3H), 1.30 (sext, *J* = 7.4 Hz, 2H), 1.54–1.60 (m, 2H), 3.00 (t, *J* = 7.9 Hz, 2H), 3.37 (t, *J* = 7.9 Hz, 2H), 3.50 (t, *J* = 7.9 Hz, 2H), 7.11–7.27 (m, 3H), 7.30 (dd, *J* = 7.4, 1.7 Hz, 1H), 7.58–7.71 (m, 3H), 7.99–8.04

(m, 1H); ^{13}C NMR (125 MHz, CD_3OD): δ = 13.4, 19.5, 29.9, 32.8, 46.5, 47.5, 124.0, 126.9, 128.1, 129.2, 130.2, 131.1, 131.6, 132.9, 133.5, 133.5, 135.4, 147.7; HRMS (DART+): *m/z* calcd for $C_{18}H_{22}ClN_2O_4S^+$ $[M+H]^+$: 397.0983, found 397.0976.

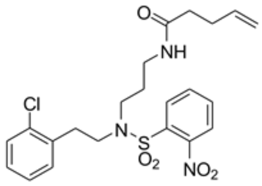

***N*-(3-(*N*-2-(2-Chlorophenyl)ethyl-2-nitrophenylsulfonamido)propyl)pent-4-enamide** (**37**).

Following the general procedure for introducing alkyl groups into nosyl-protected primary amines, the reaction of **33** (946 mg, 4.3 mmol) was performed. The resulting crude mixture was purified by silica gel column chromatography (EtOAc/*n*-hexane = 1:1) to give **37** (1.19 g, 83%) as a pale brown oil. ^{1}H NMR (500 MHz, $CDCl_3$): δ = 1.81 (quint, *J* = 6.4 Hz, 2H), 2.29 (t, *J* = 7.3 Hz, 2H), 2.40 (q, *J* = 7.3 Hz, 2H), 2.97 (t, *J* = 8.1 Hz, 2H), 3.47 (quint, *J* = 7.7 Hz, 4H), 5.01 (dd, *J* = 10.0, 1.4 Hz, 1H), 5.08 (dd, *J* = 18.0, 1.4 Hz, 1H), 5.79–5.88 (m, 1H), 6.07 (t, *J* = 6.8 Hz, 1H), 7.14–7.30 (m, 4H), 7.62–8.00 (m, 4H); ^{13}C NMR (125 MHz, CD_3OD): δ = 27.6, 29.6, 32.9, 35.7, 35.9, 45.7, 47.2, 115.5, 124.3, 127.2, 128.5, 130.6, 131.3, 131.8, 131.8, 132.1, 133.7, 133.8, 135.2, 137.0, 135.1, 172.5; HRMS (DART+): *m/z* calcd for $C_{22}H_{27}ClN_3O_5S^+$ $[M+H]^+$: 480.1355, found 480.1430.

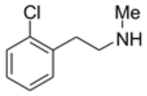

***N*-[2-(2-Chlorophenyl)ethyl]-*N*-methylamine** (**6m**). CAS 52516-17-3

Following the general procedure for deprotection of sulfonamides, the reaction of **34** (2.13 g, 6.0 mmol) was performed. The resulting crude mixture was purified by silica gel column chromatography ($CHCl_3$/*n*-hexane/NH_3 aq = 2 : 1 : 0.01) to afford **6m** (784 mg, 77%) as a pale yellow oil. ^{1}H NMR (500 MHz, CD_3OD): δ = 2.74 (s, 3H), 3.02 (t, *J* = 8.3 Hz, 2H), 3.18 (t, *J* = 8.3 Hz, 2H), 7.16–7.21 (m, 4H).

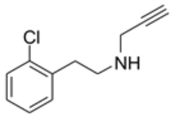

***N*-[2-(2-Chlorophenyl)ethyl]-*N*-propargylamine** (**6n**).

Following the general procedure for deprotection of sulfonamides, the reaction of **35** (947 mg, 2.5 mmol) was performed. The resulting crude mixture was purified by silica gel column chromatography ($CHCl_3$/*n*-hexane = 1 : 1) to afford **6n** (384 mg, 80%) as a brown oil. ^{1}H NMR (500 MHz, CD_3OD): δ = 2.22 (t, *J* = 2.5 Hz, 1H), 2.92–3.02 (m, 4H), 3.46 (d, *J* = 2.5 Hz, 2H), 7.13–7.22 (m, 2H), 7.20–7.27 (m, 1H), 7.35 (dd, *J* = 8.0, 1.0 Hz, 1H); ^{13}C NMR (125 MHz, $CDCl_3$): δ = 33.9, 38.1, 48.1, 71.4, 82.0, 126.8, 127.7, 129.6, 130.7, 134.1, 137.4; HRMS (DART+): *m/z* calcd for $C_{11}H_{13}ClN^+$ $[M+H]^+$: 194.0737, found 194.0738.

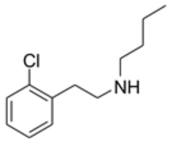

***N*-[2-(2-Chlorophenyl)ethyl]-*N*-butylamine** (**6o**).

Following the general procedure for deprotection of sulfonamides, the reaction of **36** (2.38 g, 6.0 mmol) was performed. The resulting crude mixture was purified by silica gel column chromatography ($CHCl_3$/MeOH = 10 : 1) to afford **6o** (1.16 g, 92%) as a pale yellow paste. ^{1}H NMR (500 MHz $CDCl_3$): δ = 0.95 (t, *J* = 7.4 Hz, 3H), 1.38 (sext, *J* = 7.4 Hz, 2H), 1.56 (quint, *J* = 7.4 Hz, 2H), 2.75 (t, *J* = 7.7 Hz, 2H), 2.92–2.95 (m, 2H), 3.00–3.31 (m, 2H), 7.20–7.27 (m, 2H), 7.33 (dd, *J* = 7.4, 1.7 Hz, 1H), 7.38 (dd, *J* = 7.7, 1.4 Hz, 1H); HRMS (DART+): *m/z* calcd for $C_{12}H_{19}ClN^+$ $[M+H]^+$: 212.1201, found 212.1180.

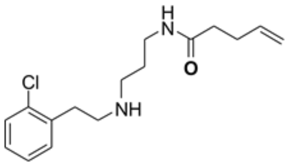

***N*-(3-(2-Nitrophenylsulfonamido)propyl)pent-4-enamide (6p)**.

Following the general procedure for deprotection of sulfonamides, the reaction of **37** (1.19 g, 2.5 mmol) was performed. The resulting crude mixture was purified by silica gel column chromatography ($CHCl_3$/*n*-hexane/NH_3 aq = 9 : 1 : 0.01) to afford **6p** (600 mg, 82%) as a pale yellow oil. ^{1}H NMR (500 MHz, $CDCl_3$): δ = 1.65 (quint, *J* = 6.4 Hz, 2H), 2.19 (t, *J* = 8.2 Hz, 2H), 2.36 (q, *J* =8.2 Hz, 2H), 2.97 (t, *J* = 8.2 Hz, 2H), 3.31 (q, *J* =5.5 Hz, 2H), 3.47 (quint, *J* = 8.2 Hz, 4H), 5.01 (dd, *J* = 10.0, 1.4 Hz, 1H), 5.08 (dd, *J* = 18.0, 1.4 Hz, 1H), 5.79–5.88 (m, 1H), 6.78 (br s, 1H), 7.15–7.27 (m, 3H), 7.35 (d, *J* = 8.2 Hz, 1H); ^{13}C NMR (125 MHz, $CDCl_3$): δ = 28.7, 29.7, 34.1, 36.0, 39.0, 48.4, 49.4, 115.4, 126.9, 127.8, 129.6, 130.7, 134.1, 137.2, 137.5, 172.2; HRMS (DART+): *m/z* calcd for $C_{16}H_{24}ClN_2O^+$ $[M+H]^+$: 295.1572, found 295.1611.

Biological evaluation

Materials and compounds. Eagle's minimum essential medium (E-MEM, 051-07615), 1 mmol/L sodium pyruvate, RPMI-1640 (with 2000 mg/L of glucose, 189-02025), Sodium Dodecyl Sulfate (SDS, 194-13985), Tween 20, bovine serum albumin (5217/100G) and 45w/v% *D*(+)-Glucose Solution (079-05511) were obtained from FUJIFILM Wako Pure Chemical Corporation (Osaka, Japan). MEM non-essential amino acid (11140050), RPMI 1640 (no glucose, 11879020) and were obtained from Thermo Fisher Scientific, Inc, (GIBCO, Tokyo, Japan). 50 units/mL penicillin, 50 μg/mL streptomycin and 50 μg/mL kanamycin were obtained from Meiji Seika Pharma Corp, Ltd, (Tokyo, Japan). G418, thiazolyl blue tetrazolium bromide (MTT), Anti-Mouse IgG (whole molecule) peroxidase conjugate (A4416) and Anti-Goat IgG (whole molecule) peroxidase conjugate (A5420) were obtained from Sigma-Aldrich (Tokyo, Japan). XF 200 mM Glutamine solution (103578-100), XF DMEM medium pH 7.4 (103575-100), XF 100 mM Pyruvate solution (103578-100), 1.5 μM oligomycin, 2 μM carbonyl cyanide-4-(trifluoromethoxy)phenylhydrazone (FCCP), 0.5 μM rotenone/antimycin A, 1 μM Oligomycin and 50 mM 2-deoxy-*D*-glucose were obtained from Agilent Technologies (Tokyo, Japan).

Preparation of test compounds. All of the test compounds were prepared as stock solutions of 100 mM in DMSO and stored in aliquots at -20 °C. The final concentration of DMSO is less than 1% (v/v) for all *in vitro* assays**.**

Cell lines and culture conditions. Cells were maintained in EMEM supplemented with 1% (v/v) MEM non-essential amino acid (100 ×) and 1 mmol/L sodium pyruvate for Human Embryo Kidney HEK293 and U87MG glioblastoma, or RPMI-1640 for human colon cancer HT-29 and A549 lung carcinoma. Both medium were supplemented with 10% heat-inactivated fetal bovine serum, 50 units/mL penicillin, 50 μg/mL streptomycin, 50 μg/mL kanamycin (Meiji Seika Pharma Corp, Ltd). All cell lines were cultured at 37°C in a humidified atmosphere containing 5% CO_2 as the normal growth condition. Cells were cultured in glucose-free medium for glucose deprivation treatment. Glucose-free RPMI 1640 medium was supplemented with 10% heat-inactivated fetal bovine serum. For hypoxia stress conditions, an air-tight chamber (Modular Incubator Chamber from Billups-Rothenberg Inc.) filled with mixed gas (1% O_2, 94% N_2, and 5% CO_2). All compounds were added at various final concentrations immediately after replacing glucose-free medium, whereas hypoxic treatment was performed one hour after the addition of compounds.

Cell viability assay. HT29 cells were seeded in 96-well (3.0 × 10^3 cells per well) and cultured overnight. Then treated with various concentrations of compounds in the normal or glucose-free medium for 48 h. Then the medium was replaced with fresh growth medium, and cells were cultured for further 16 h. Subsequently, 10 μL of thiazolyl blue tetrazolium bromide (Sigma-Aldrich, St Louis, MO, USA) solution (0.5 mg/mL) was added to each well. After 4 h incubation at 37°C, the medium was removed, 100 μL of DMSO was added then absorbance of each well was measured at 570 nm by MULTISKAN JX plate reader. Relative cell survival (mean ± SD of triplicate determinations) was calculated by setting each of the control absorbance from non-drug treated cells as 100%.

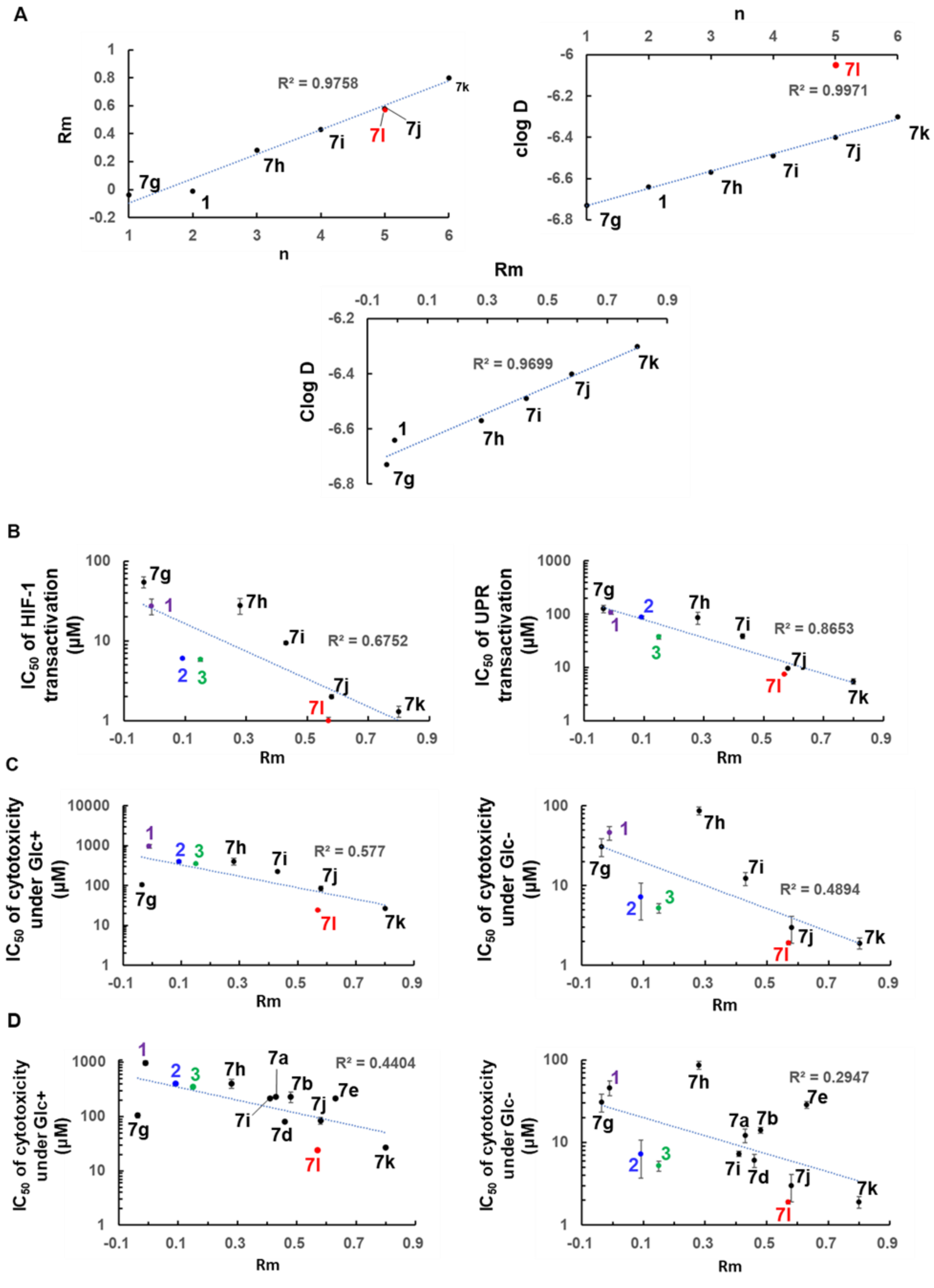

Figure S2. (A) Correlation between clog D, Rm values and alkylene linker length for compounds **1** and **7g–7k**. (B)

Correlation between Rm values and the IC_{50} values of HIF-1 or UPR transactivation for compounds **1–3** and **7g–7l**. (C) Correlation between Rm values and the IC_{50} values of cytotoxicity under Glc + or Glc − for compounds **1–3** and **7g–7l**. (D) Correlation between Rm values and the IC_{50} values of cytotoxicity under Glc + or Glc − for compounds **1–3**, **7a**, **7b**, **7d**, **7e** and **7g–7l**. The R^2 values were calculated by linear approximation in (A) or (B) and exponential approximation in (C) or (D).

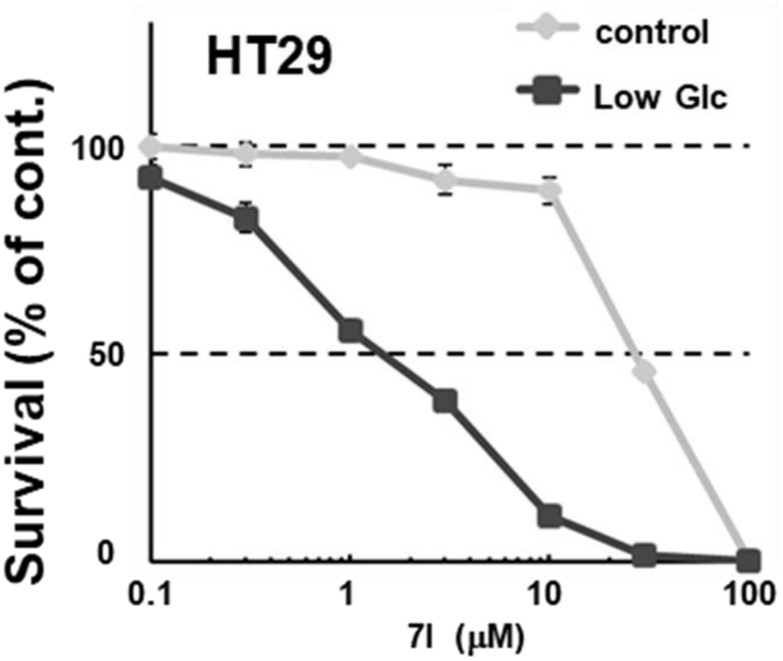

Figure S3. Concentration response curves in cell viability assay on HT29 with **7l** in normal or glucose-free medium. MTT assay using HT29 cells was performed with **7l** under normal or glucose deprivation conditions for 48 hours. Each point represents mean ± SD of triplicate experiments.

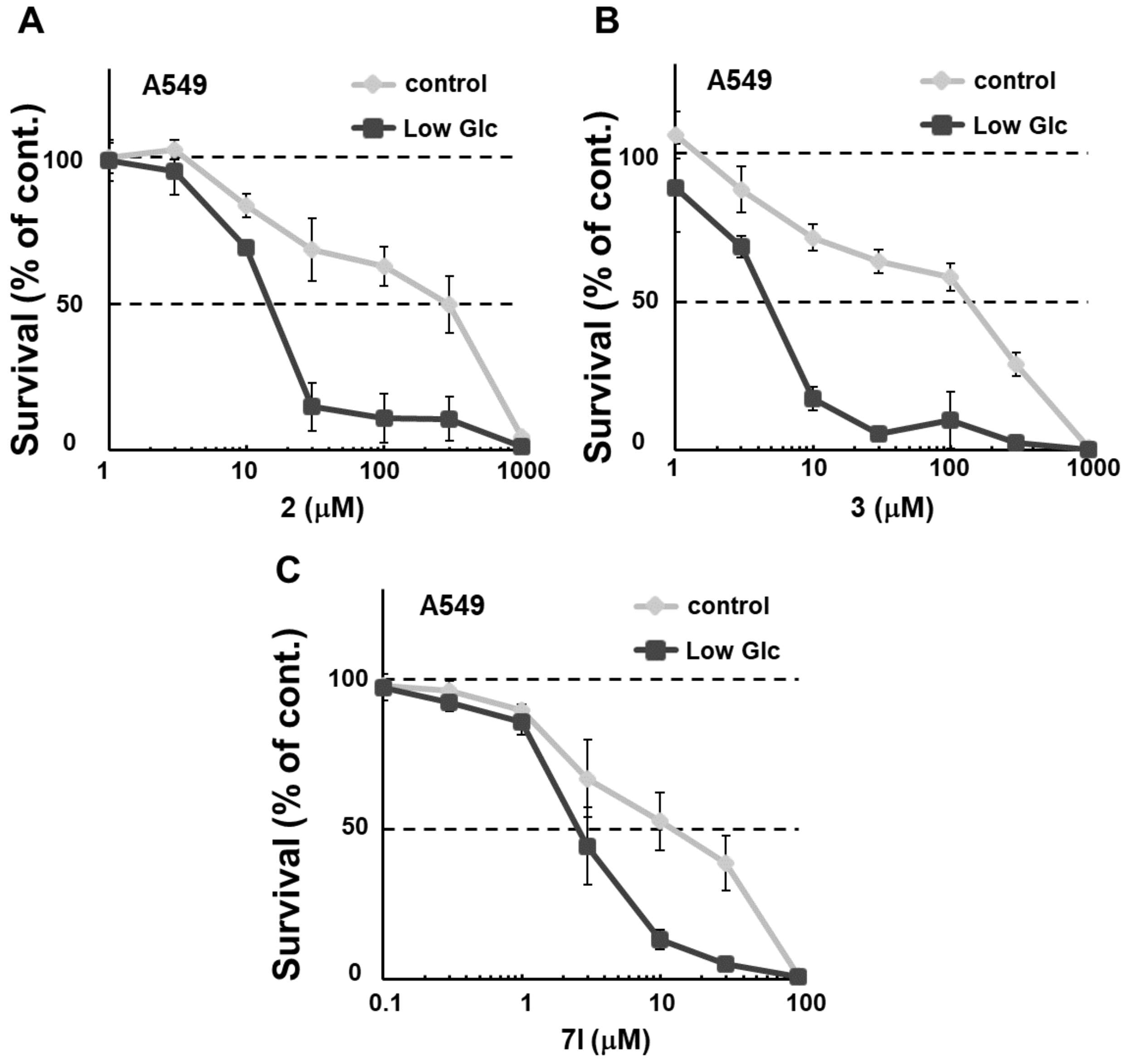

Figure S4. Concentration response curves in cell viability assay on A549 cells with **2**, **3**, and **7l** in normal or glucose-free medium. MTT assay using A549 cells was performed with **2** (A), **3** (B) and **7l** (C) under normal or glucose deprivation conditions for 48 hours. Each point represents mean ± SD of triplicate experiments.

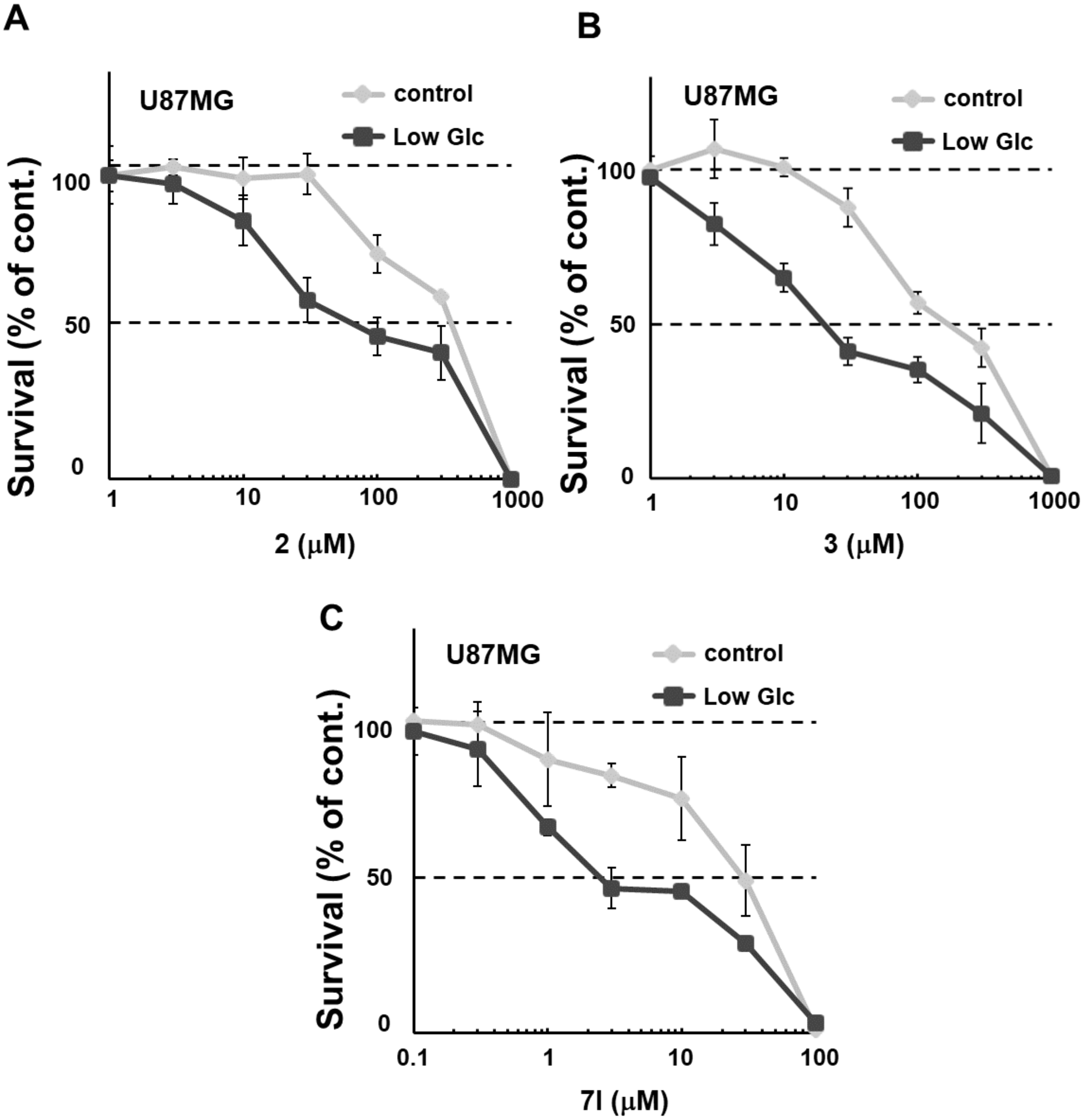

Figure S5. Concentration response curves in cell viability assay on U87MG cells with **2**, **3**, and **7l** in normal or glucose-free medium. MTT assay using U87MG cells was performed with **2** (A), **3** (B) and **7l** (C) under normal or glucose deprivation conditions for 48 hours. Each point represents mean ± SD of triplicate experiments.

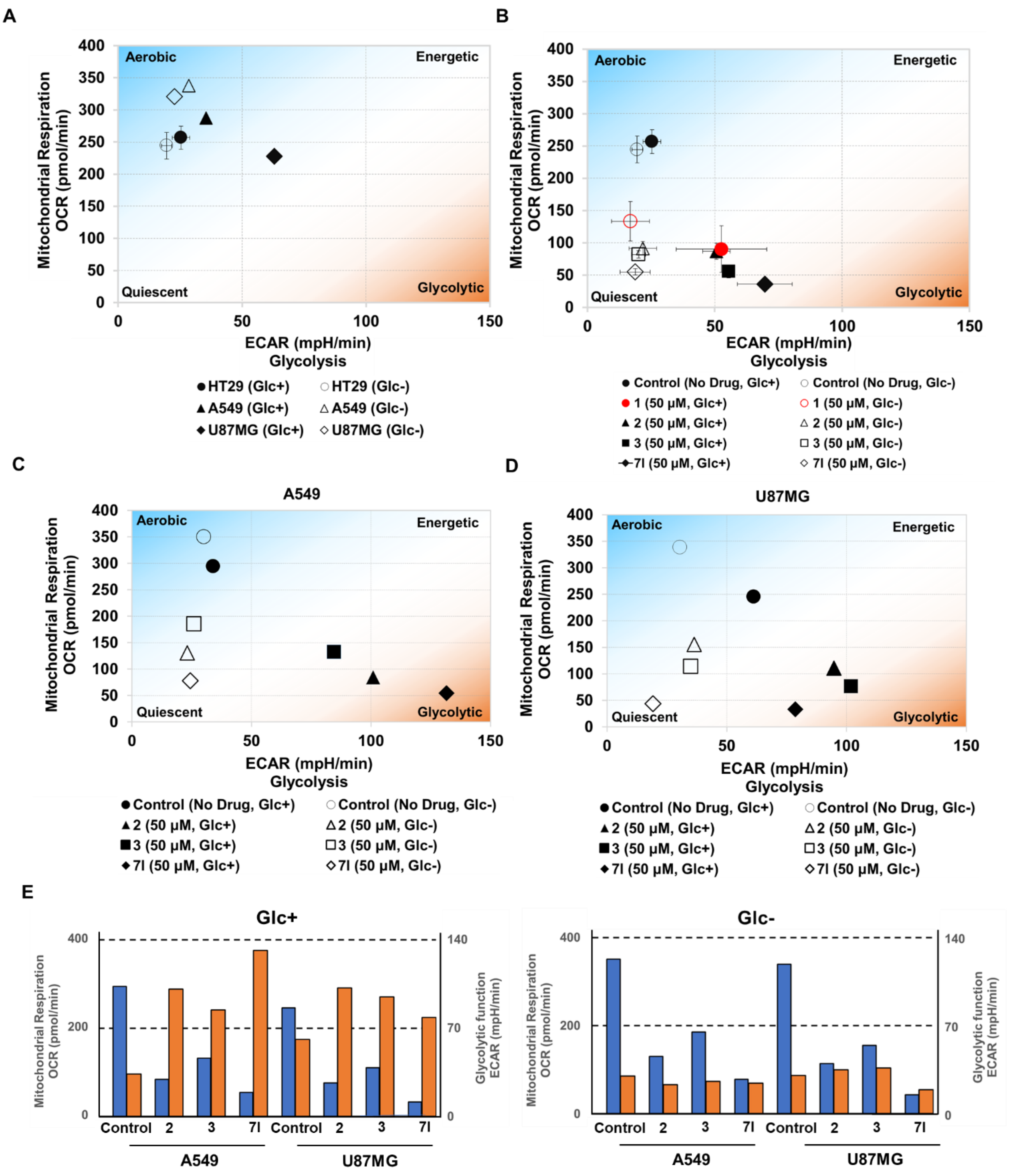

Figure S6. Cell energy phenotype profiles of HT29, A549 and U87MG. (A) Diagram of energy phenotypes of HT29, A549 and U87MG cells under normal (Glc+) or glucose free (Glc−) conditions. The energy phenotype of HT29 (B), A549 cells (C), and U87MG cells (D) treated with **2**, **3**, and **7l**. (E) OCR (Blue) and ECAR (Orange) of A549 and U87MG cells treated with treated with **2**, **3**, and **7l** under normal (Glc+) or glucose free (Glc−) conditions. Results for U87MG and A549 are from a single measurement.

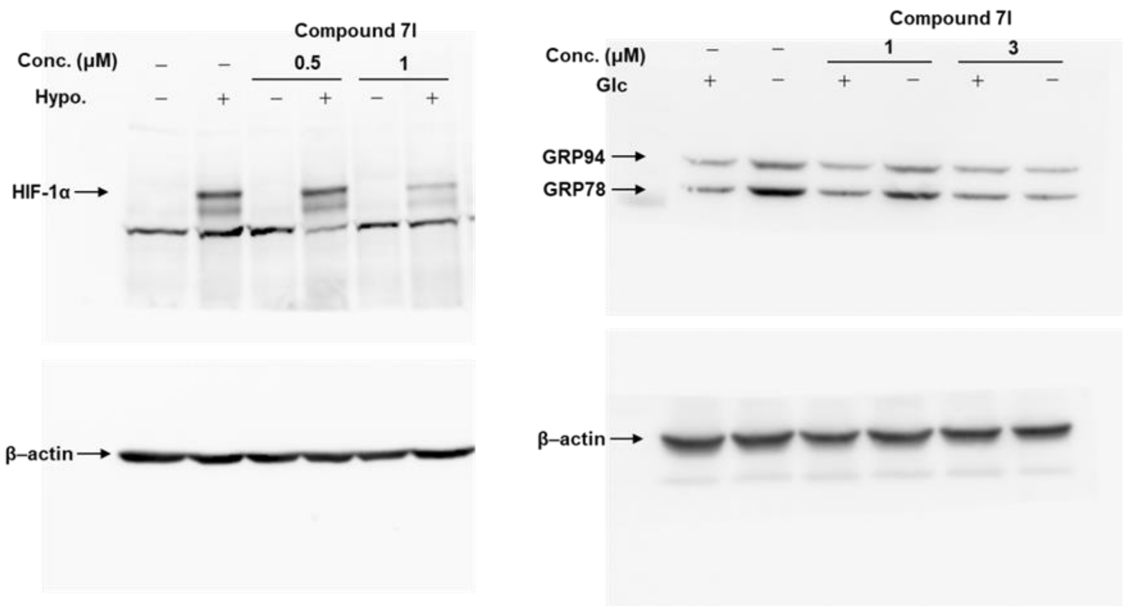

Figure S7. Full size image of the blots shown in figure 4 in main manuscript. The blot membranes were cut at approximately 50 kDa and stained with anti-HIF-1 alpha (H1alpha67) Antibody, anti-KDEL Mouse mAb, and goat polyclonal anti-human β-actin Ab (I-19) respectively. Next, they were stained with the corresponding secondary antibodies, subjected to chemiluminescence reaction and photographed with LAS3000.

References

1. Leftheris, K. et al. The discovery of orally active triaminotriazine aniline amides as inhibitors of p38 MAP kinase. *J. Med. Chem.* **47**, 6283-6291 (2004).
2. Bock, M. G., Gaul, C., Gummadi, V. R., Moebitz, H. & Sengupta, S. Preparation of aryl and heteroaryl fused piperidinone compounds as therapeutic 17α-hydroxylase/C17,20-lyase inhibitors. WO2012035078A1 (2012).
3. Murai, Y. et al. Synthesis of photoreactive 2-phenethylamine derivatives: synthesis of adenosine derivatives enabling functional analysis of adenosine receptors by photoaffinity labeling. *Eur. J. Org. Chem.* **2013**, 2428-2433 (2013).
4. Subbarao, K. V., Damodaran, N. P. & Dev, S. Photochemical transformations — V a: Organic iodides (part 4) : solution photochemistry of 4-phenyl-l-iodobotane and 4-phenyl-l-bromobutane. *Tetrahedron* **43**, 2543-2548 (1987).
5. Lai, G., Tan, P.-Z. & Ghoshal, P. A One-Pot method for the efficient conversion of Aryl- and Acyl-substituted methyl alcohols into chlorides. *Synth. Commun.* **33**, 1727-1732 (2003).
6. Vechorkin, O., Proust, V. r. & Hu, X. Functional group tolerant Kumada−Corriu−Tamao coupling of nonactivated alkyl halides with aryl and heteroaryl nucleophiles: catalysis by a nickel pincer complex permits the coupling of functionalized grignard reagents. *J. Am. Chem. Soc.* **131**, 9756-9766 (2009).
7. Lee, J.-Y. & Fu, G. C. Room-temperature Hiyama cross-couplings of arylsilanes with alkyl bromides and iodides. *J. Am. Chem. Soc.* **125**, 5616-5617 (2003).
8. Butini, S. et al. Discovery of potent inhibitors of human and mouse fatty acid amide hydrolases. *J. Med. Chem.* **55**, 6898-6915 (2012).
9. Leach, C. A. & Smith, S. A. Preparation of pyridinone and pyrimidinone compounds as Lp-PLA2 inhibitors for treating atherosclerosis. WO2003042206A1 (2003).
10. Bunnett, J. F. & Skorcz, J. A. Homocyclic ring closures via benzyne intermediates. A new synthesis of 1-substituted benzocyclobutenes. *J. Org. Chem.* **27**, 3836-3843 (1962).
11. Kurosawa, W., Kan, T. & Fukuyama, T. Preparation of secondary amines from primary amines *via* 2-nitrobenzenesulfonamides: *N*-(4-methoxybenzyl)-3-phenylpropylamine. *Org. Synth.* **79**, 186-193 (2002).

^{1}H and ^{13}C NMR spectra of new biguanides

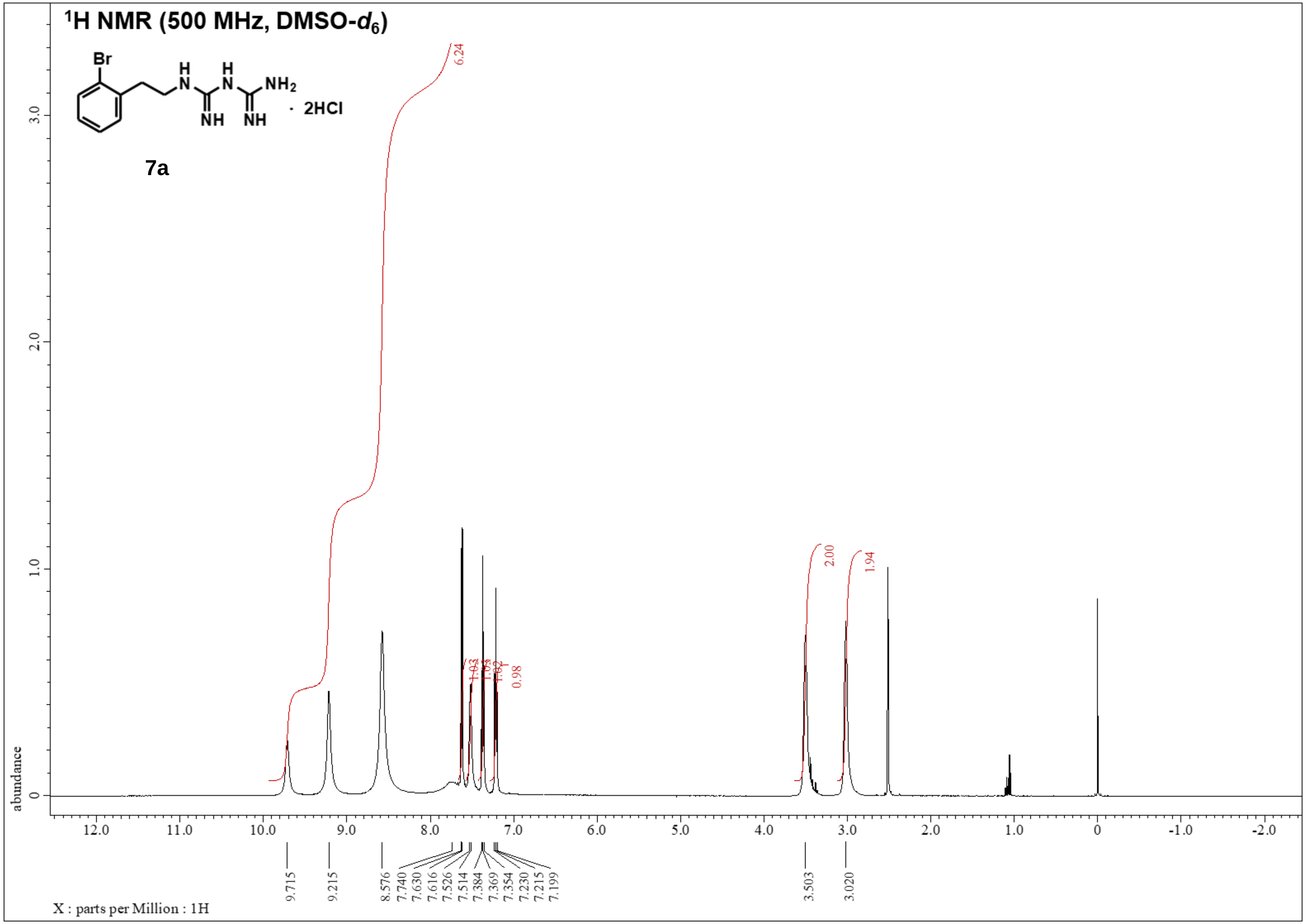

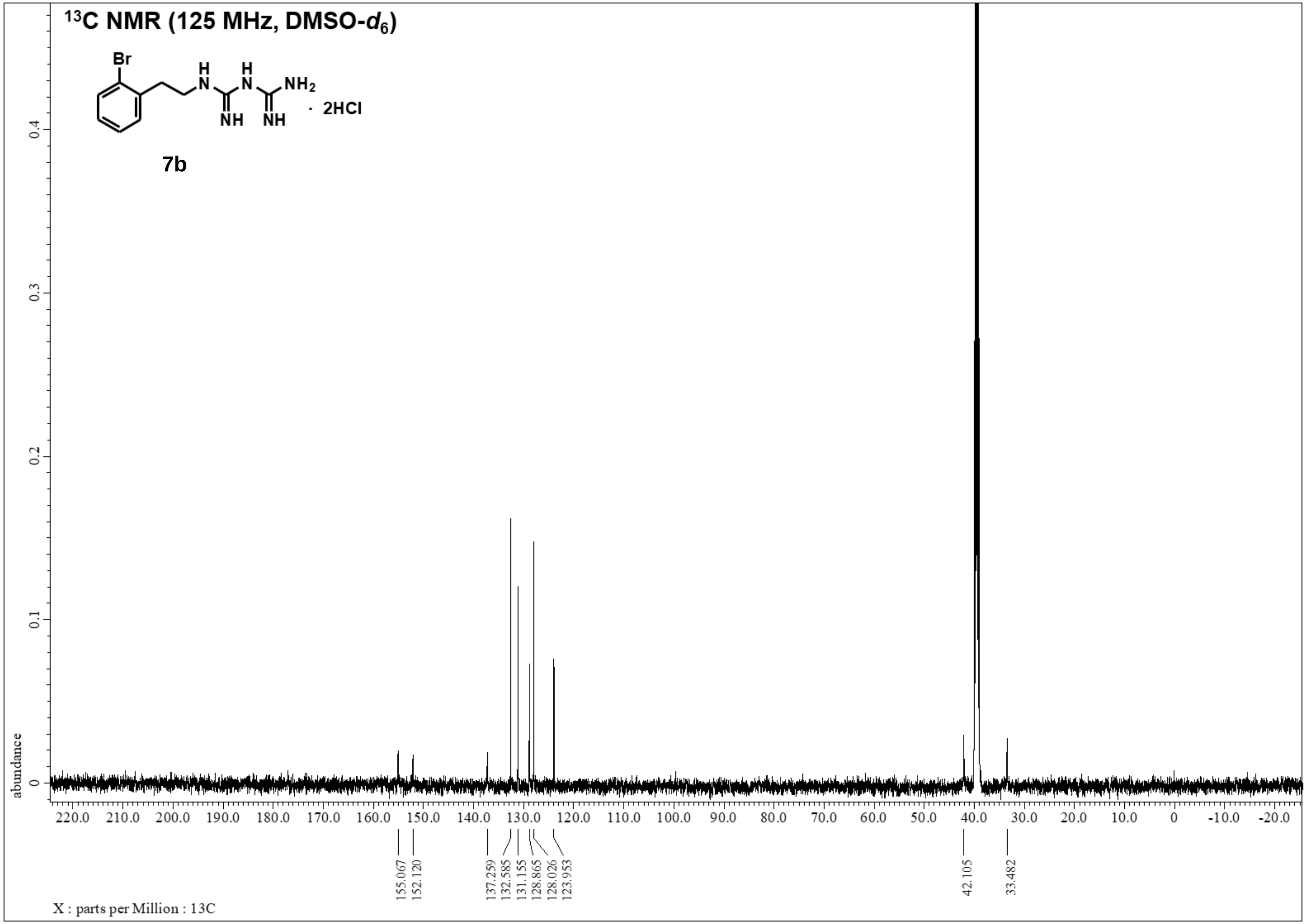

^{13}C NMR (125 MHz, DMSO-d_6)
Br
NH$_2$
NH
NH
· 2HCl
7b
abundance
0
0.1
0.2
0.3
0.4
220.0 210.0 200.0 190.0 180.0 170.0 160.0 150.0 140.0 130.0 120.0 110.0 100.0 90.0 80.0 70.0 60.0 50.0 40.0 30.0 20.0 10.0 0 -10.0 -20.0
155.067
152.120
137.259
132.585
131.155
128.865
128.026
123.953
42.105
33.482
X : parts per Million : 13C

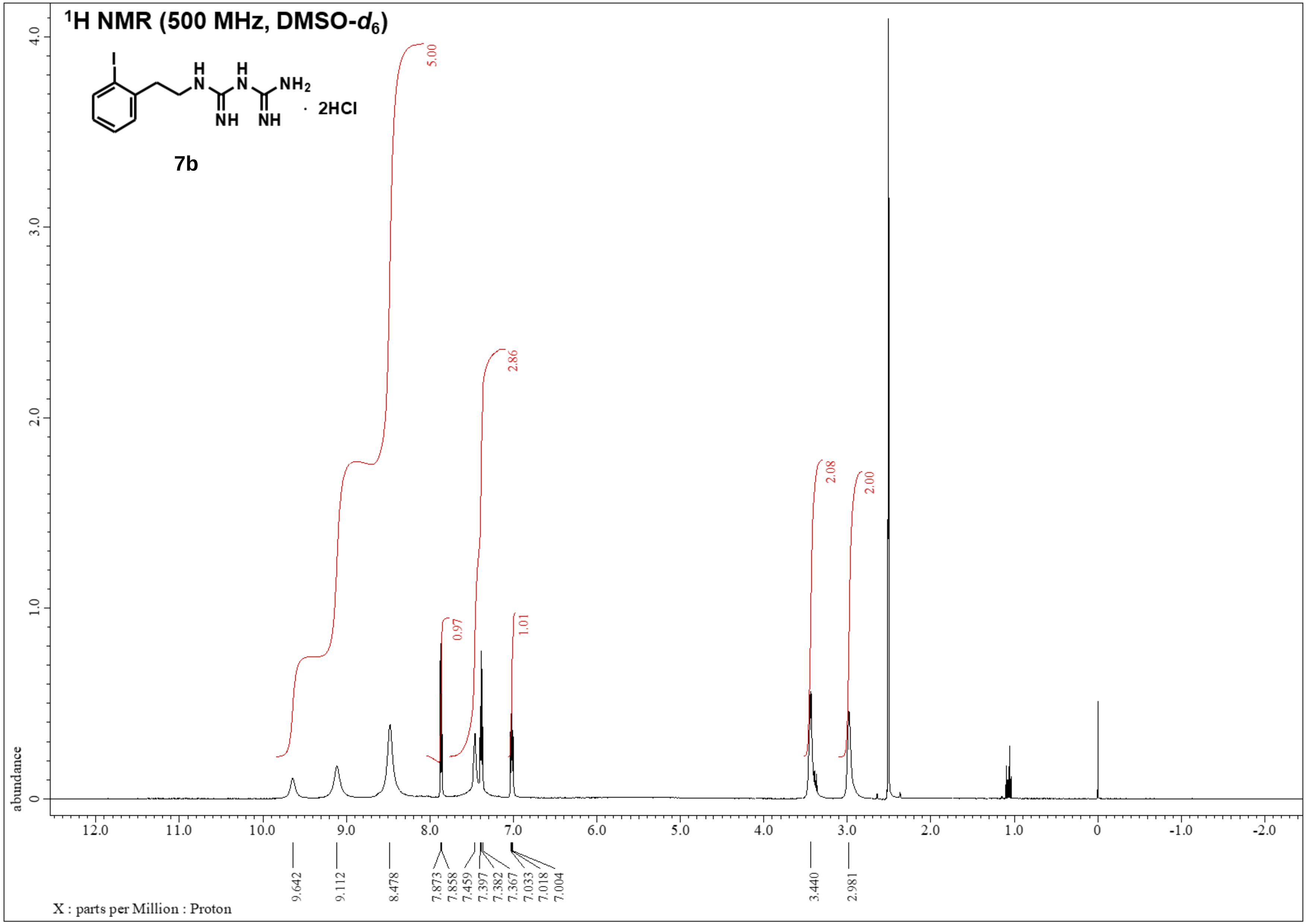

^{1}H NMR (500 MHz, DMSO-d_6)
NH$_2$
NH
NH
· 2HCl
7b
5.00
2.86
0.97
1.01
2.08
2.00
abundance
X : parts per Million : Proton
9.642
9.112
8.478
7.873
7.858
7.459
7.397
7.382
7.367
7.033
7.018
7.004
3.440
2.981

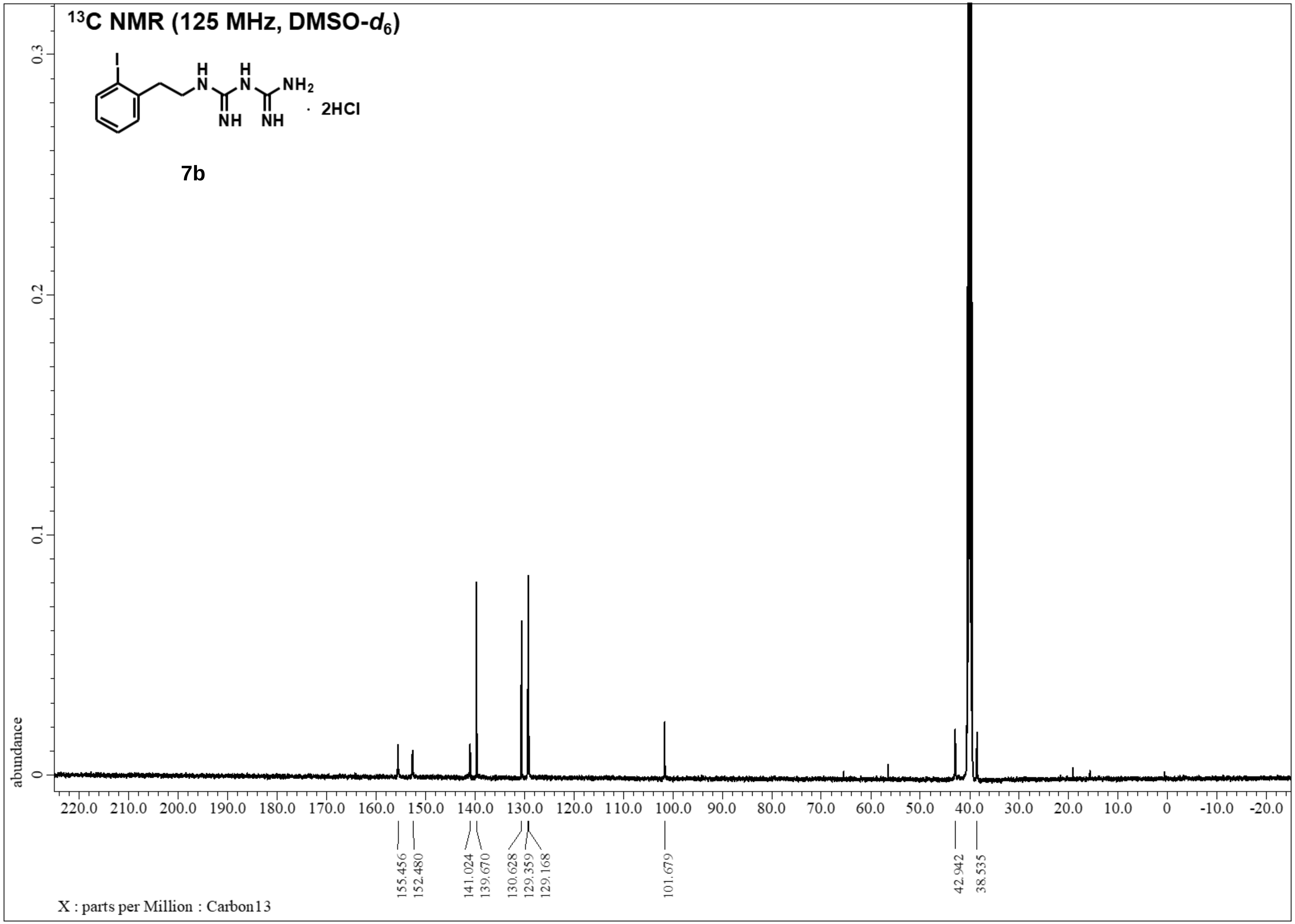

^{13}C NMR (125 MHz, DMSO-d_6)
7b
· 2HCl
abundance
0.3
0.2
0.1
0
220.0 210.0 200.0 190.0 180.0 170.0 160.0 150.0 140.0 130.0 120.0 110.0 100.0 90.0 80.0 70.0 60.0 50.0 40.0 30.0 20.0 10.0 0 -10.0 -20.0
155.456
152.480
141.024
139.670
130.628
129.359
129.168
101.679
42.942
38.535
X : parts per Million : Carbon13

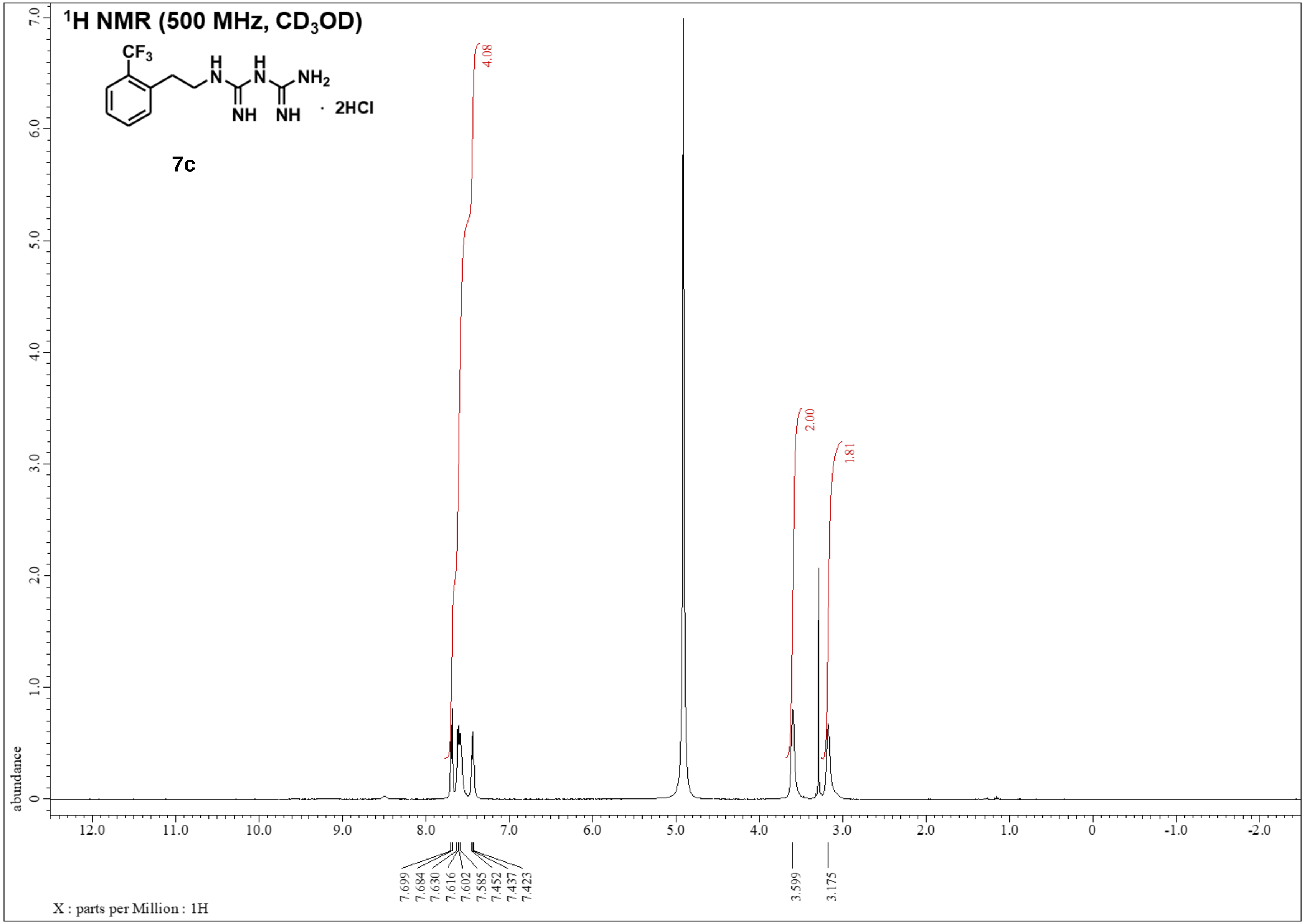

¹H NMR (500 MHz, CD₃OD)
7c
· 2HCl
4.08
2.00
1.81
7.699
7.684
7.630
7.616
7.602
7.585
7.452
7.437
7.423
3.599
3.175
abundance
X : parts per Million : 1H

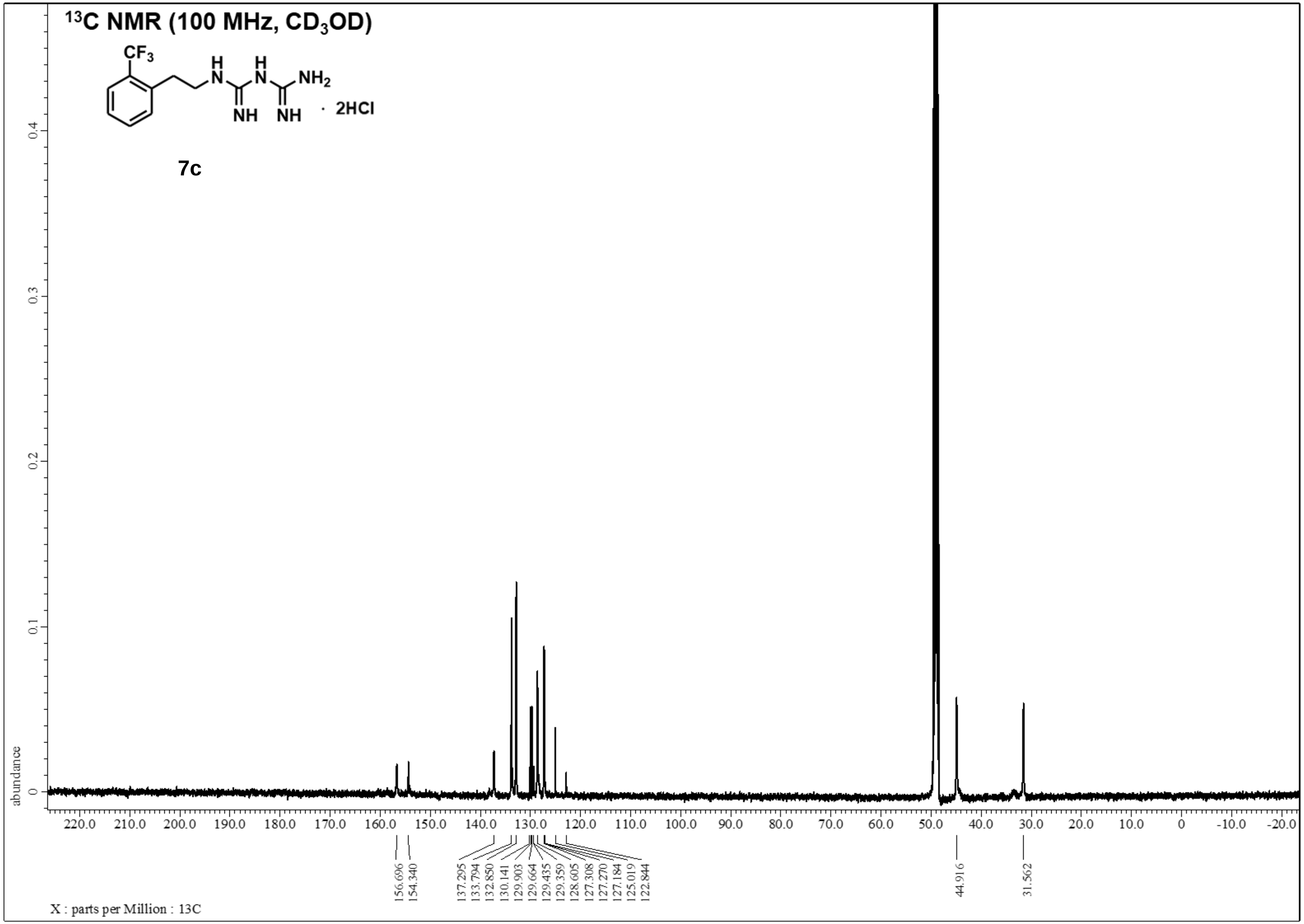

^{13}C NMR (100 MHz, CD_3OD)
7c
abundance
X : parts per Million : 13C
156.696
154.340
137.295
133.794
132.850
130.141
129.903
129.664
129.435
129.359
128.605
127.308
127.270
127.184
125.019
122.844
44.916
31.562

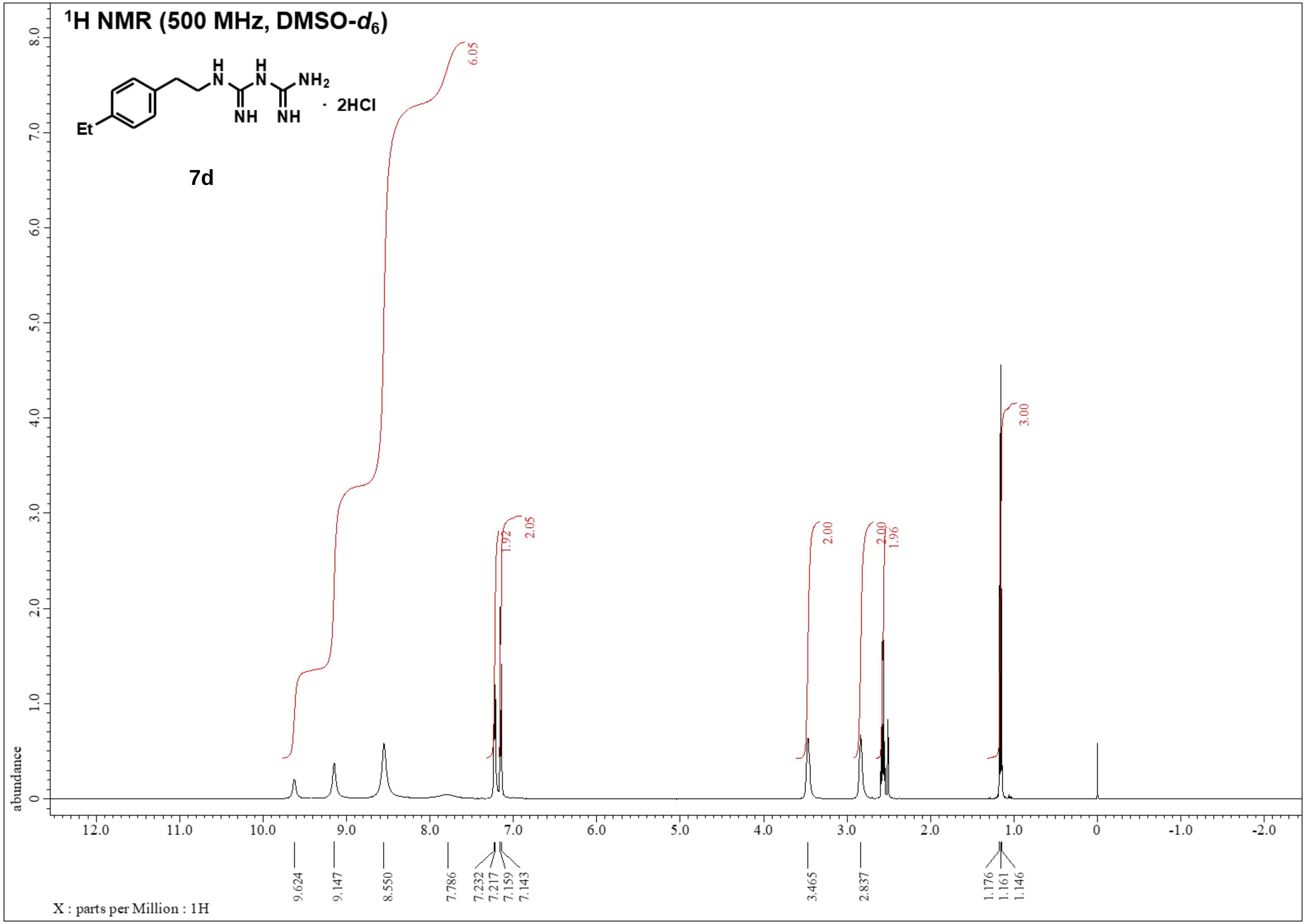

^{1}H NMR (500 MHz, DMSO-d6)
Et
NH2
NH
NH
· 2HCl
7d
6.05
1.92
2.05
2.00
2.00
1.96
3.00
abundance
X : parts per Million : 1H
9.624
9.147
8.550
7.786
7.232
7.217
7.159
7.143
3.465
2.837
1.176
1.161
1.146

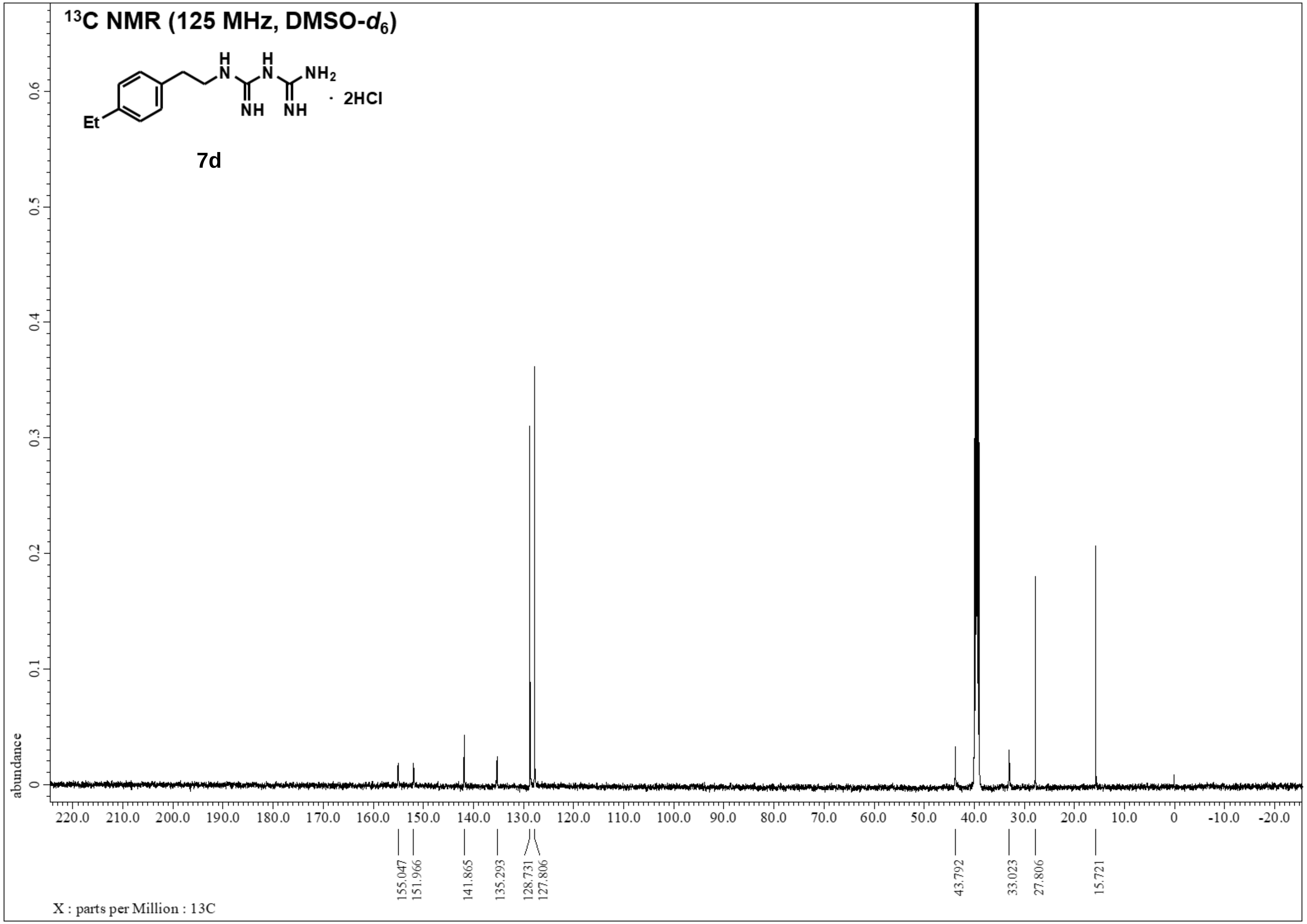

13C NMR (125 MHz, DMSO-d6)
NH2
NH
NH
· 2HCl
Et
7d
abundance
0
0.1
0.2
0.3
0.4
0.5
0.6
220.0 210.0 200.0 190.0 180.0 170.0 160.0 150.0 140.0 130.0 120.0 110.0 100.0 90.0 80.0 70.0 60.0 50.0 40.0 30.0 20.0 10.0 0 -10.0 -20.0
155.047
151.966
141.865
135.293
128.731
127.806
43.792
33.023
27.806
15.721
X : parts per Million : 13C

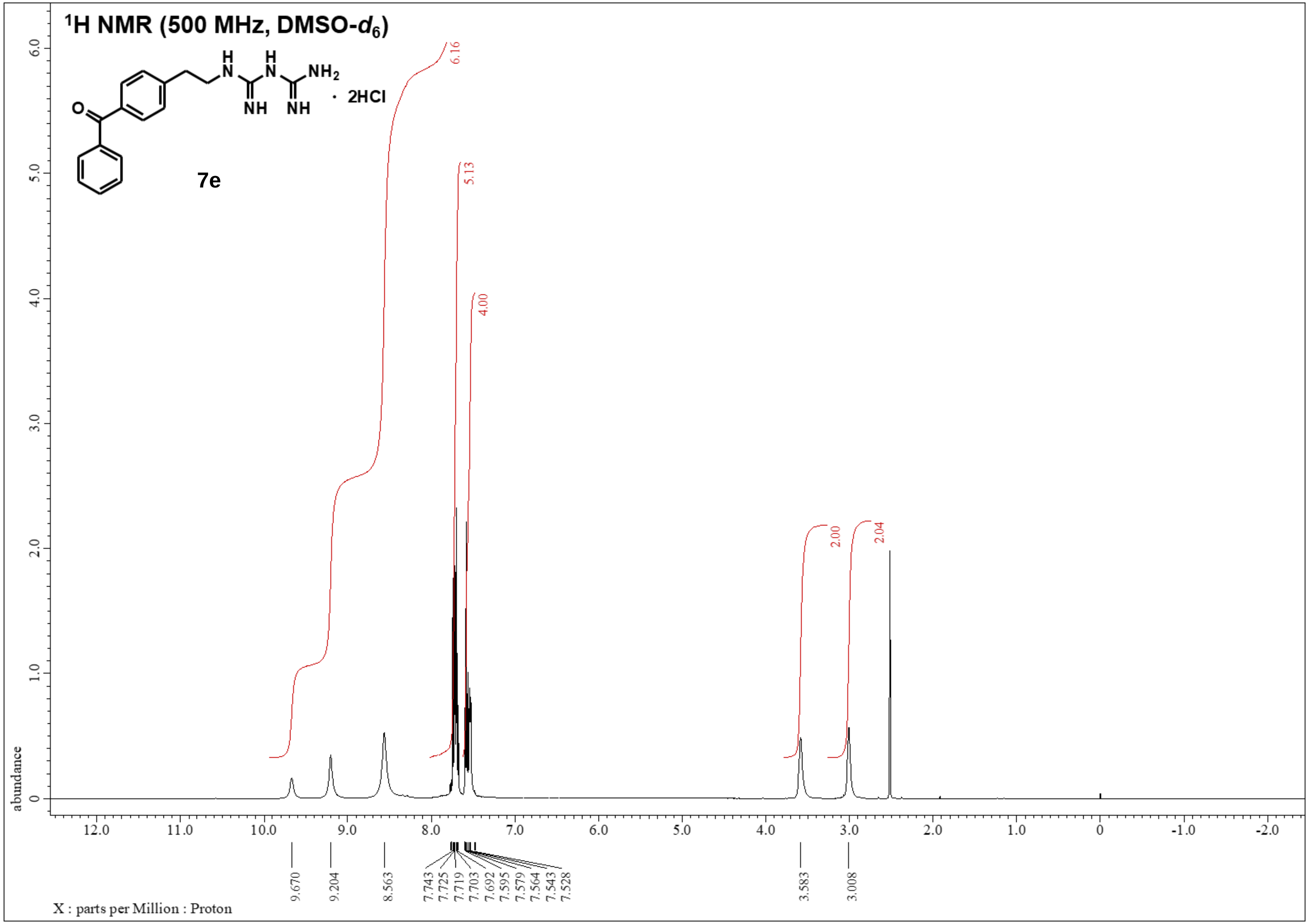

^{1}H NMR (500 MHz, DMSO-d_6)
NH
NH
NH$_2$
NH
NH
O
· 2HCl
7e
6.16
5.13
4.00
2.00
2.04
abundance
X : parts per Million : Proton
9.670
9.204
8.563
7.743
7.725
7.719
7.703
7.692
7.595
7.579
7.564
7.543
7.528
3.583
3.008

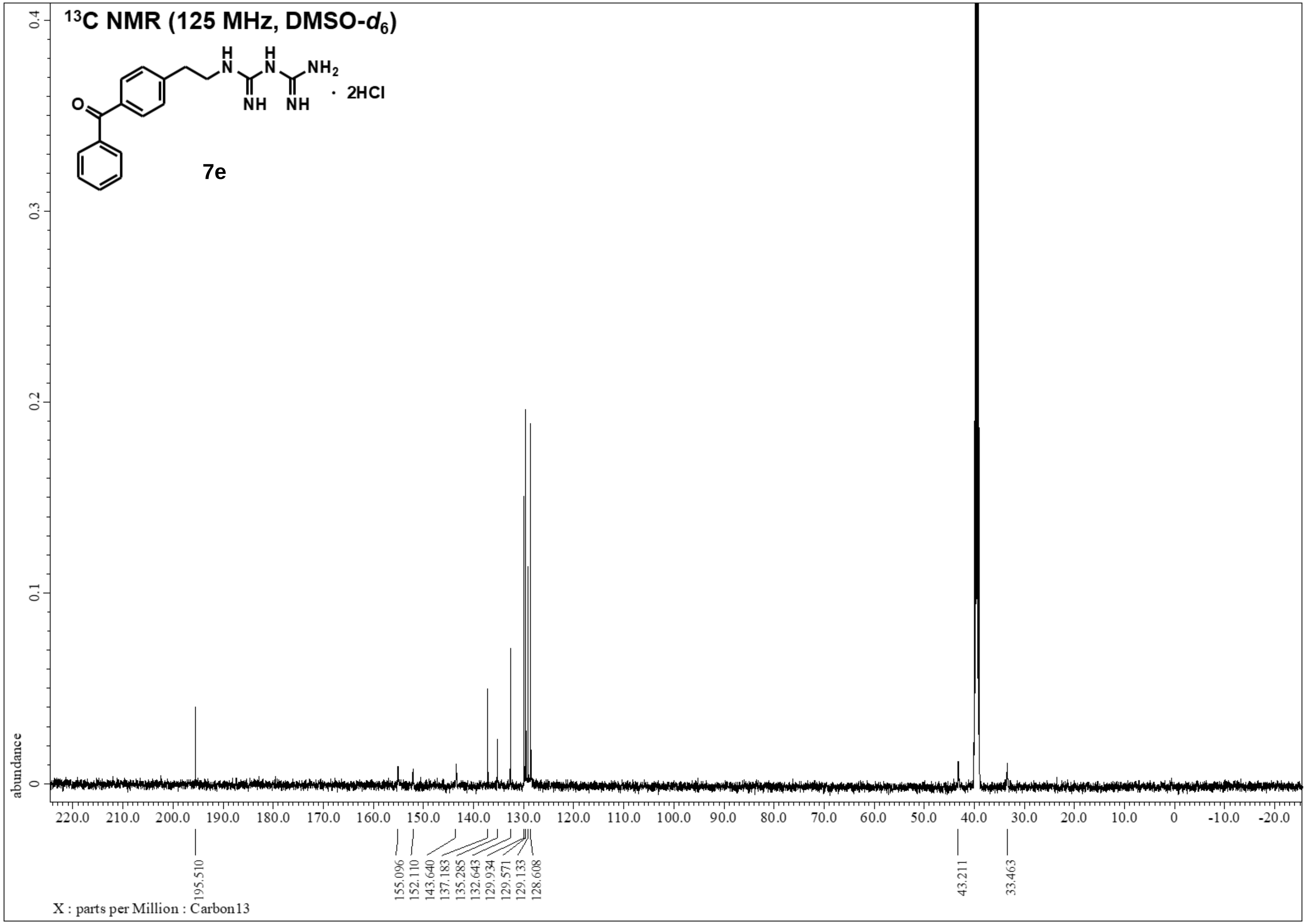

13C NMR (125 MHz, DMSO-d6)
7e
· 2HCl
abundance
195.510
155.096
152.110
143.640
137.183
135.285
132.643
129.934
129.571
129.133
128.608
43.211
33.463
X : parts per Million : Carbon13

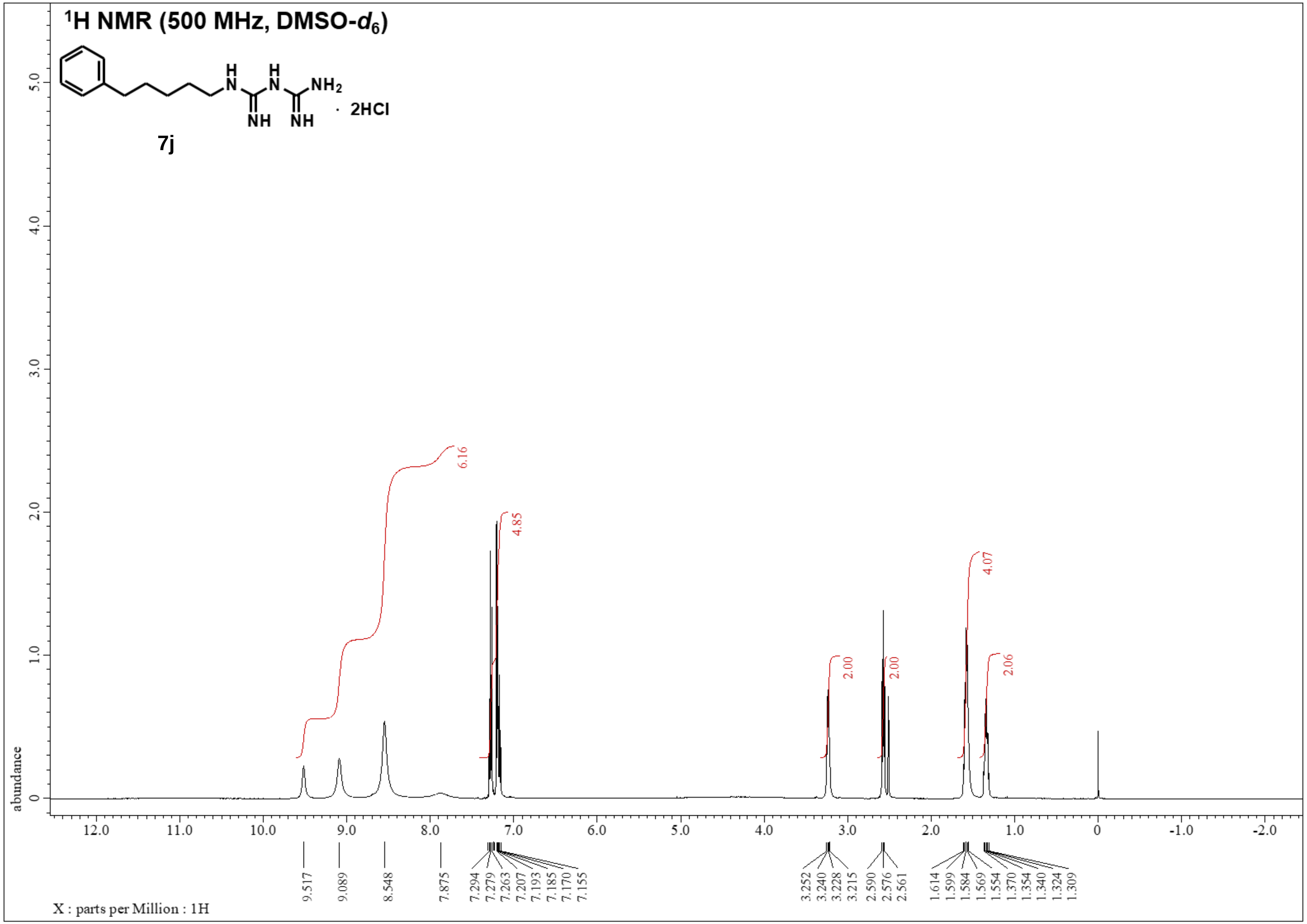

1H NMR (500 MHz, DMSO-d6)
NH2
NH NH
H H
N N
· 2HCl
7j
abundance
X : parts per Million : 1H
6.16
4.85
2.00
2.00
4.07
2.06
9.517
9.089
8.548
7.875
7.294
7.279
7.263
7.207
7.193
7.185
7.170
7.155
3.252
3.240
3.228
3.215
2.590
2.576
2.561
1.614
1.599
1.584
1.569
1.554
1.370
1.354
1.340
1.324
1.309

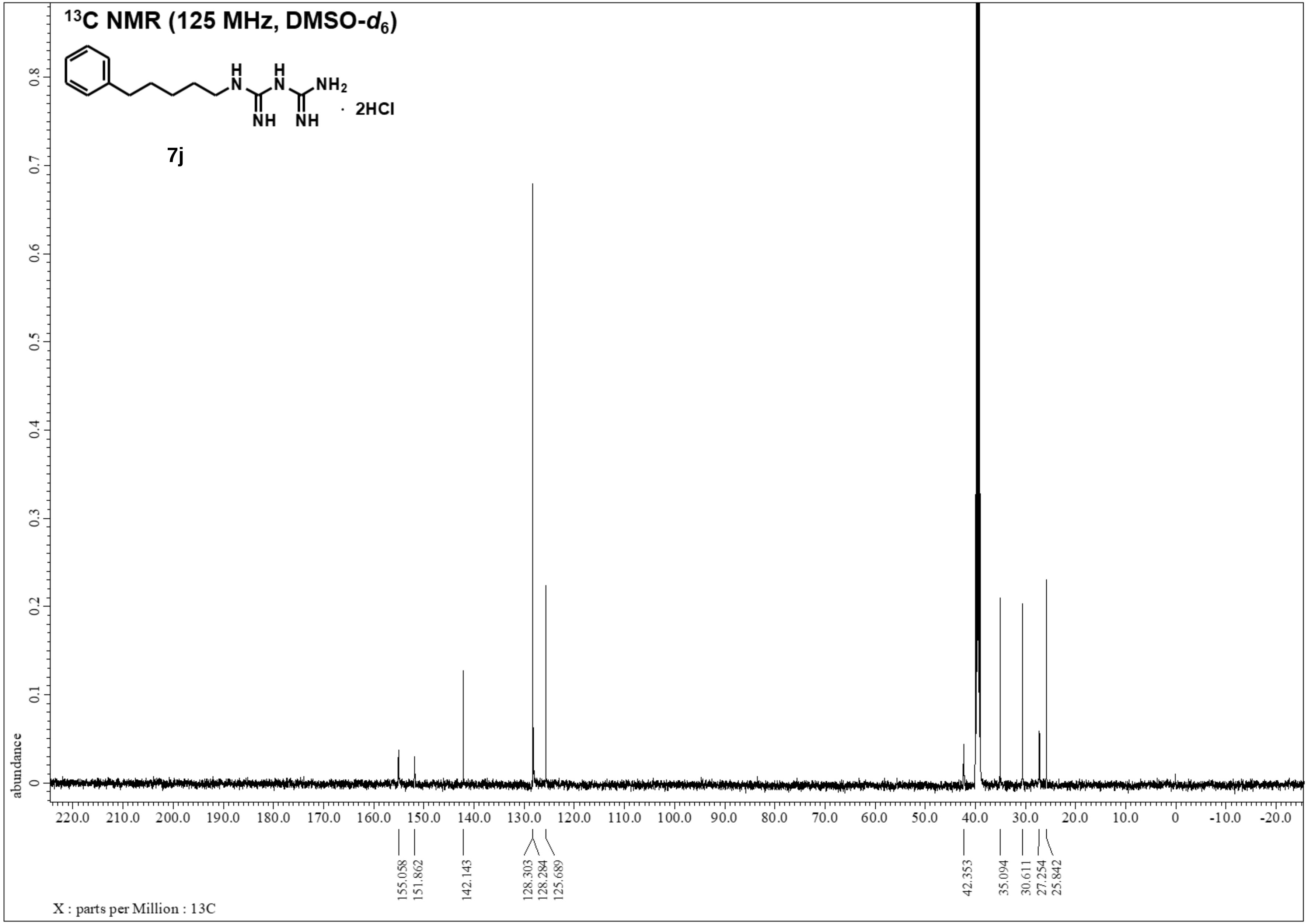

13C NMR (125 MHz, DMSO-d6)
H
N
H
N
NH2
NH
NH
· 2HCl
7j
abundance
0
0.1
0.2
0.3
0.4
0.5
0.6
0.7
0.8
220.0
210.0
200.0
190.0
180.0
170.0
160.0
150.0
140.0
130.0
120.0
110.0
100.0
90.0
80.0
70.0
60.0
50.0
40.0
30.0
20.0
10.0
0
-10.0
-20.0
155.058
151.862
142.143
128.303
128.284
125.689
42.353
35.094
30.611
27.254
25.842
X : parts per Million : 13C

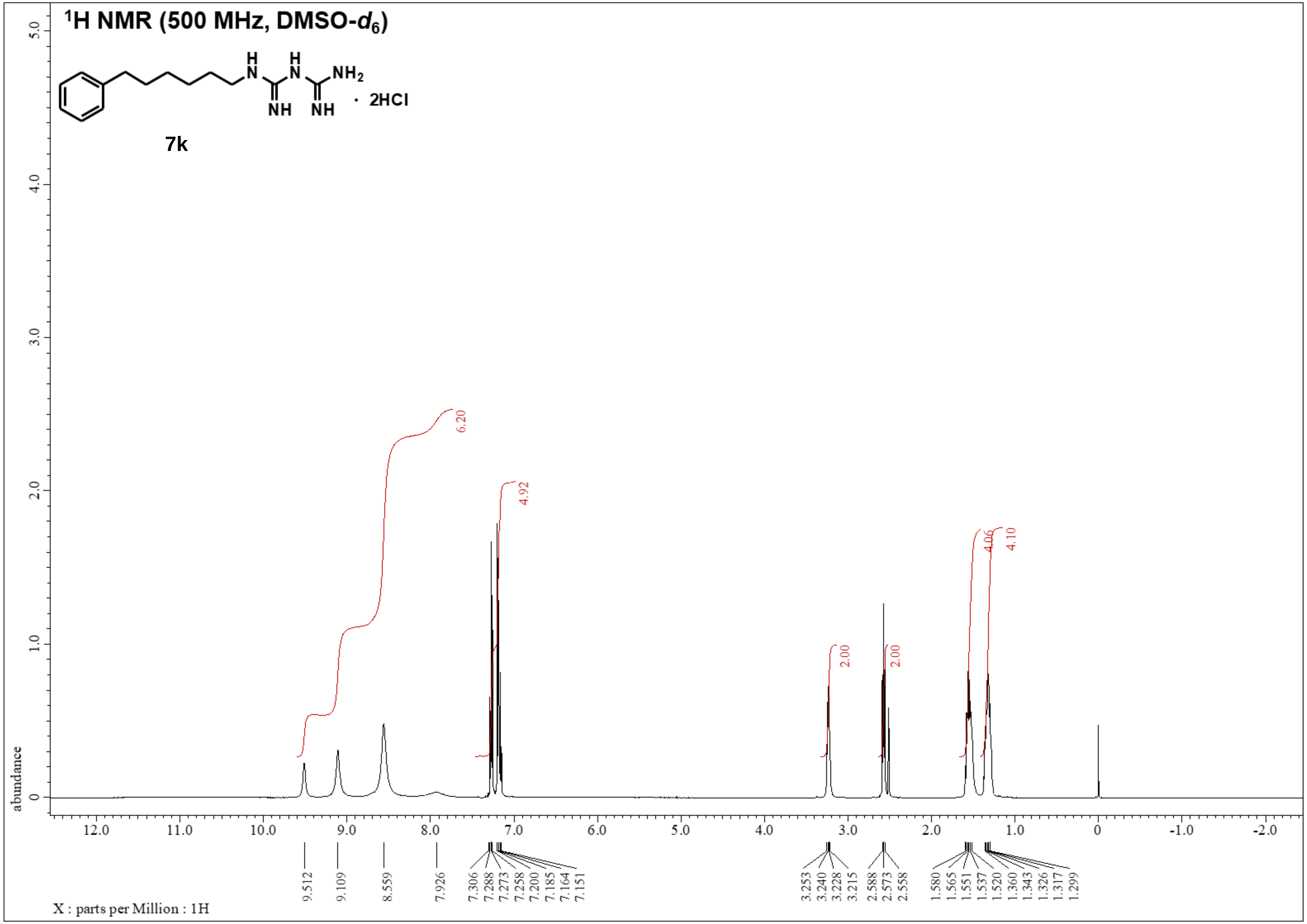

^{1}H NMR (500 MHz, DMSO-d_6)
NH$_2$
NH
NH
· 2HCl
7k
6.20
4.92
2.00
2.00
4.06
4.10
abundance
X : parts per Million : 1H
9.512
9.109
8.559
7.926
7.306
7.288
7.273
7.258
7.200
7.185
7.164
7.151
3.253
3.240
3.228
3.215
2.588
2.573
2.558
1.580
1.565
1.551
1.537
1.520
1.360
1.343
1.326
1.317
1.299

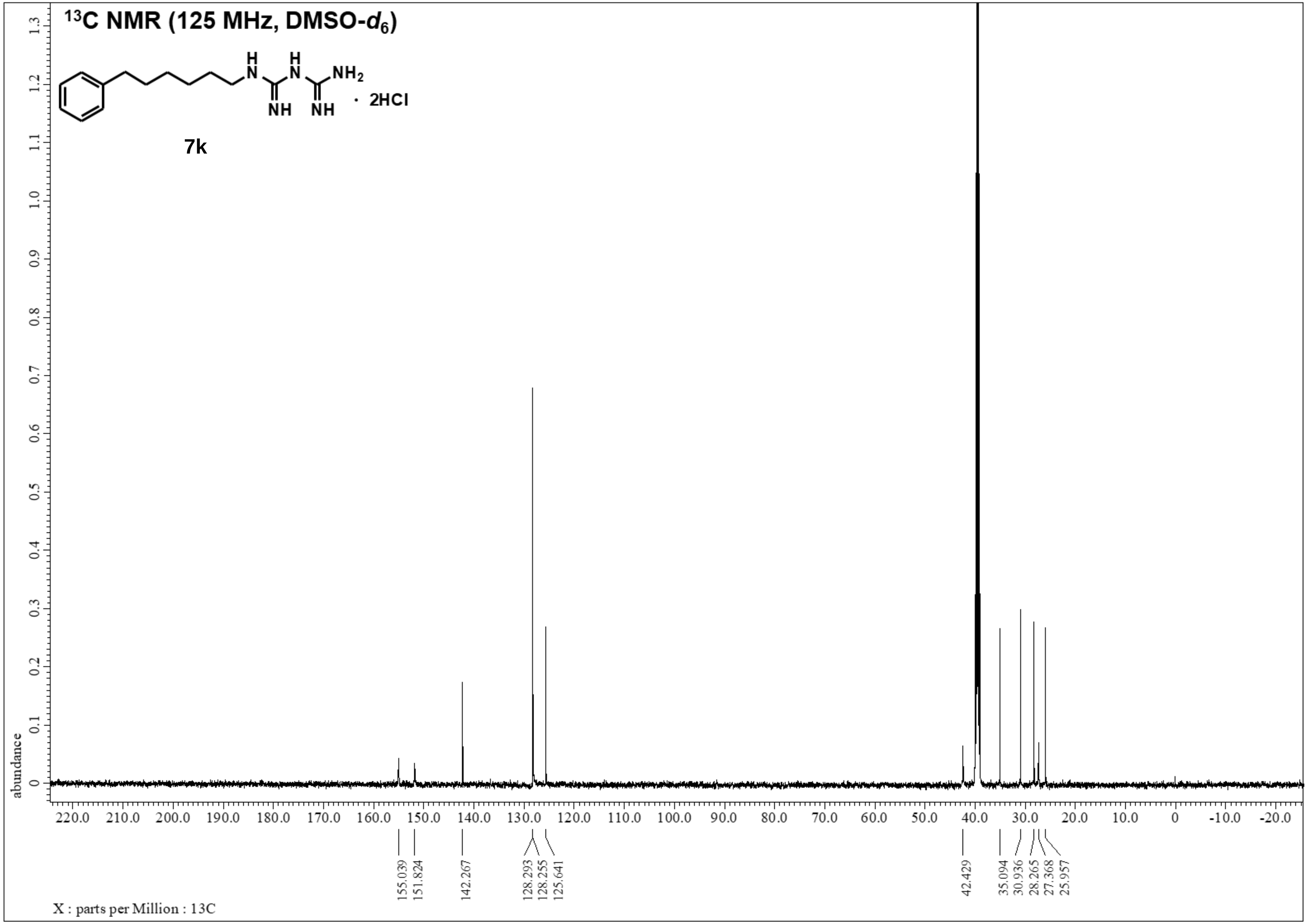

13C NMR (125 MHz, DMSO-d6)
NH2
NH
NH
· 2HCl
7k
abundance
X : parts per Million : 13C
155.039
151.824
142.267
128.293
128.255
125.641
42.429
35.094
30.936
28.265
27.368
25.957

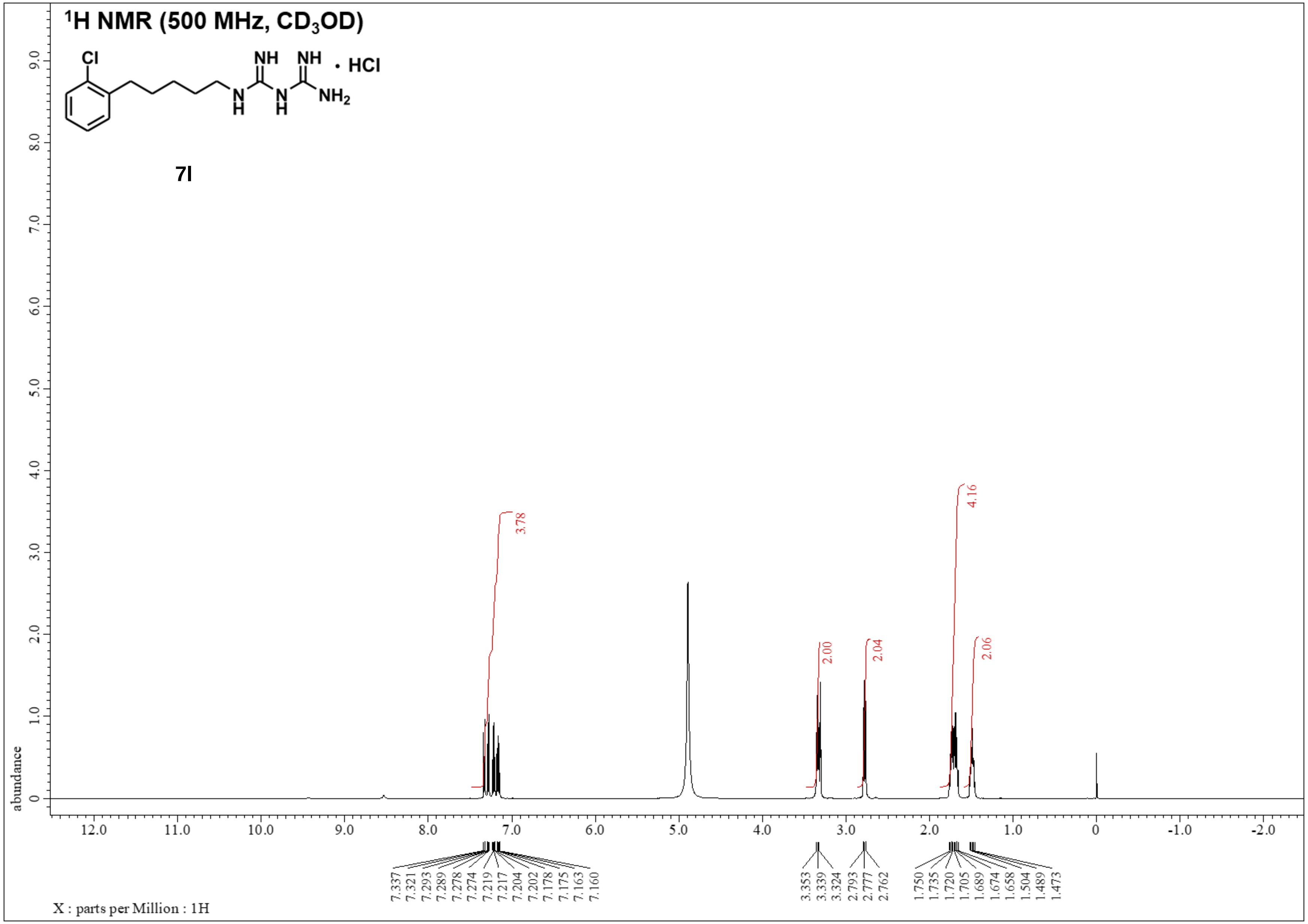

^{1}H NMR (500 MHz, CD_3OD)
Cl
NH
NH
· HCl
N
H
N
H
NH2
7l
3.78
4.16
2.00
2.04
2.06
abundance
X : parts per Million : 1H
7.337
7.321
7.293
7.289
7.278
7.274
7.219
7.217
7.204
7.202
7.178
7.175
7.163
7.160
3.353
3.339
3.324
2.793
2.777
2.762
1.750
1.735
1.720
1.705
1.689
1.674
1.658
1.504
1.489
1.473

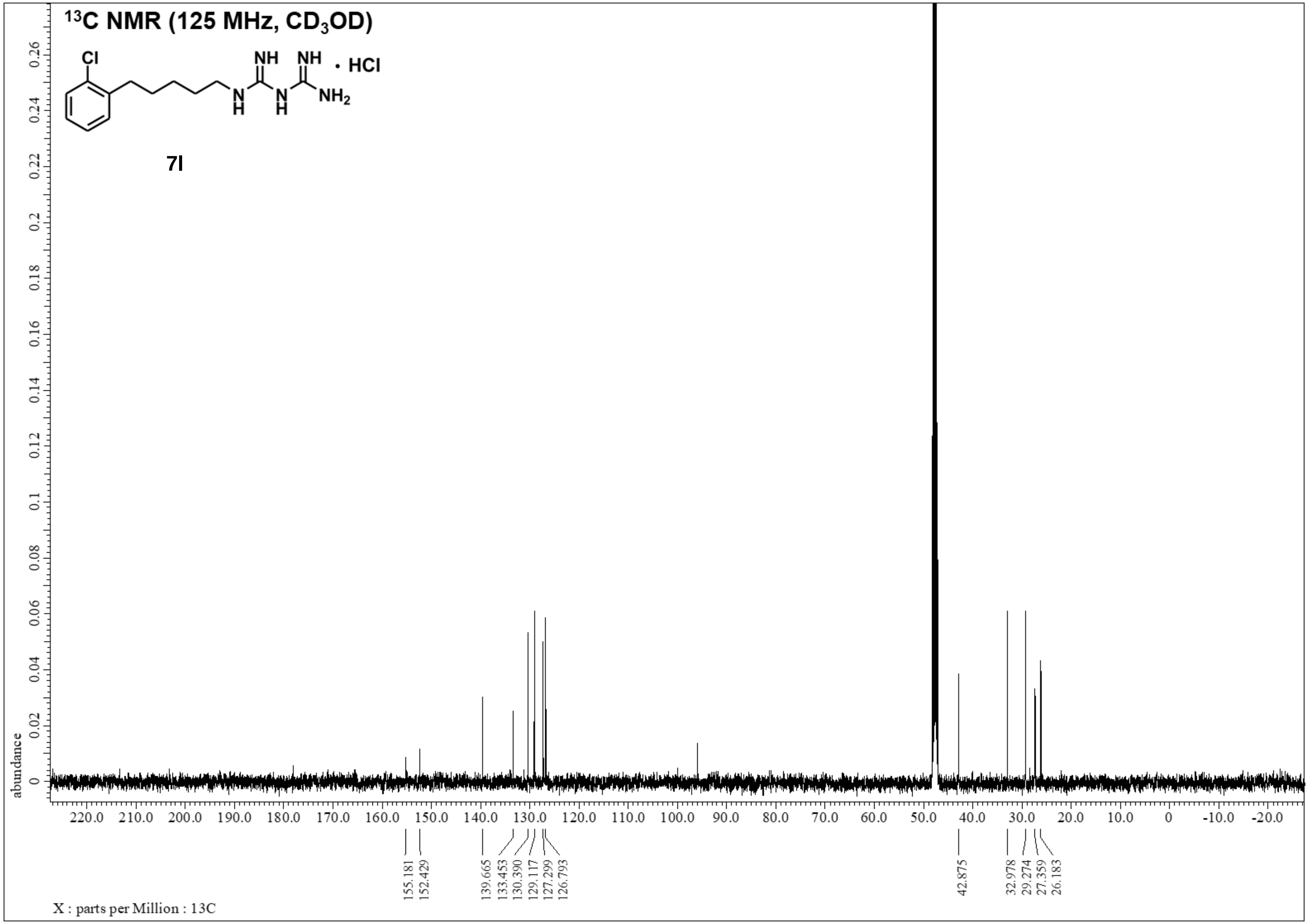

13C NMR (125 MHz, CD3OD)
Cl
NH
NH
· HCl
N
H
N
H
NH2
7l
abundance
X : parts per Million : 13C
155.181
152.429
139.665
133.453
130.390
129.117
127.299
126.793
42.875
32.978
29.274
27.359
26.183

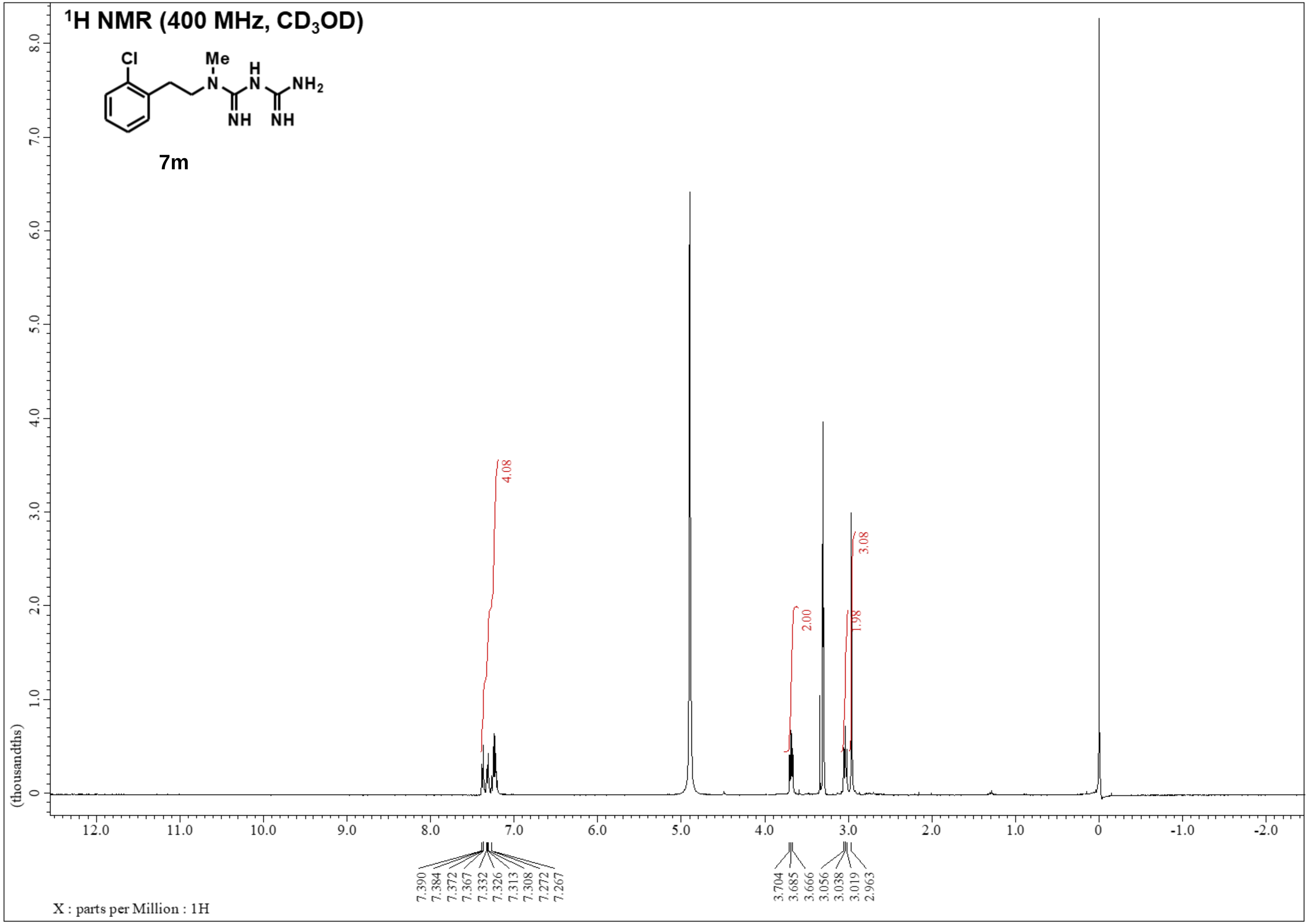

^{1}H NMR (400 MHz, CD$_3$OD)
Cl
Me
N
H
N
NH$_2$
NH
NH
7m
4.08
2.00
1.98
3.08
(thousandths)
X : parts per Million : 1H
7.390
7.384
7.372
7.367
7.332
7.326
7.313
7.308
7.272
7.267
3.704
3.685
3.666
3.056
3.038
3.019
2.963

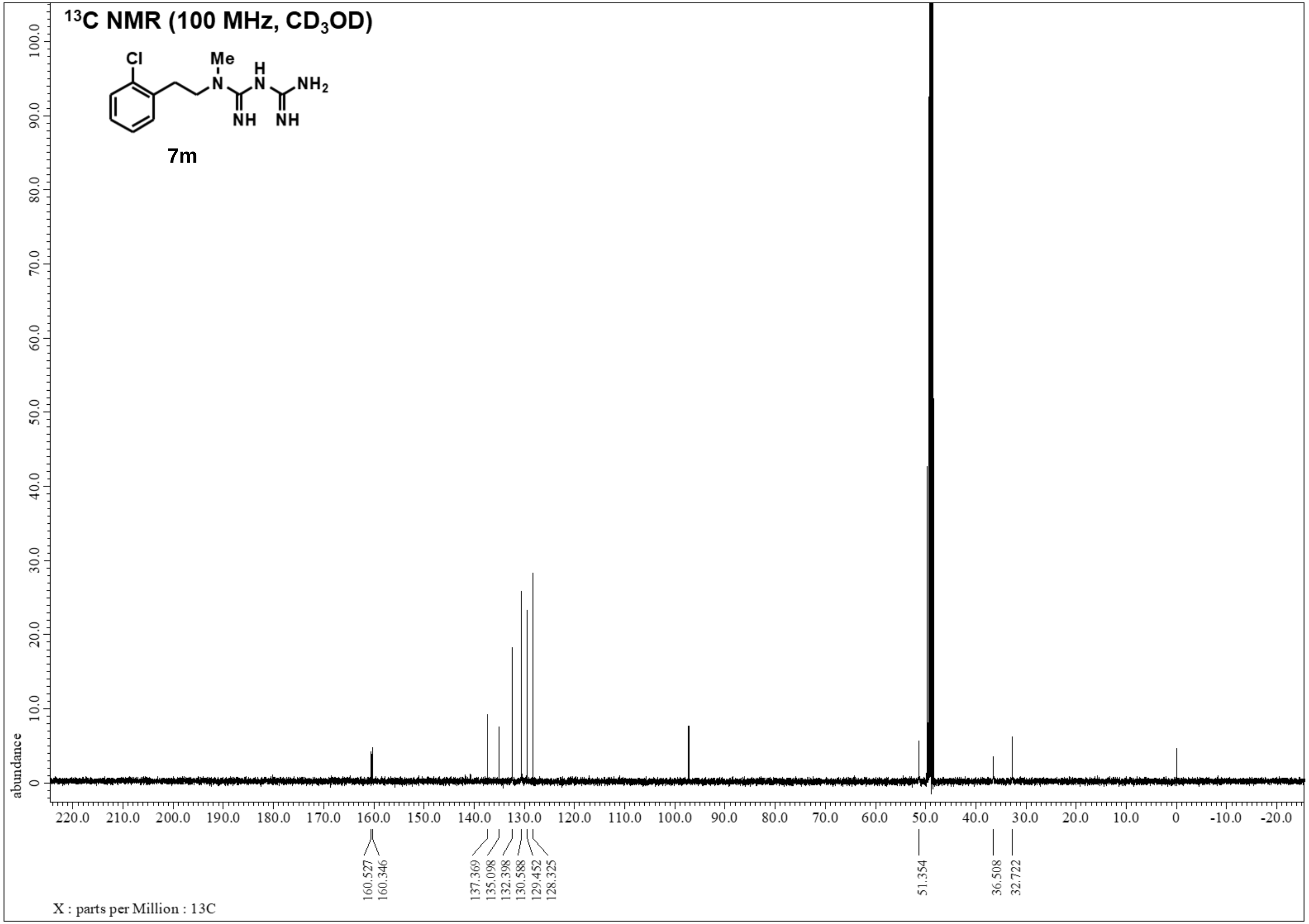

^{13}C NMR (100 MHz, CD$_3$OD)
Cl
Me
H
N
NH$_2$
NH
NH
7m
abundance
160.527
160.346
137.369
135.098
132.398
130.588
129.452
128.325
51.354
36.508
32.722
X : parts per Million : 13C

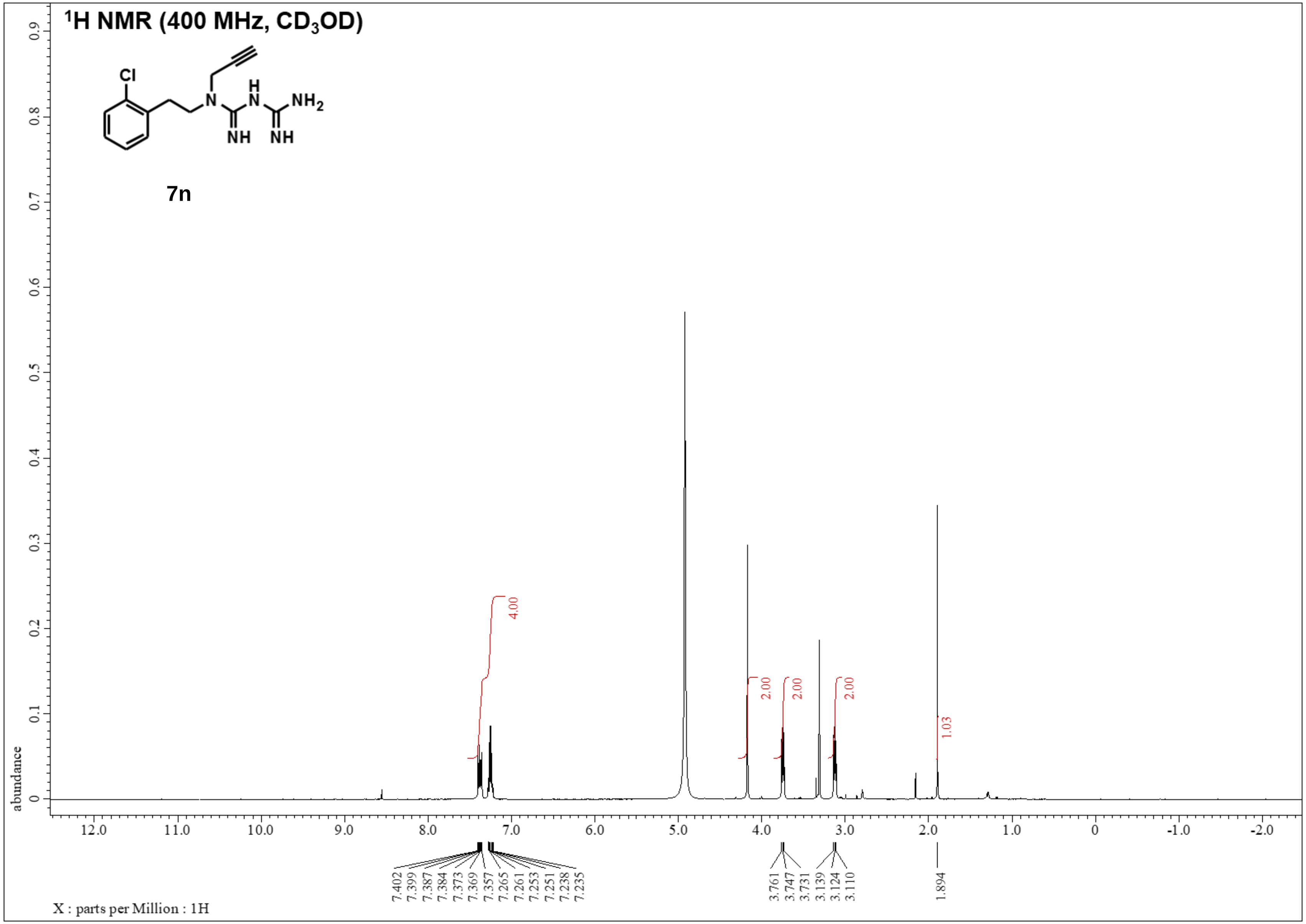

¹H NMR (400 MHz, CD3OD)
Cl
N
H
N
NH2
NH
NH
7n
4.00
2.00
2.00
2.00
1.03
7.402
7.399
7.387
7.384
7.373
7.369
7.357
7.265
7.261
7.253
7.251
7.238
7.235
3.761
3.747
3.731
3.139
3.124
3.110
1.894
abundance
X : parts per Million : 1H

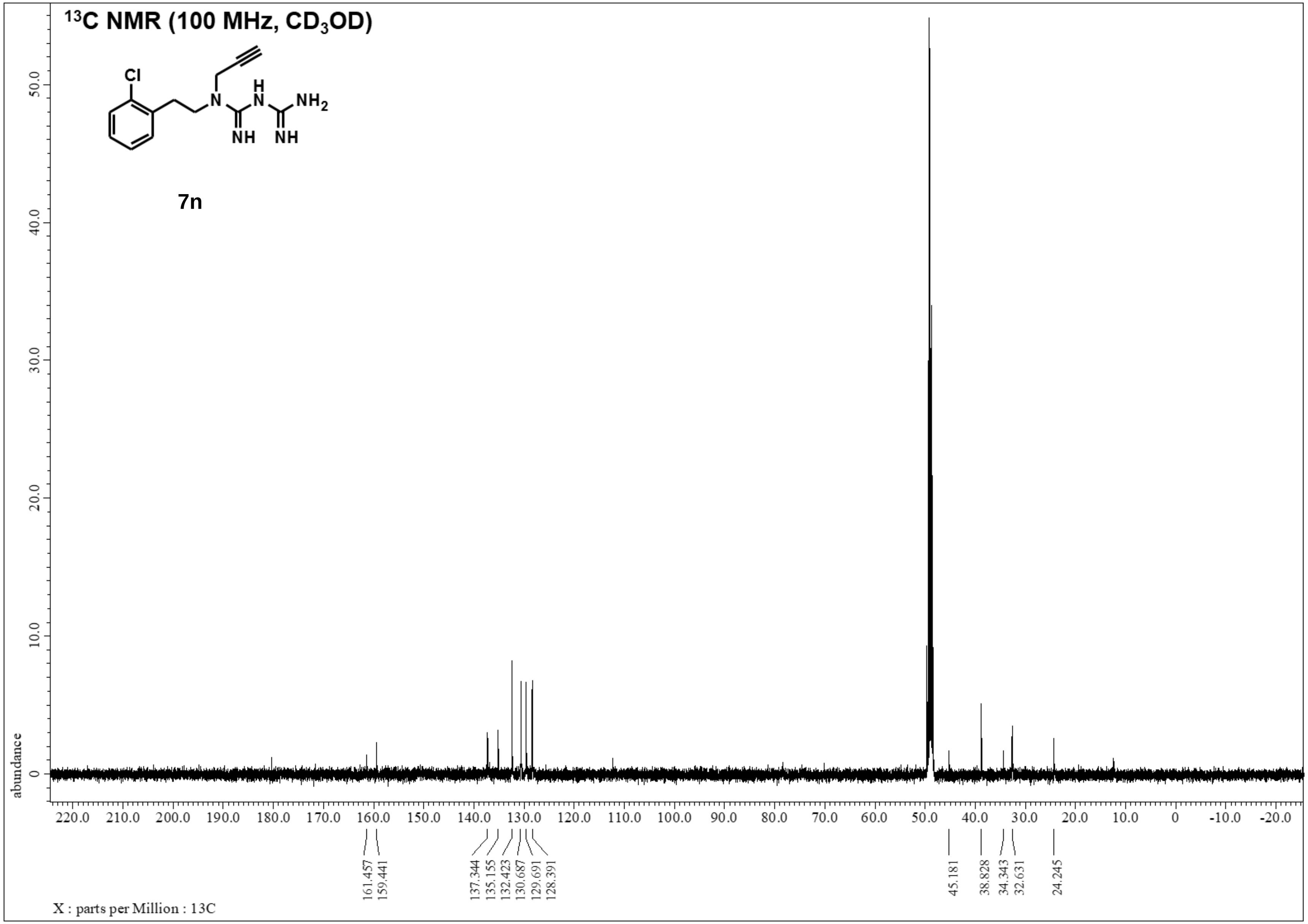

13C NMR (100 MHz, CD3OD)
Cl
N
H
N
NH2
NH
NH
7n
abundance
161.457
159.441
137.344
135.155
132.423
130.687
129.691
128.391
45.181
38.828
34.343
32.631
24.245
X : parts per Million : 13C

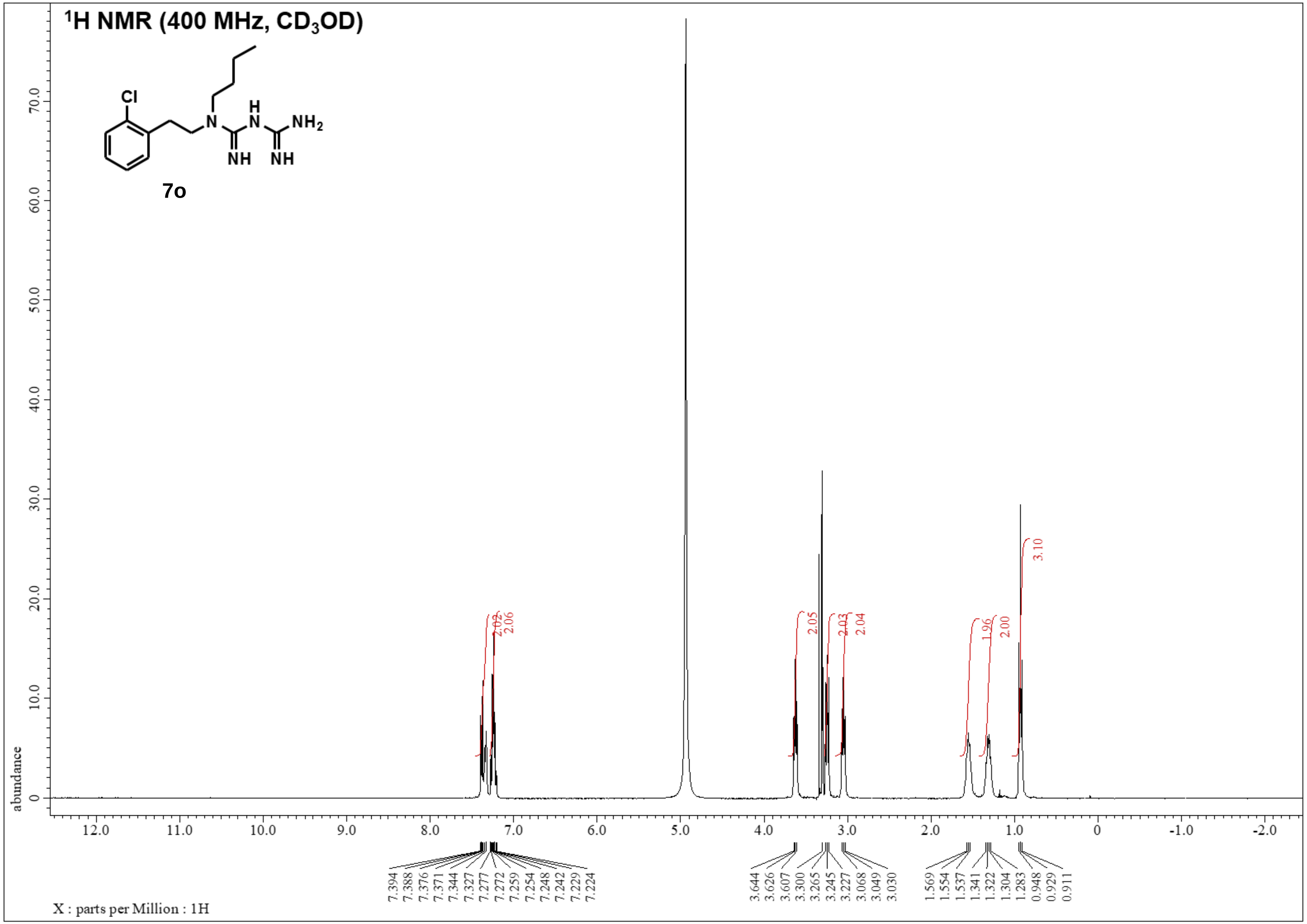

1H NMR (400 MHz, CD3OD)
Cl
N
H
N
NH2
NH
NH
7o
abundance
X : parts per Million : 1H
12.0 11.0 10.0 9.0 8.0 7.0 6.0 5.0 4.0 3.0 2.0 1.0 0 -1.0 -2.0
0 10.0 20.0 30.0 40.0 50.0 60.0 70.0
2.02 2.06 2.05 2.03 2.04 1.96 2.00 3.10
7.394 7.388 7.376 7.371 7.344 7.327 7.277 7.272 7.259 7.254 7.248 7.242 7.229 7.224
3.644 3.626 3.607 3.300 3.265 3.245 3.227 3.068 3.049 3.030
1.569 1.554 1.537 1.341 1.322 1.304 1.283 0.948 0.929 0.911

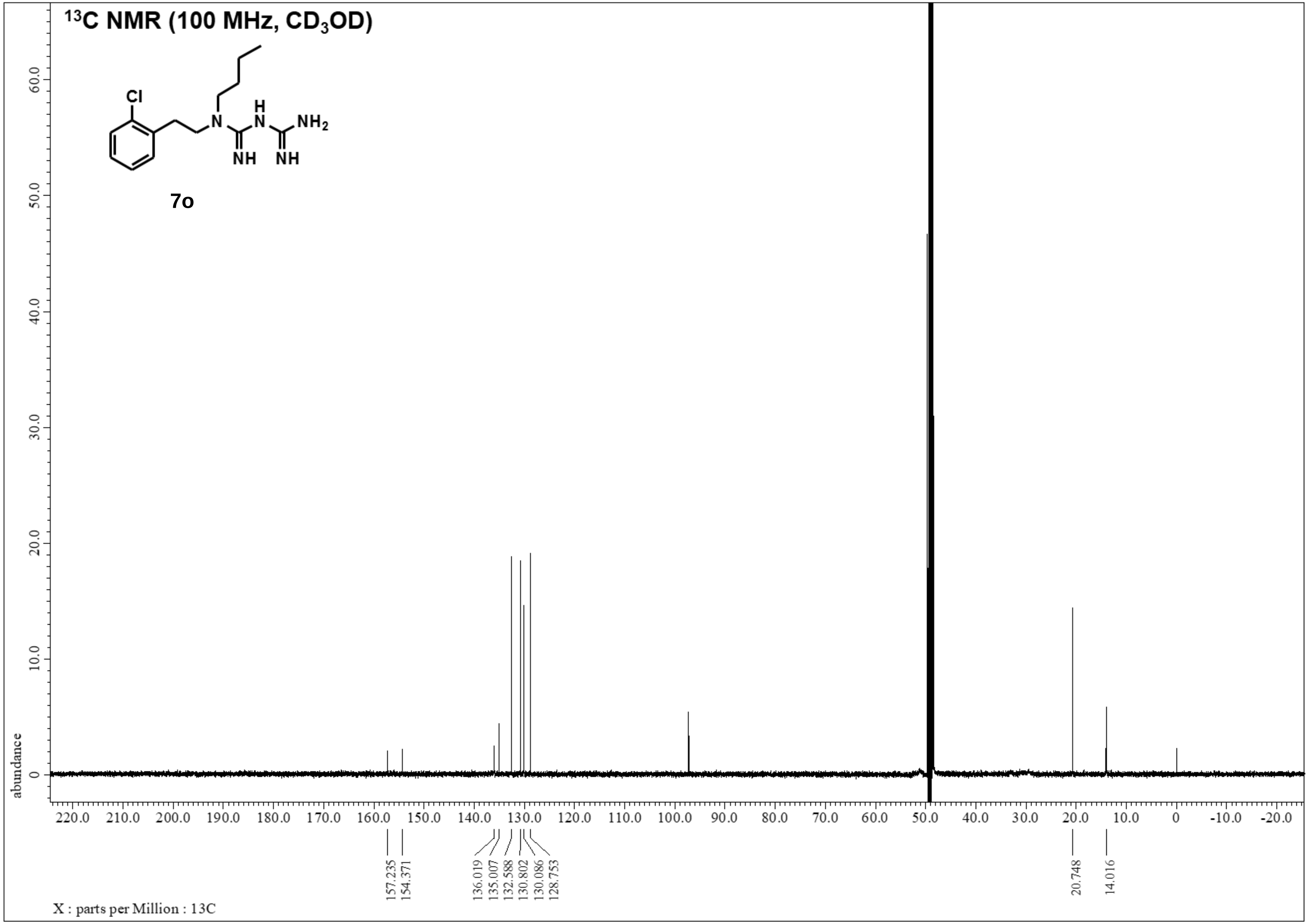

13C NMR (100 MHz, CD3OD)
Cl
H
N
N
NH2
NH
NH
7o
abundance
X : parts per Million : 13C
157.235
154.371
136.019
135.007
132.588
130.802
130.086
128.753
20.748
14.016

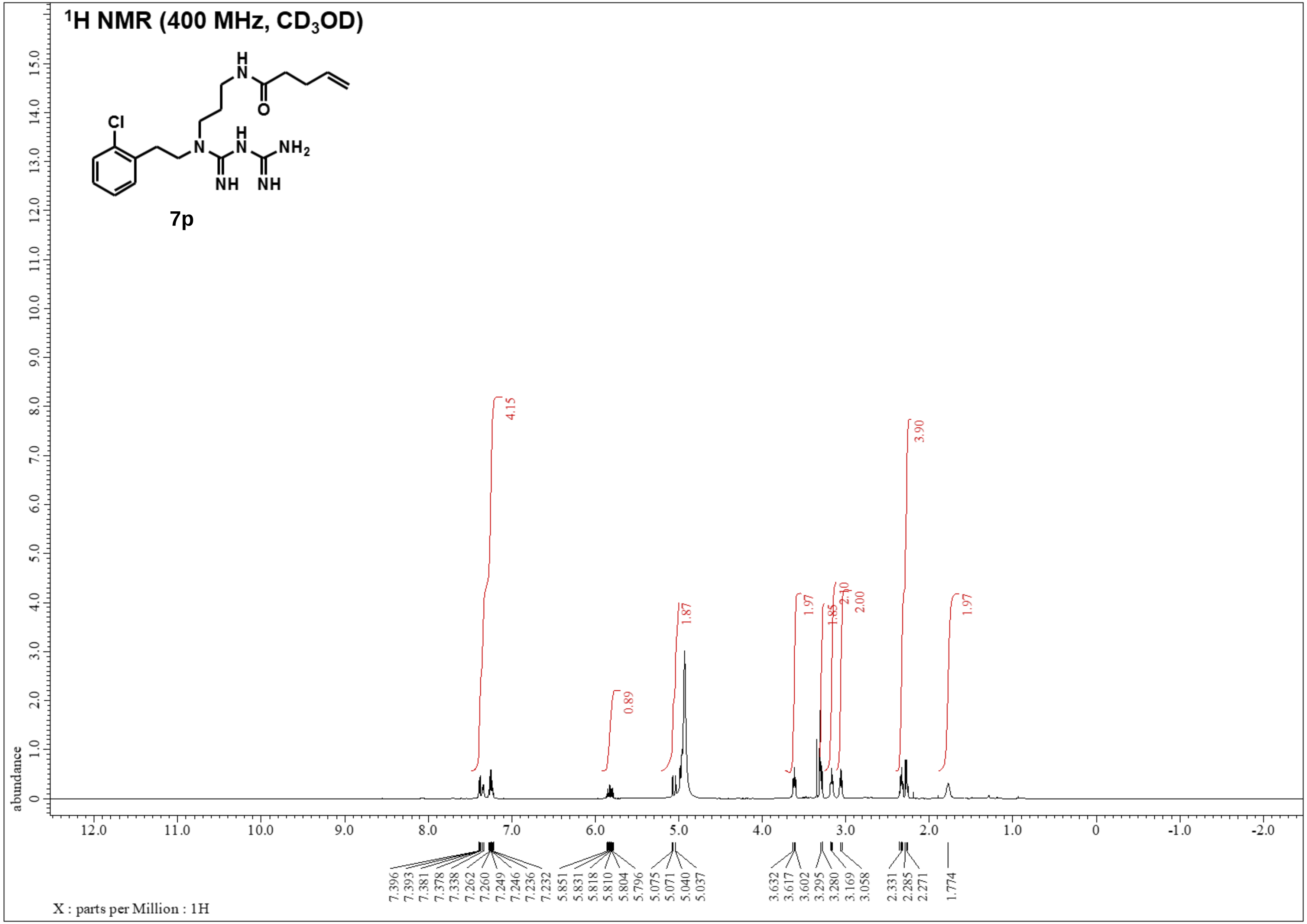

1H NMR (400 MHz, CD3OD)
7p
X : parts per Million : 1H
abundance

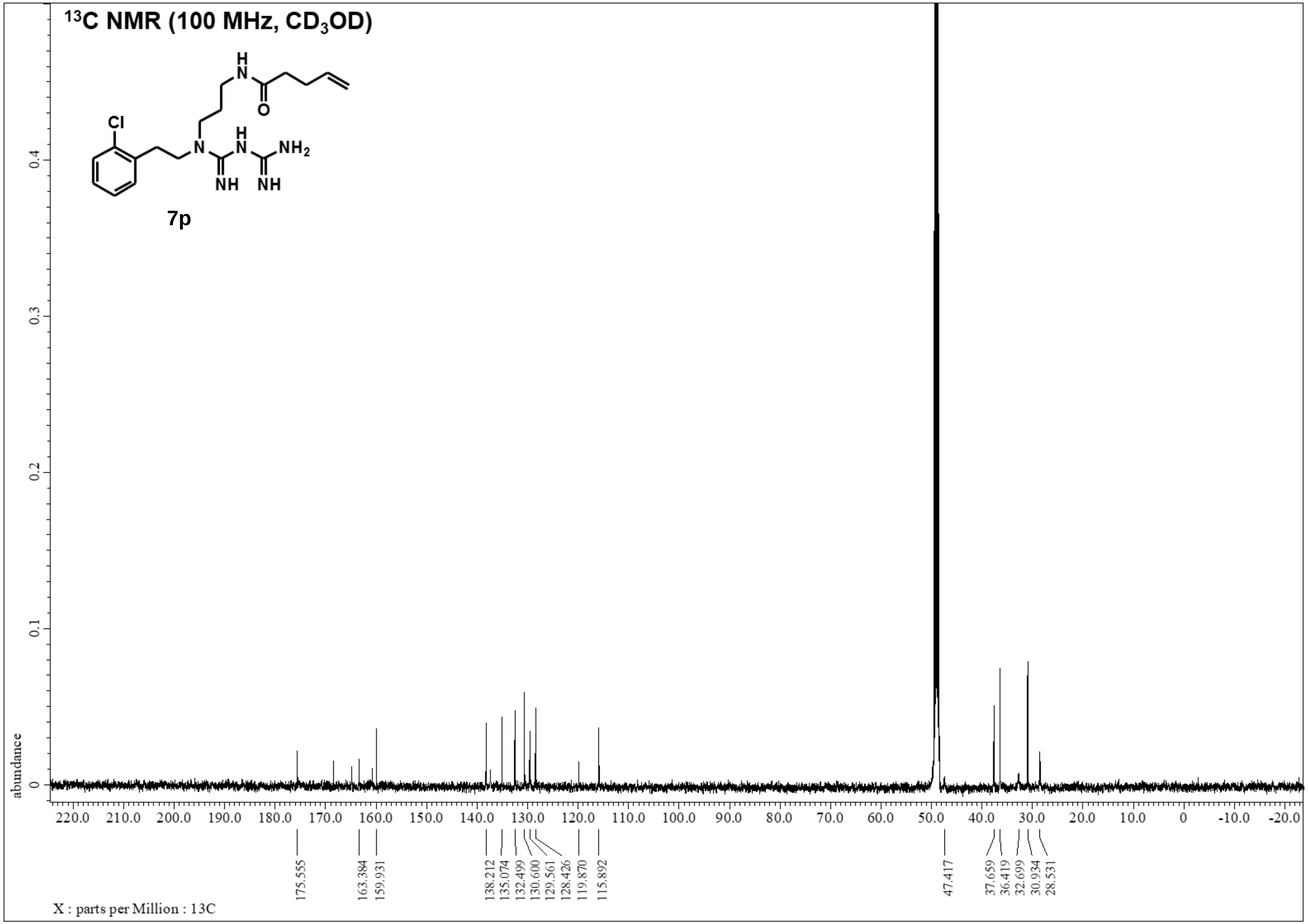
13C NMR (100 MHz, CD3OD)
7p
abundance
X : parts per Million : 13C
175.555
163.384
159.931
138.212
135.074
132.499
130.600
129.561
128.426
119.870
115.892
47.417
37.659
36.419
32.699
30.934
28.531
